# Tissue-specific fibroblast lipid cues impose the rate of epithelial cancer invasion

**DOI:** 10.1038/s42255-026-01514-y

**Published:** 2026-04-27

**Authors:** Timothy Budden, Noah Palombo, Shilpa Gurung, Martha Gutteridge, Charlotte Russell, Jair Marques, Alex von Kriegsheim, Lyutong An, Catherine Harwood, Luisa Motta, Claus Jorgensen, Carlos López-Garcîa, Caroline Gaudy-Marqueste, Kevin Harrington, Malin Pedersen, Ben O’Leary, Antonio Rullan, Amaya Virós

**Affiliations:** 1 Skin Cancer and Ageing Lab, Cancer Research UK Manchester Institute, University of Manchester, Manchester, UK; 2 Department of Molecular and Clinical Cancer Medicine, University of Liverpool, Liverpool, UK; 3 CRUK Scotland Centre, Institute of Genetics and Cancer, The University of Edinburgh, Edinburgh, UK; 4 Centre for Cell Biology and Cutaneous Research, Blizard Institute, Faculty of Medicine and Dentistry, Queen Mary University of London, London, UK; 5 Department of Histopathology, Salford Royal Hospital, Northern Care Alliance, Manchester, UK; 6 Systems Oncology, Cancer Research UK Manchester Institute, Manchester, UK; 7 North Wales Medical School, University of Bangor, Bangor, UK; 8 Aix-Marseille University, APHM, Hôpital Timone, Service de Dermatologie et de Cancérologie cutanée, Marseille, France; 9 Head and Neck Unit, The Royal Marsden NHS Foundation Trust, London, UK; 10 Targeted Therapy Team, The Institute of Cancer Research, London, UK; 11 The Institute of Cancer Research, National Institute of Health Research Biomedical Research Centre, London, UK; 12 Evolution and Translational Genomics Team, The Institute of Cancer Research, London, UK; 13 Institute of Immunity and Transplantation, University College London, London, UK; 14 NIHR Manchester Biomedical Research Centre, Manchester, UK; 15 Department of Dermatology, Salford Royal Hospital, Northern Care Alliance, Manchester, UK

**Keywords:** Metabolism, Head and neck cancer, Non-small-cell lung cancer, Squamous cell carcinoma, Cancer microenvironment

## Abstract

Squamous cell carcinomas (SCCs) originate in epithelial tissues of older individuals who have been exposed to environmental carcinogens. Despite overlapping clinical hallmarks, SCCs from different anatomic sites have different prognoses. Here we show that fibroblasts confer site-specific cues that determine SCC proliferation and invasion. Oral and lung fibroblasts have distinct lipid metabolism, transferring unique lipids to SCC cells that promote epithelial-to-mesenchymal transition, and oral and lung SCC invasion. Whereas oral fibroblasts transfer sphingomyelins, which activate the ceramide–sphingosine-1-phosphate–STAT3 pathway and promote oral SCC invasion, lung fibroblasts transfer triglycerides to lung SCCs, thereby triggering cholesterol synthesis and invasion, which is associated with poor survival. By contrast, dermal fibroblasts are lipid poor, and cutaneous SCC is less invasive. Our data indicate that targeting fibroblast lipid synthesis and SCC lipid uptake or breakdown inhibits oral and lung epithelial cancer invasion.

## Abstract (web summary)

Tissue-specific fibroblast lipid cues are shown to significantly influence the invasion rate of squamous cell carcinomas (SCCs), with oral and lung fibroblasts promoting aggressive cancer behaviour through distinct lipid metabolic pathways, while dermal fibroblasts, being lipid poor, limit cutaneous SCC invasion.

## Main

SCCs arise from epithelial tissues that line the surfaces and cavities of the body, such as the skin, oral cavity, respiratory, genitourinary and digestive tracts^1^. Together, SCCs show the highest incidence of all cancers, accounting for 80–90% of all cases^2^.

Although SCCs are classified by anatomic location, they present overlapping epidemiological, histological and molecular hallmarks. Specifically, they are driven by environmental carcinogens, such as ultraviolet (UV) light, tobacco and alcohol, which increase SCCs at different sites by inducing DNA damage. This interaction with extrinsic carcinogens results in a high mutation burden and alterations in key tumour suppressor genes and oncogenes (*RAS*, *TP53*, *CDKN2A* and *NOTCH1*/*NOTCH2*), which are common in most SCCs^3,4^. Interestingly, despite the shared aetiopathology, SCCs from different organs have significantly different prognoses. Cutaneous SCCs arise from UV-damaged skin, and metastases are rare (1.2–5%)^5^. In contrast, SCCs arising from internal cavities and organs, such as oral SCC and lung SCC, are highly metastatic and deadly^3^.

Epithelial architecture and function are supported by the adjacent connective tissue or stroma, and stromal fibroblasts determine epithelial cell identity and differentiation^6,7^. Importantly, fibroblasts from different sites express organ-specific instructions reflecting developmental programmes that are maintained through adulthood^8–11^, implicating tissue-specific fibroblast programmes in tissue-specific homeostasis. Critically, the stroma contributes to SCC progression^12,13^, and fibroblasts impact tumour initiation, progression and metastasis^14,15^. Therefore, we investigated whether site-specific fibroblasts confer site-specific cues to SCCs that impact invasion and survival.

We found that fibroblasts from the oral cavity and the lung are lipid rich and transfer lipids to adjacent SCC cells. Fibroblast lipids taken up by SCC cells promote epithelial-to-mesenchymal transition (EMT) and promote SCC invasion. In contrast, cutaneous fibroblasts are lipid poor and transfer few lipids to SCCs, which limits invasion. Oral fibroblasts synthesize and transfer sphingolipids (sphingomyelins or SMs), which are signalling lipids, to drive oral SCC invasion. In contrast, lung fibroblasts synthesize and transfer triglycerides (TGs), which promote aggressive behaviour in lung SCCs. We show that oral and lung fibroblast lipids drive SCC invasion independently of tumour-intrinsic SCC biology and SCC anatomic subtype. Mechanistically, targeting fibroblast lipid synthesis, lipid uptake or breakdown in SCC cells inhibits tumour invasion. Our work shows that site-specific fibroblast cues determine SCC progression at distinct anatomic sites, which correlates with site-specific SCC mortality.

## Results

### Oral and lung fibroblasts increase SCC invasion

**Fig. 1:**
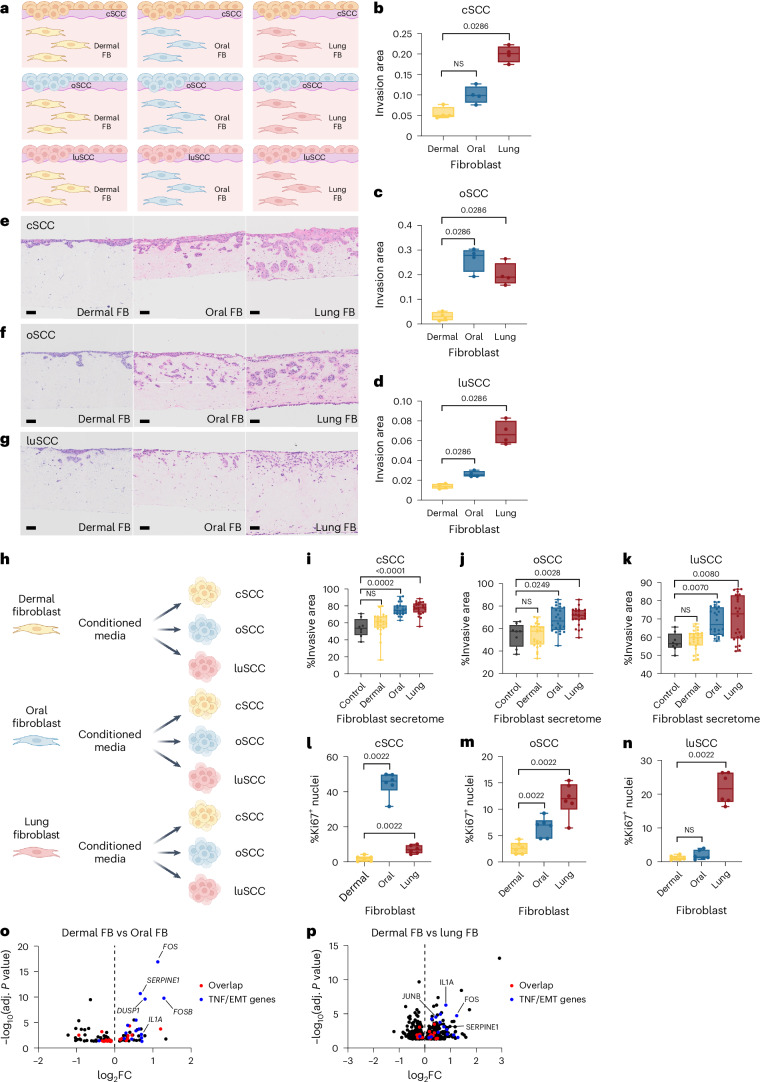
Oral and lung fibroblasts increase SCC invasion. **a**, Schematic of organotypic invasion models combining SCC and tissue fibroblasts (FBs). **b**–**d**, cSCC cell line (IC19) (**b**), oSCC cell line (FADU) (**c**) and luSCC cell line (SKMES1) (**d**) invasion in organotypic constructs with dermal (yellow), oral (blue) and lung (red) FBs (two-sided Mann–Whitney *U-*test, *n* = 4 counts in two independent constructs). **e**–**g**, Representative images of cSCC IC19 (**e**), oSCC UMSCC01 (**f**) and luSCC SKMES1 (**g**) invasion in organotypic model with dermal (left), oral (middle) and lung (right) FBs (scale bars, 100 µm). **h**, Schematic of spheroid invasion model with tissue FB secretomes. **i**–**k**, Spheroid invasion of cSCC (IC1, IC19) (**i**), oSCC (FADU, UMSCC01) (**j**) and luSCC (SKMES1, H520) (**k**) treated with control media (grey; *n* = 8 spheroids in two independent experiments) or FB secretomes (yellow, dermal; blue, oral; red, lung; *n* = 24 spheroids across two experiments, Kruskal–Wallis test, Dunn’s multiple comparisons). **l**–**n**, Quantification of proliferating cSCC (IC19) (**l**), oSCC (FADU) (**m**) and luSCC (H520) (**n**) in organotypic models with dermal (yellow), oral (blue) and lung (red) FBs (two-sided Mann–Whitney *U-*test, *n* = 6 quantifications over two independent organotypic constructs). **o**, Volcano plot of differentially expressed genes in SCC cell lines treated with dermal FB secretomes (log_2_FC < 0) or oral FB secretomes (log_2_FC > 0). Differential expression analysis was performed using a negative binomial generalized linear model and two-sided Wald test. *P* values were adjusted for multiple comparisons using the Benjamini–Hochberg method (FDR). Red indicates genes overlapping with differentially expressed genes by lung FB secretome; blue indicates genes related to TNF and EMT signalling. **p**, Volcano plot of differentially expressed genes in SCC cell lines treat with dermal FB secretomes (log_2_FC < 0) or lung FB secretomes (log_2_FC > 0). Differential expression analysis was performed using a negative binomial generalized linear model and two-sided Wald test. *P* values were adjusted for multiple comparisons using the Benjamini–Hochberg method (FDR). Red indicates genes overlapping with differentially expressed genes by oral FB secretome. Blue indicates genes related to TNF and EMT signalling. Box plots represent minimum to maximum values (error bars), the box represents 25th and 75th percentiles, and the line represents the median value. All data points are displayed. Panels created in BioRender: **a**, Budden, T. https://biorender.com/csfpg9x (2026); **h**, Budden, T. (2026) https://BioRender.com/b0eod5w (2026). NS, not significant. Source data

**Extended Data Fig. 1:**
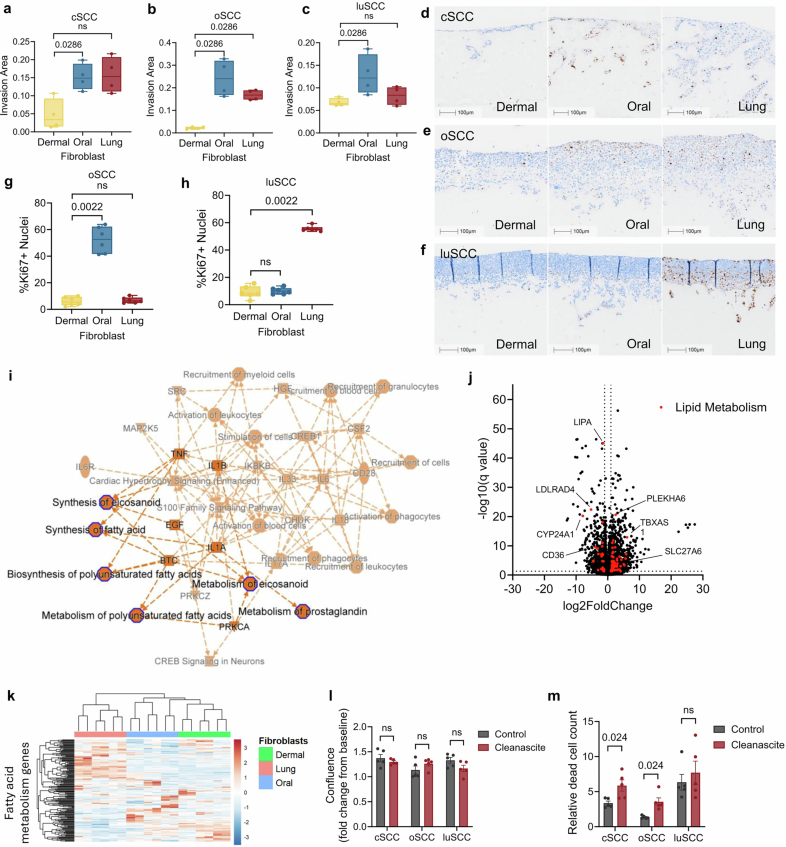
Lipid cues from stromal fibroblasts impose SCC invasion and proliferation. **a**, cSCC cell line (IC1) invasion in organotypic models with dermal (yellow), oral (blue), lung (red) fibroblasts (two-sided Mann Whitney U, ns: non-significant, n = 4 quantifications, two independent constructs). **b**, oSCC cell line (UMSCC01) invasion in organotypic models with dermal (yellow), oral (blue), lung (red) fibroblasts (two-sided Mann Whitney U, n = 4 quantifications two independent constructs). **c**, luSCC cell line (H520) invasion in organotypic models with dermal (yellow), oral (blue), and lung (red) fibroblasts (two-sided Mann Whitney U, n = 4 quantification over two independent organotypics). **d**, Representative images of cSCC IC19, **e**, oSCC FADU, **f**, luSCC H520 proliferation (Ki67, brown) in organotypic model with dermal (left), oral (middle), lung (right) fibroblasts, scale=100 µm. **g**, oSCC (UMSCC01) proliferation in organotypic construct with dermal (yellow), oral (blue), lung (red) fibroblasts (two-sided Mann Whitney U, ns: non-significant, n = 6 quantifications, two independent organotypics). **h**, luSCC (SKMES1) proliferation in organotypic constructs with dermal (yellow), oral (blue), lung (red) fibroblasts (two-sided Mann Whitney U, ns: non-significant, n = 6 quantifications, two independent organotypics). **i**, Ingenuity pathway analysis graphical summary representing most significant differentially expressed pathways, regulators and gene functions in dermal and oral/lung fibroblasts. Highlighted pathways and molecules represent lipid metabolism enrichment. **j**, Volcano plot of differential gene expression between dermal and oral/lung fibroblasts, differential expression analysis was performed using a negative binomial generalized linear model and two-sided Wald test, p-values were adjusted for multiple comparisons using the Benjamini–Hochberg method (FDR), red: lipid metabolism genes (adjusted p-value < 0.05). **k**, Unsupervised heatmap clustering of fatty acid metabolism genes in fibroblasts (dermal: green, oral: blue, lung: red), colour scale: red: high expression, blue: low expression. **l**, Confluence of SCC cell lines treated with fibroblast secretomes (control, grey) or Cleanascite treated secretomes (red), represented as a fold change from baseline (n = 5 independent measurements per cell line, two-sided Mann Whitney U. ns: non-significant). **m**, Dead cell counts of SCC cell lines treated with fibroblast secretomes (control, grey) or Cleanascite treated secretomes (red) normalised to cell confluence (two-sided Mann Whitney U, n = 5 independent measurements per cell line, ns: non-significant). Boxplots: minimum to maximum values (error bars), box: 25th and 75th percentiles; line: median. Bar plots display mean ± standard error of the mean. Source data

To assess whether tissue-specific fibroblasts or SCC subtype drive SCC invasion, we built organotypic three-dimensional matrices with adult dermal, oral or lung fibroblasts, and then seeded the fibroblast matrices with cutaneous (cSCC), oral (oSCC) or lung (luSCC) cells (Fig. 1a). This revealed that all SCC subtypes (cSCC, oSCC and luSCC) were more invasive in matrices formed with either oral or lung fibroblasts (Fig. 1b–g and Extended Data Fig. 1a–c) and, strikingly, cSCC, oSCC and luSCC invasion was significantly reduced on dermal fibroblast matrices. These data indicate that fibroblast biology impacts SCC invasion, and lung and oral fibroblasts confer invasive cues to adjacent SCC cells. Oral and lung, but not dermal fibroblasts, increase oral, cutaneous and luSCC invasion in three dimensions.

To determine if fibroblasts modulate SCC invasion by modifying the extracellular matrix or by secreting factors that drive SCC invasion, we embedded spheroids of cSCC, oSCC and luSCC subtypes in equal concentrations of collagen, and cultured each SCC subtype with lung, oral or dermal fibroblast secretomes (conditioned media; Fig. 1h). This revealed that cSCC, oSCC and luSCC spheroids were significantly more invasive when exposed to oral or lung fibroblast secretomes, but not dermal fibroblast secretomes (Fig. 1i–k). Notably, we observed that lung fibroblast secretomes conferred the highest level of invasion. These data show that secreted oral and lung fibroblast factors drive SCC invasion. Furthermore, the rate of proliferation across all SCC constructs was notably increased when cSCC, oSCC and luSCC cells were seeded over oral or lung fibroblast matrices (Fig. 1l–n and Extended Data Fig. 1d–h). Altogether, this demonstrates that oral and lung fibroblasts secrete factors that drive SCC proliferation and invasion, in contrast to dermal fibroblasts, which do not confer invasive properties to SCC cells. Critically, these data correlate with patient outcomes, as oral and lung SCCs have significantly higher rates of mortality (≈45–80% 5-year survival rate^16^) compared to cSCC (≈2% mortality)^16,17^.

## Oral and lung fibroblasts induce aggressive SCC transcriptional programmes

To investigate how oral and lung fibroblasts increase SCC invasion, we studied the transcriptional changes in cSCC, oSCC and luSCC cells after dermal, oral or lung fibroblast secretome exposure. We compared the gene expression in SCCs exposed to oral and lung fibroblasts to the gene expression in SCCs exposed to dermal fibroblasts. We found that lung fibroblast exposure induced the most differentially expressed genes in SCC cells (1,001 genes, adjusted *P* value < 0.05; Supplementary Table 1) compared to oral fibroblasts (100 genes, adjusted *P* value < 0.05; Supplementary Table 2 and Fig. 1o,p). A total of 36 genes were significantly differentially expressed by both oral and lung fibroblasts. Gene-set analysis revealed enrichment for EMT and tumour necrosis factor (TNF) signalling pathway genes, including interleukin-1 (*IL1A*, *IL1B*), AP-1 (*FOS*, *FOSB*, *JUN*, *JUNB*) and TNF–nuclear factor (NF)-κB signalling genes, which are known to promote metastasis and stemness in SCCs^18–22^. Thus, oral and lung fibroblasts secrete factors that induce EMT in exposed SCC cells.

## Oral, lung and dermal fibroblasts have unique lipid metabolism profiles that impact SCC invasion

**Fig. 2:**
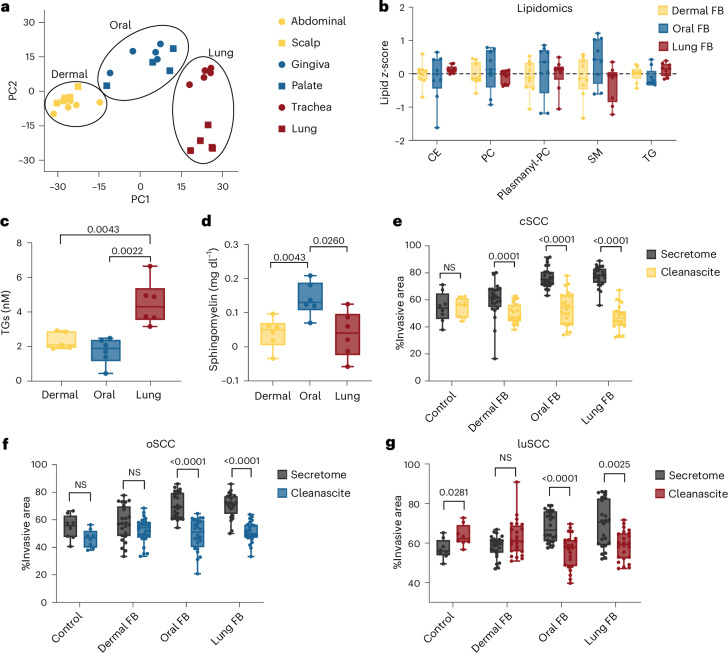
Fibroblasts from different anatomic sites present unique lipid metabolism profiles. **a**, PCA of FB RNA sequencing by anatomic site. **b**, Lipidomics of FB secretomes. Data represent average *z*-score for each lipid family. Error bars represent the s.e.m. *n* = 9, triplicate secretomes of three independent cell lines per condition (CE, cholesterol ester; PC, phosphatidylcholine). **c**, TG quantification in FB secretomes by assay (two-sided Mann–Whitney *U-*test, *n* = 6 duplicate measurements of three independent FB secretomes). **d**, SM quantification in dermal (yellow), oral (blue) and lung (red) FB secretomes by assay (two-sided Mann–Whitney *U-*test, *n* = 6, duplicate measurements of three independent FB secretomes. **e**, Spheroid invasion of cSCC lines with FB secretomes (grey) and secretomes stripped of lipids with Cleanascite (yellow; two-sided Mann–Whitney *U-*test, control *n* = 8 replicate spheroids, FB secretomes *n* = 24 independent spheroids from two cell lines treated with three independent FB secretomes). **f**, Spheroid invasion of oSCC lines with FB secretomes (grey) and secretomes stripped of lipids with Cleanascite (blue; two-sided Mann–Whitney *U-*test, control *n* = 8 replicate spheroids, FB secretomes *n* = 24 independent spheroids from two cell lines treated with three independent FB secretomes). **g**, Spheroid invasion of luSCC lines with FB secretomes (grey) and secretomes stripped of lipids with Cleanascite (red; two-sided Mann–Whitney *U-*test, control *n* = 8 replicate spheroids, FB secretomes *n* = 24 independent spheroids from two cell lines treated with three independent FB secretomes). Box plots represent minimum to maximum values (error bars), the box represents 25th and 75th percentiles, and the line represents the median value. Data points are displayed. Bar plots show the mean ± s.e.m. Source data

To identify the secreted factors in oral and lung fibroblasts that drive SCC invasion, we first examined the transcriptomic profiles from healthy adult fibroblasts from the skin, the oral mucosa and the lung^8^. Principal component analysis (PCA) confirmed that fibroblasts cluster by anatomic site, with the first principal component separating dermal fibroblasts from oral and lung fibroblasts (Fig. 2a). Oral and lung fibroblasts drive SCC invasion, so we compared the differential gene expression of oral and lung fibroblasts (gingival, palate, trachea, lung) to dermal fibroblasts (scalp, abdomen). A total of 1,779 genes were significantly differentially expressed (false discovery rate (FDR)-adjusted *P* value < 0.05; Supplementary Table 3) with 1,198 upregulated in oral and lung fibroblasts (log_2_ fold change (FC) ≥ 1) and 581 upregulated in dermal fibroblasts (log_2_FC ≤ −1). Pathway analysis revealed lipid metabolic pathways were enriched in oral and lung fibroblasts compared to dermal fibroblasts (Extended Data Fig. 1i). To confirm lipid metabolism differentiates the oral, lung and dermal fibroblasts, we studied the transcriptional programmes in the in vitro fibroblast cell lines, which validated that lipid metabolic genes were differentially expressed in oral and lung fibroblasts (58 genes, log_2_FC ≥ 1, adjusted *P* < 0.05) compared to dermal fibroblasts (33 genes, log_2_FC ≤ −1, adjusted *P* value < 0.05; Extended Data Fig. 1j and Supplementary Table 3). Moreover, fatty acid metabolism genes distinguished dermal from oral and lung fibroblasts (Extended Data Fig. 1k).

We next hypothesized that the lipid transcriptional differences in oral, lung and dermal fibroblasts could translate to distinct lipid secretory profiles driving cancer^23–25^, so we performed lipidomic analyses. We compared the secretory lipid profile of adult fibroblasts from distinct anatomic sites (dermal, oral and lung), which revealed that oral and lung fibroblasts secreted more lipids than dermal fibroblasts; furthermore, oral, lung and dermal fibroblasts secreted distinct lipid species (Fig. 2b and Supplementary Table 4). Lung fibroblasts secreted the most TGs (Fig. 2c), while oral fibroblasts secreted the most SMs (Fig. 2d). We validated tissue-specific lipid patterns in mouse samples (Supplementary Fig. 1–3).

Cancer cells exploit lipid metabolism^26,27^, so we tested whether lipids secreted by fibroblasts drive SCC invasion. For this, we stripped lipids from fibroblast secretomes with Cleanascite, then exposed SCC spheroids to lipid-stripped secretomes. This established that lipid-stripped oral and lung fibroblast secretomes lost the ability to drive SCC invasion. Importantly, stripping lipids from dermal fibroblast secretomes had no impact on visceral SCC invasion (Fig. 2e–g), and Cleanascite had no negative impact on cell viability (Extended Data Fig. 1l,m). Thus, these data show that lipids secreted by oral and lung fibroblasts drive SCC invasion.

## Oral fibroblast SMs drive oSCC invasion

**Fig. 3:**
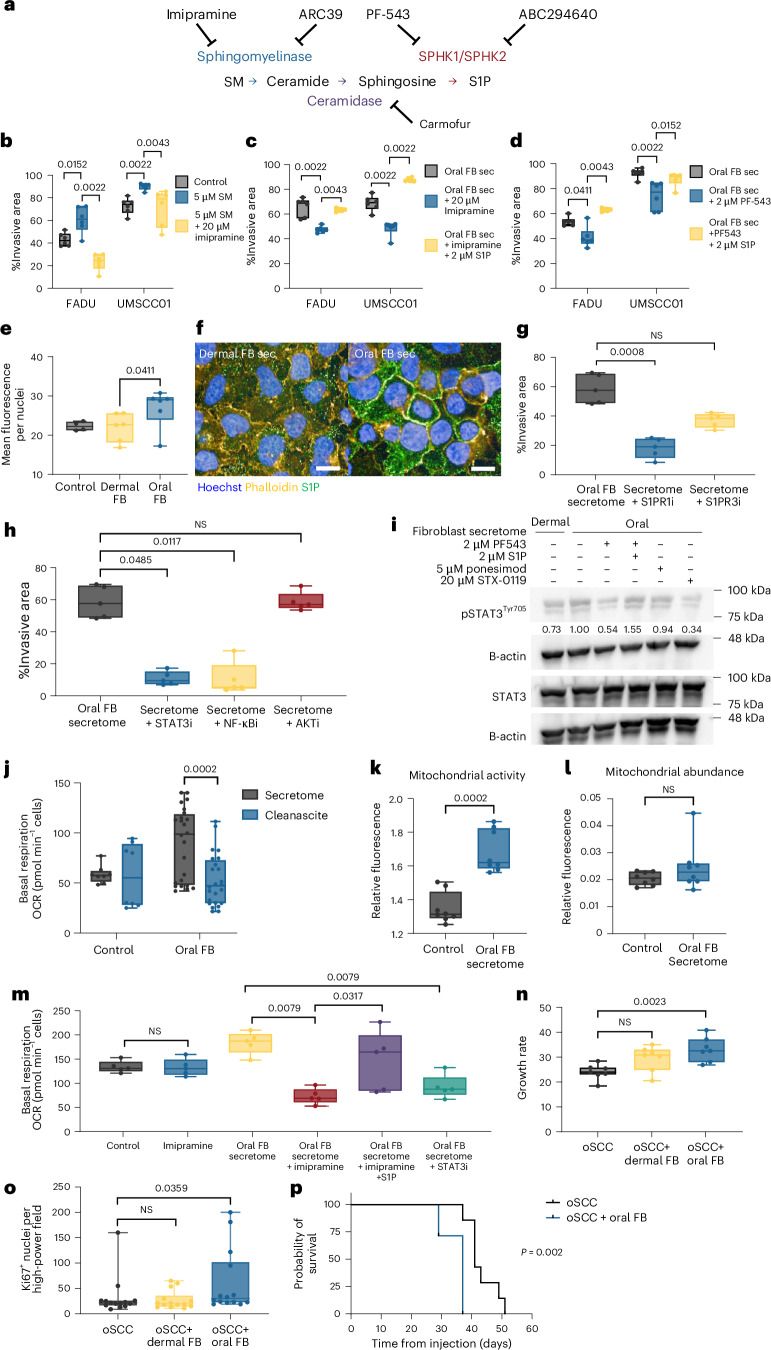
Oral fibroblasts secrete SMs to drive oSCC invasion. **a**, SM metabolic pathway and inhibitors targeting enzymes. **b**–**d**, Spheroid invasion of oSCC with control media (grey), 5 µM SM (blue) or 5 µM SM and 20 µM imipramine (yellow) (**b**), oral FB secretomes (grey), with 20 µM imipramine (blue) or 20 µM imipramine and 2 µM S1P (yellow) (**c**) and oral FB secretomes (grey), with 2 µM PF543 (blue) or 2 µM PF543 and 2 µM S1P (**d**) (two-sided Mann–Whitney *U-*test, *n* = 6 replicates, two cell lines, two independent experiments). **e**, S1P immunofluorescence quantification of FADU control (grey), dermal (yellow) and oral (blue) FB secretomes (two-sided Mann–Whitney *U-*test, *n* = 6 replicates, three independent FB secretomes). **f**, Immunofluorescence images of FADU + dermal (left) or oral (right) FB secretomes; blue, Hoechst; orange, phalloidin; green, S1P; scale bar, 20 µm (representative images from *n* = 6 biologically independent wells). **g**, Spheroid invasion of oSCC cells treated with oral FB secretomes (grey) with 5 µM S1PR1 inhibitor (S1PR1i, ponesimod, blue) or 10 µM S1PR3 inhibitor (S1PR3i, TY-52156, yellow; Kruskal–Wallis, Dunn’s multiple comparisons, *n* = 5 replicates). **h**, Spheroid invasion of oSCC treated with oral FB secretomes (grey) with 20 µM STAT3 inhibitor (STAT3i, STX-0119, blue), 10 µM NF-κB inhibitor (NF-κBi; JSH-23, yellow) or 2 µM AKT inhibitor (AKTi; MK-2206, red; Kruskal–Wallis, Dunn’s multiple comparisons, *n* = 5 replicates). **i**, Western blot of STAT3, phospho-STAT3^Tyr705^, B-actin in oSCC (FADU) + dermal or oral FB secretomes, ±PF543, S1P rescue, S1PR1 inhibitor (ponesimod) or STAT3 inhibitor (STX-0119); numbers represent normalized pSTAT3 to total STAT3 fold change relative to oral FB secretomes, run on separate blots with independent loading controls under identical conditions (western blots representative of two independent experiments). **j**, oSCC basal respiration in oSCC cells treated with oral FB secretomes ± lipid stripping (Cleanascite, blue, 24 h; OCR, oxygen consumption rate; *n* = 24 replicates in two cell lines treated with three FB secretomes, two independent experiments, two-sided Mann–Whitney *U-*test). **k**, Mitochondrial activity in oSCC cell lines treated with oral FB secretomes (blue) measured by membrane potential-dependent immunofluorescence (two-sided Mann–Whitney *U-*test, *n* = 8 replicates, two independent cell lines). **l**, Mitochondrial abundance in oSCC cell lines treated with oral FB secretomes (blue) measured by immunofluorescence (two-sided Mann–Whitney *U-*test, *n* = 8 replicates, two independent cell lines). **m**, Basal respiration in oSCC cells treated with combinations of oral FB secretomes, 20 µM imipramine, 2 µM S1P or 20 µM STAT3i (STX-0119; *n* = 5 independent replicates, two-sided Mann–Whitney *U-*test). **n**, Subcutaneous tumour growth rate in NSG mice injected with oSCC UMSCC01 (grey), UMSCC01 + dermal FBs (oSCC + dermal FB, yellow) or UMSCC01 + oral FBs (oSCC + oral FB, blue; two-sided Mann–Whitney *U-*test, *n* = 7 per group). **o**, Ki67 proliferation quantification in oSCC (grey), oSCC + dermal FB (yellow) and oSCC + oral FBs (blue) tumours (two-sided Mann–Whitney *U-*test, *n* = 14, two measurements per tumour). **p**, Kaplan–Meier survival curve of oSCC (black) and oSCC + oral FBs (blue; *n* = 7 per group, two-sided Mantel–Cox test). Box plots show minimum to maximum values (error bars), the box indicates 25th and 75th percentiles, and the line denotes the median. Source data

**Extended Data Fig. 2:**
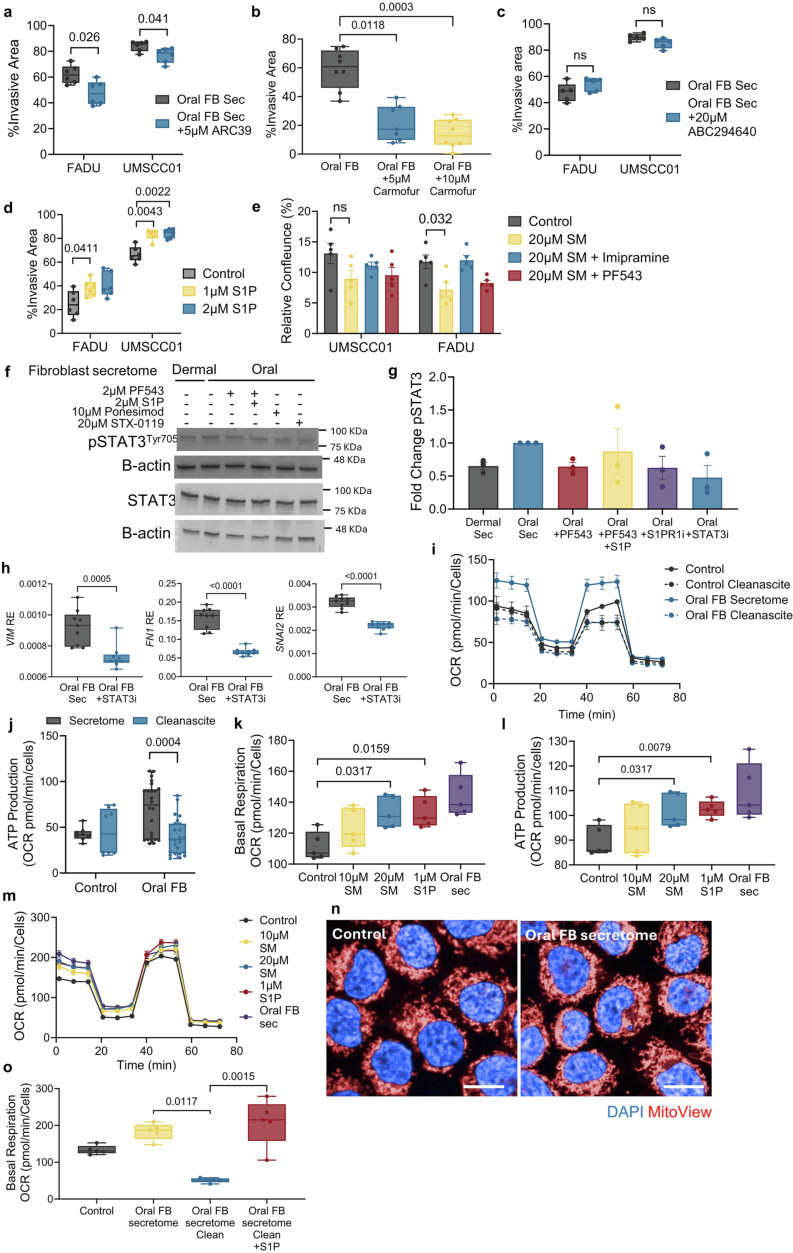
Sphingomyelin metabolism in oral SCC. **a**, oSCC spheroid invasion with oral fibroblast secretome treated with (blue) or without (grey) 5 µM ARC39 (two-sided Mann Whitney U, n = 6 replicates, two cell lines, two independent experiments). **b**, oSCC (FADU) spheroid invasion with oral fibroblast secretome (grey) with 5 µM (blue) and 10 µM (yellow) carmofur (Kruskal-Wallis, Dunn’s multiple comparisons, Oral FB, 10 µM: n = 8 replicates, 5 µM: n = 7). **c**, oSCC spheroid invasion +/- oral fibroblast secretomes control (grey), 20 µM SPHK2 inhibitor ABC294640 (blue) (two-sided Mann Whitney U, ns: non-significant, n = 5 replicates, two cell lines, two independent experiments). **d**, Spheroid invasion of oSCC with control media (grey) or sphingsine-1-phosphate (S1P, 1 µM yellow, 2 µM blue), (two-sided Mann Whitney U, n = 6 replicates, two cell lines, two independent experiments). **e**, Proliferation (relative confluence) of oSCC treated with 20 µM sphingomyelin (SM) and with 20 µM imipramine or 2 µM PF543 for 24 h (two-sided Mann Whitney U, n = 5 replicates, bars represent mean, error bars SEM). **f**, Replicate western blot of STAT3, phospho-STAT3Tyr705, B-actin in oral SCC (FADU) + dermal or oral fibroblast secretome, +/- PF-543, S1P rescue, S1PR1 inhibitor (ponesimod), or STAT3 inhibitor (STX-0119), run on separate blots with independent loading controls under identical conditions. **g**, Fold change in pSTAT3 levels relative to oral fibroblast secretome (Oral sec) in FADU western blots (n = 2 independent western blots). **h**, Relative expression (RE) of EMT genes VIM, FN1, and SNAI2 in oral SCC cells treated with oral fibroblast secretome (grey) and 20 µM STAT3 inhibitor (STAT3i, blue) STX-0119 (two-sided Mann Whitney U, n = 9 replicates across three independent samples). **i**, Oxygen consumption rate (OCR) plots of Seahorse assay of oSCC treated with fibroblast secretome ± Cleanascite (control: n = 8 biological replicates, oral FB: n = 24 replicates in two cell lines treated with three fibroblast secretomes). **j**, ATP production in oSCC cells treated with oral fibroblast secretomes +/- lipid stripping (Cleanascite: blue) (OCR: oxygen consumption rate, n = 24 replicates in two cell lines treated with three fibroblast secretomes, two independent experiments, two-sided Mann Whitney U). **k**, Basal respiration and ATP production (**l**) in oSCC (FADU) treated with sphingomyelin (SM), sphingosine-1-phosphate (S1P) or oral fibroblast secretome (24 h, two-sided Mann Whitney U, n = 5 replicates). **m**, Oxygen consumption rate (OCR) plots of Seahorse assay of FADU treated with sphingomyelin (SM), sphigosine-1-phosphate (S1P) or oral fibroblast secretome (n = 5 biological replicates). **n**, Representative immunofluorescence images of oSCC mitochondria stained with MitoView 633 (red), before and after exposure to oral fibroblast secretome, scale = 25 µm. **o**, Basal respiration of FADU cells treated with fibroblast secretome, Cleanascite treated secretome and rescue with addition of 2 µM S1P (Kruskal-Wallis, Dunn’s multiple comparisons, n = 5 replicates). Boxplots: minimum to maximum values (error bars), box: 25th and 75th percentiles; line: median. Bar plots display mean ± standard error of the mean. Source data

Lipidomic analysis showed oral fibroblasts secrete SMs, which are active signalling sphingolipids involved in key cell functions: cell death, proliferation and migration^28^. SMs can be metabolized to ceramide via sphingomyelinase enzymes and, subsequently, ceramides are converted to sphingosine and sphingosine-1-phosphate (S1P; Fig. 3a). S1P is an established driver of metastasis^29,30^, so we hypothesized that oral fibroblasts secrete SMs, which could drive oSCC invasion via S1P. To test this, we exposed oSCC spheroids to exogenous SMs, which confirmed SMs drive oSCC invasion (Fig. 3b). Conversely, inhibition of sphingomyelinase with imipramine significantly reduced SCC invasion after exogenous SMs and oral fibroblast secretome exposure (Fig. 3b,c). Similarly, the sphingomyelinase inhibitors ARC39 and ceramidase inhibitor (carmofur) also reduced invasion significantly (Extended Data Fig. 2a,b), further confirming the role of SM breakdown in invasion.

Next, we tested whether sphingosine kinase 1 and 2 (SPHK1 and SPHK2), which phosphorylate sphingosine to S1P^31^, mediate oSCC invasion after SM exposure. We found that SPHK1 inhibition decreased oSCC invasion significantly, while SPHK2 inhibition had no effect. Additionally, exogenous S1P increased oSCC invasion and rescued invasion after sphingomyelinase and SPHK1 inhibition (Fig. 3c,d and Extended Data Fig. 2c,d). To confirm that oral fibroblast secretomes increase intracellular S1P in oSCC, we performed S1P immunofluorescence staining, which validated that oral fibroblast secretome exposure increased intracellular S1P in oSCC, in contrast to dermal fibroblast secretome, which did not increase S1P (Fig. 3e,f). Additionally, inhibition of S1P receptor 1 (S1PR1) significantly decreased secretome-induced oSCC invasion (Fig. 3g). Given that invasion and proliferation are often inversely correlated, particularly during EMT, we verified that SM treatment did not significantly increase proliferation of oSCC (Extended Data Fig. 2e). Taken together, these data indicate that oral fibroblasts secrete SM, which induces invasion via the SM–ceramide–S1P pathway in oSCC.

Next, we examined the predicted upstream regulators of the genes that were highly expressed in SCC cells after oral fibroblast secretome exposure. This analysis predicted activation of SPHK1, S1P and its receptor S1PR3, as well as their downstream targets NF-κB, AKT and STAT3, in oral fibroblast-exposed oSCC. Importantly, S1P targets included EMT genes previously identified to be differentially expressed in SCC cell lines (Supplementary Table 5). To mechanistically test these observations, we quantified oSCC invasion following the pharmacological inhibition of STAT3, NF-κB or AKT. Inhibition of STAT3 or NF-κB, but not AKT, markedly suppressed oSCC invasive behaviour (Fig. 3h). Immunoblotting for phosphorylated STAT3 (pSTAT3) further confirmed activation of the SM–S1P–STAT3 axis in oSCC cells exposed to oral fibroblast secretomes. Inhibition of SPHK1 with PF543 reduced pSTAT3 in secretome-treated oSCC cells, and this suppression was rescued by exogenous S1P. Consistently, blockade of S1PR1 or STAT3 also lowered pSTAT3 levels (Fig. 3i and Extended Data Fig. 2f,g). Notably, STAT3 inhibition significantly attenuated the induction of canonical EMT-associated genes in secretome-treated oSCC cells (Extended Data Fig. 2h).

EMT, cell migration and invasion have high energy demands, so we next tested whether exposure to oral fibroblast secretomes increases energy production in oSCC. For this, we quantified oxidative phosphorylation (OXPHOS) in oSCC cells exposed to oral fibroblast secretomes, exogenous SM and S1P, which increased OXPHOS and ATP production (Fig. 3j and Extended Data Fig. 2i–m). The increase in respiration was due to increased mitochondrial activity and not increased abundance of mitochondria (Fig. 3k,l and Extended Data Fig. 2n). Importantly, exposure to lipid-stripped oral fibroblast secretomes decreased OXPHOS in oSCC cells profoundly, suggesting lipids taken up by oSCC cells impact OXPHOS. Moreover, both imipramine and STAT3 inhibitors prevented secretome-induced increases in OXPHOS, and the addition of S1P to lipid-stripped or imipramine-containing oral fibroblast secretomes restored OXPHOS induction (Fig. 3m and Extended Data Fig. 2o). Taken together, these data show that the oral fibroblast lipidome^32^, and specifically SM, increase oSCC respiration and invasion by activating the SM–ceramide–S1P–STAT3 signalling axis.

**Extended Data Fig. 3:**
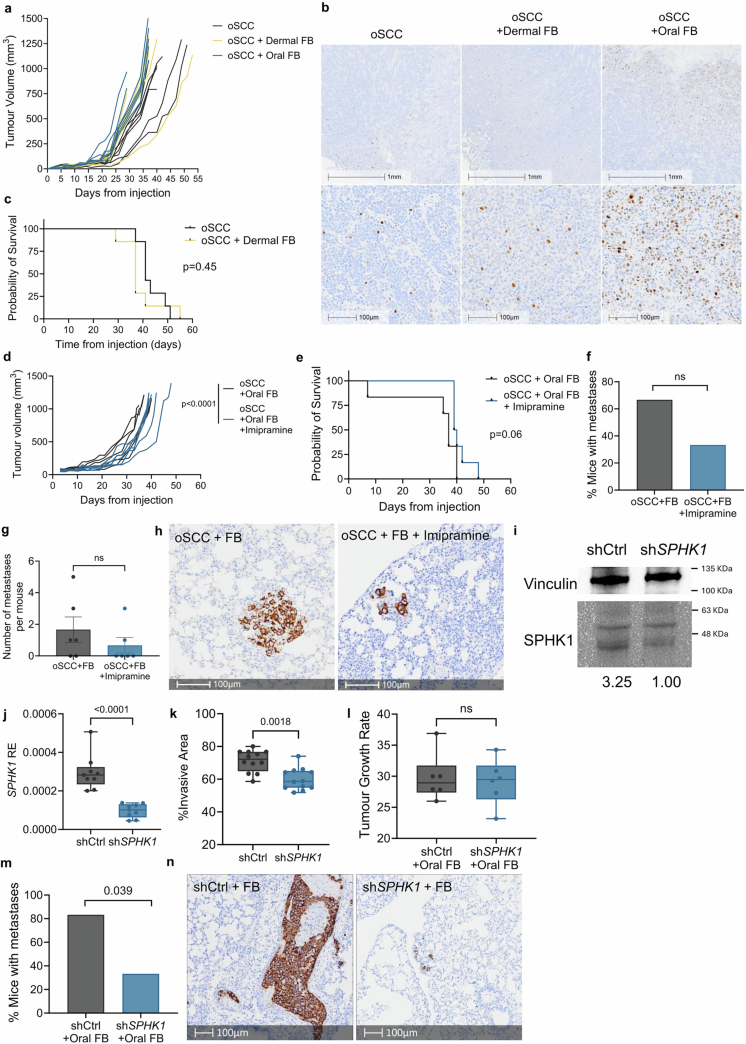
Targeting sphingomyelin metabolism in vivo. **a**, Tumour volumes of oSCC (black), oSCC + dermal fibroblasts (yellow), oSCC + oral fibroblast (blue) tumours in NSG mice (n = 7 mice per group). **b**, Ki67 immunohistochemistry staining (brown) in oSCC (left), oSCC + dermal fibroblasts (middle), oSCC + oral fibroblasts (right) tumours (top: scale=1 mm, bottom: representative image scale=100 µm). **c**, Kaplan-Meier survival curve of oral SCC (oSCC, black,) and oral SCC + dermal fibroblasts (oSCC + Dermal FB, yellow) mice (n = 7 per group, two-sided Mantel Cox test). **d**, Subcutaneous tumour volumes of oSCC + oral fibroblast tumours in NSG mice in control group (black) or mice treated with 40 mg/kg imipramine daily (blue) (n = 6 per group, two-sided linear mixed-effects model, p-values for treatment x time interaction shown). **e**, Kaplan-Meier survival curve of oral SCC + oral fibroblasts mice in control group (black) or mice treated with 40 mg/kg imipramine daily (blue) (n = 6 per group, two-sided Mantel Cox test). **f**, Bar plot representing the percent of mice that developed lung metastasis in subcutaneous oSCC + oral fibroblast model with and without imipramine treatment. **g**, Number of lung metastases per mouse in control and imipramine treated tumour bearing mice, quantified by human mitochondria immunohistochemistry, (two-sided Mann Whitney U, ns: non-significant, bars represent mean, error bars SEM, n = 6) **h**, Representative images of lung metastases in control and imipramine treated mice, stained for human mitochondria (brown), (representative image from n = 6 biologically independent mice per condition; all lungs were analysed, scale = 100 µm). **i**, SPHK1 protein expression in shCtrl and shSPHK1 oSCC cell line, numbers represent SPHK1 normalised to loading control (Vinculin), western blot from one biological experiment. **j**, Relative gene expression of SPHK1 in shCtrl and shSPHK1 oSCC cells quantified by qPCR (two-sided Mann Whitney U, n = 9). **k**, Spheroid invasion of shCtrl and shSPHK1 oSCC cell lines treated with oral fibroblast secretome (two-sided Mann Whitney U, n = 12 replicates, two independent secretomes). **l**, Subcutaneous tumour growth rate in NSG mice injected with shCtrl oral SCC + oral fibroblasts (shCtrl + oral FB, grey) or shSPHK1 oral SCC + oral fibroblasts (shSPHK1 + Oral FB, blue), (two-sided Mann Whitney U, n = 6 per group). **m**, Bar plot representing the percent of mice that developed lung metastasis in subcutaneous oSCC + oral fibroblast model with shCtrl and shSPHK1 oSCC cell lines (one-tailed Z-test, n = 6). **n**, Representative images of lung metastases in mice injected with shCtrl and shSPHK1 oSCC cells with oral fibroblasts, stained for human mitochondria (brown), (representative image from n = 6 biologically independent mice per condition; all lungs were analysed, scale = 100 µm). Boxplots: minimum to maximum values (error bars), box: 25th and 75th percentiles; line: median. Source data

To test whether fibroblasts promote oSCC progression and proliferation in vivo, we injected human oSCC cells alone or combined with either dermal or oral fibroblasts (oSCC tumours, oSCC + dermal FB tumours, oSCC + oral FB tumours) into the subcutaneous flanks of NSG mice. This revealed that oSCC + oral FB tumours had enhanced growth and proliferation compared to oSCC tumours, or oSCC + dermal FB tumours (Fig. 3n,o and Extended Data Fig. 3a,b). Critically, animals with oSCC + oral FB tumours had a shorter overall survival (Fig. 3p and Extended Data Fig. 3c). To validate the role of tumour SM metabolism in vivo, we treated mice injected with oSCC + oral FB tumours with imipramine, which significantly reduced tumour growth, and resulted in fewer lung metastases compared to untreated, tumour-bearing mice (Extended Data Fig. 3d–h). Additionally, we generated sh*SPHK1* oSCC cell lines, which expressed lower levels of SPHK1 and were less invasive than shCtrl oSCC lines (Extended Data Fig. 3i–k). To test the relevance of the pathway in vivo, we co-injected oral fibroblasts with sh*SPHK1* oSCC cells or shCtrl oSCC cells into the flanks of NSG mice, leading to oral FB + sh*SPHK1* oSCC and oral FB + shCtrl oSCC tumours. This revealed that oral FB + sh*SPHK1* oSCC tumours developed fewer lung metastases compared to animals with oral FB + shCtrl oSCC tumours, despite a similar rate of primary tumour growth (Extended Data Fig. 3l–n). Taken together, these data indicate that the SM–ceramide–S1P–STAT3 signalling axis, imposed by fibroblast lipid cues on oSCC, drives tumour invasion and metastasis.

## Oral fibroblast lipids impact oSCC progression and outcome in humans

**Fig. 4:**
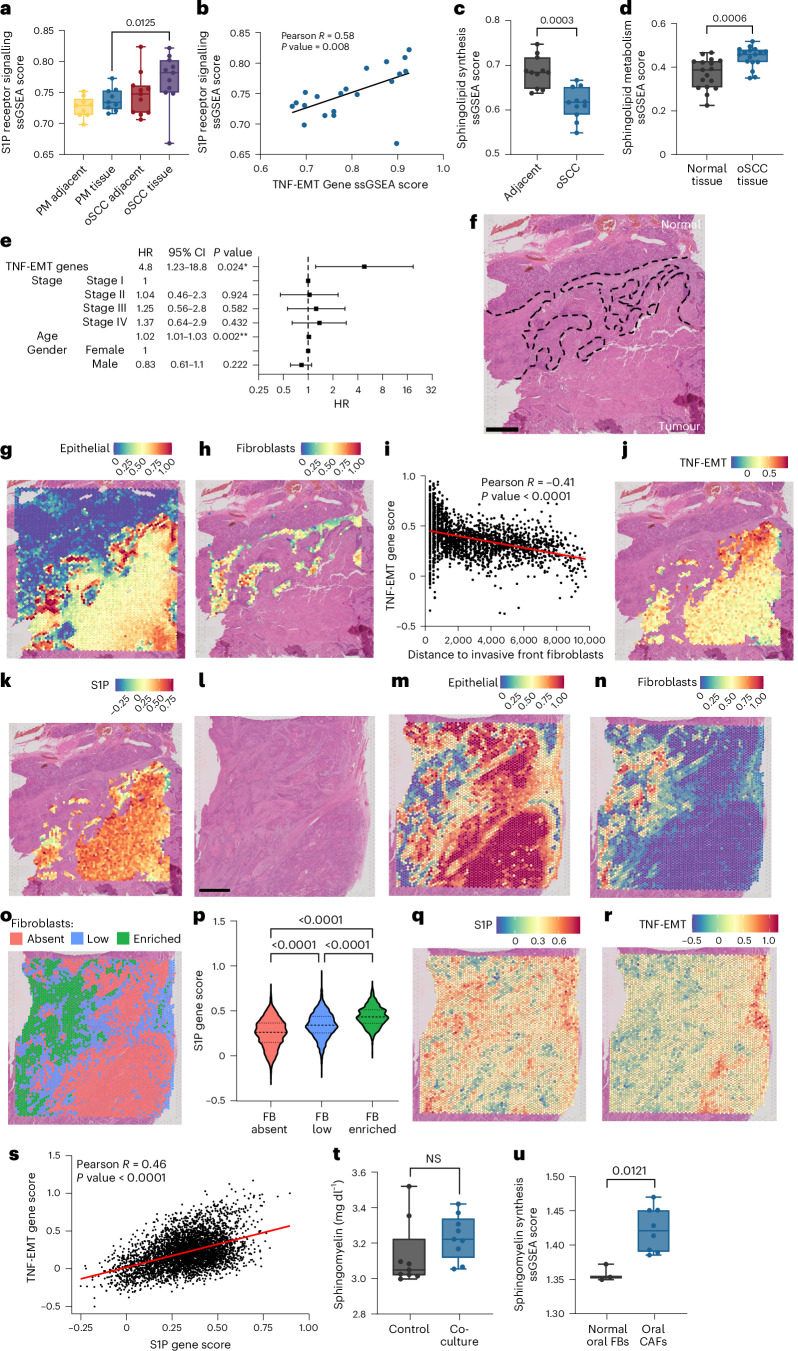
SM metabolism impacts oSCC progression and outcomes. **a**, Single-sample gene-set enrichment analysis (ssGSEA) of S1P regulated genes in oral premalignant tissue (PM, *n* = 9 biological replicates), oSCC (*n* = 11 biological replicates) and matched normal adjacent tissue (GSE202048, two-sided Mann–Whitney *U-*test) **b**, Two-sided Pearson correlation between S1P genes and TNF-EMT genes in PM tissue (*n* = 9 biological replicates) and oSCC (*n* = 11 biological replicates). **c**, ssGSEA of sphingolipid synthesis genes in oSCC and matched adjacent tissue (two-sided Mann–Whitney *U-*test, *n* = 11 biological replicates). **d**, ssGSEA of sphingolipid metabolism genes in oSCC and normal matched tissue (GSE186775, *n* = 17 biological replicates, two-sided Mann–Whitney *U-*test). **e**, Hazard ratio (HR; centre, square) and 95% confidence interval (CI; bars) of overall survival in two-sided multivariate Cox regression of TNF-EMT gene signature expression adjusted for stage, age and gender in the primary HNSCC cohort of the TCGA (*n* = 505 biological replicates; *P* values were derived from two-sided Wald tests). **f**, Human oSCC tumour H&E stain (*n* = 2 independent tumours analysed, one section per tumour; dashed lines represent invasive front stroma separating tumour, bottom, from normal tissue, top; scale bar, 1 mm). **g**,**h**, Corresponding spatial transcriptional expression of epithelial cell signatures (**g**) and spatial fibroblast signatures (**h**) at the invasive front of the oSCC tumour. Colour denotes the proportion of each spot represented by the signature (blue, low; red, high). **i**, Correlation between TNF-EMT signature score in the epithelial compartment and the distance to fibroblasts (two-sided Pearson correlation, *R* = −0.41, *P* < 0.0001; points denote individual spatial spots, *n* = 2,053). **j**, Spatial expression of TNF-EMT genes in the epithelial tumour compartment. Colour denotes the expression of the signature (blue, low; red, high). **k**, Spatial expression of S1P target genes in the epithelial compartment of the tumour. Colour denotes the expression of the signature (blue, low; red, high). **l**, Second human oSCC tumour H&E stain (*n* = 2 independent tumours analysed, one section per tumour; scale bar, 1 mm). **m**,**n**, Spatial transcriptional expression of epithelial cell signatures (**m**) and spatial fibroblast signatures (**n**). Colour denotes the proportion of each spot represented by the signature (blue, low; red, high). **o**, oSCC tumour region clustered by regions enriched for fibroblasts (green), with low fibroblasts (blue) or absent fibroblasts (red) by fibroblast signature expression level. **p**, Expression of S1P regulated genes in the oSCC tumour by fibroblast clusters (Kruskal–Wallis, Dunn’s multiple comparisons; FB absent: *n* = 2,205, FB low: *n* = 1,430, FB enriched: *n* = 900). **q**,**r**, Spatial expression of S1P regulated genes (**q**) and TNF-EMT signature in the second oSCC tumour (**r**). Blue, low; red, high. **s**, Correlation between S1P and TNF-EMT genes in the epithelial compartment of the oSCC tumour (two-sided Pearson correlation, *n* = 4,535). **t**, Quantification of SM in the secretomes of normal oral fibroblasts (control, grey) and oral fibroblasts co-cultured with oSCC cells (co-culture, blue; two-sided Mann–Whitney *U-*test, *n* = 9 biological replicates). **u**, ssGSEA score of SM synthesis genes in a dataset of normal oral fibroblasts (grey) and CAFs (blue) from individuals with oSCC (GSE135975; two-sided Mann–Whitney *U-*test, normal FB: *n* = 3 biological replicates, CAFs: *n* = 8 biological replicates). Box plots show the minimum to maximum values (error bars), the box shows the 25th and 75th percentiles, and the line indicates the median. Source data

**Extended Data Fig. 4:**
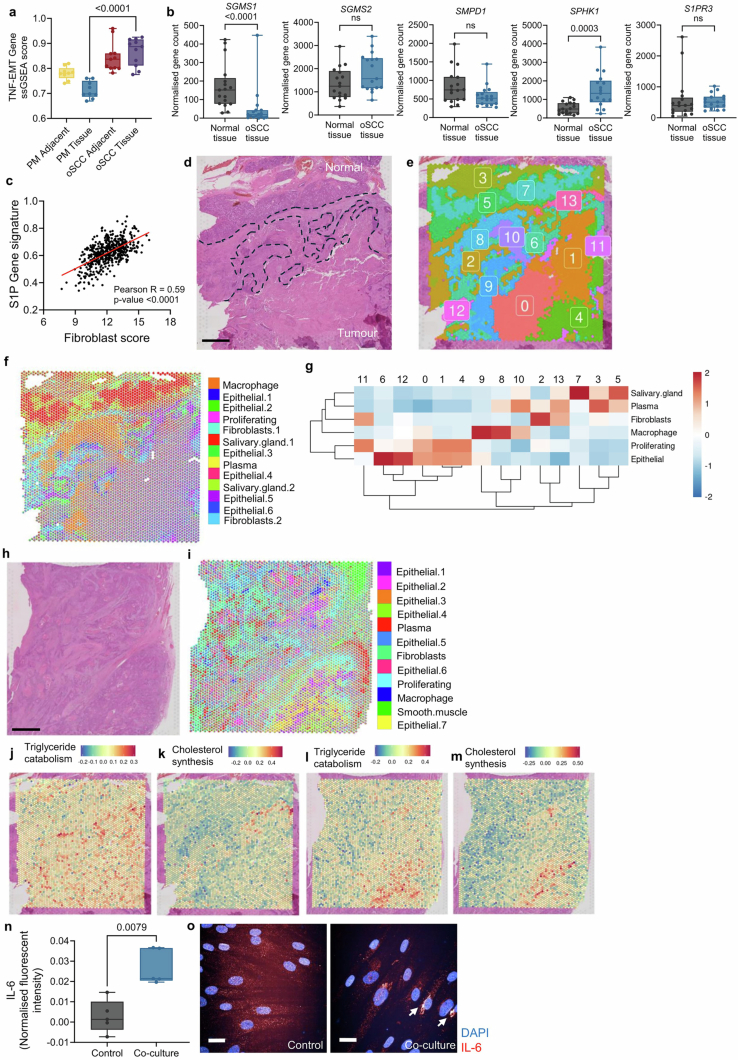
Sphingomyelin metabolism in oral SCC tumours. **a**, Single sample gene set enrichment analysis (ssGSEA) of differentially expressed TNF-EMT genes in oral premalignant tissue (PM, n = 9 biological replicates), oral SCC (oSCC, n = 11 biological replicates) and matched normal adjacent tissue (GSE202048, two-sided Mann Whitney U). **b**, Expression of a panel of sphingomyelin metabolism genes in oSCC and normal matched tissue (GSE186775, n = 17 biological replicates, two-sided Mann Whitney U, ns: non-significant). **c**, Correlation between S1P genes and Fibroblast signature in TCGA HNSCC primary tumour cohort (two-sided Pearson correlation, n = 520 biological replicates). **d**, Human oSCC tumour haematoxylin and eosin stain (dotted lines represent invasive front stroma separating tumour, bottom, from normal tissue, top, n = 2 independent tumours analysed; one section per tumour). **e**, The corresponding, colour map to **d** shows gene expression-based clusters representing distinct spatial transcriptional signatures. **f**, Spatial distribution of deconvoluted cell type signatures, each spot represents pie chart displaying proportion of each signature. **g**, Heatmap showing overlap between clusters and cell type signatures in oSCC spatial transcriptomics. **h**, Haematoxylin and eosin stain of second human oSCC tumour (n = 2 independent tumours analysed; one section per tumour) with corresponding **i**, spatial distribution of deconvoluted cell type signatures, each spot represents pie chart displaying proportion of each signature. **j**, Spatial expression of triglyceride catabolism and cholesterol synthesis (**k**) gene signatures in first oral SCC tumour, colour: gene expression (low: blue, red: high). **l**, Spatial expression of triglyceride catabolism and cholesterol synthesis (**m**) gene signatures in second oral SCC tumour, colour: gene expression (low: blue, red: high). **n**, Quantification of IL-6 expression by immunofluorescence in oral fibroblasts co-cultured with oral SCC cells (two-sided Mann Whitney U, n = 5 biological replicates). **o**, Representative immunofluorescent images of IL-6 (red) in control and co-cultured oral fibroblasts (representative image from n = 5 biologically independent wells; 122 fields per well analysed, scale: 25 µm, arrows highlight IL-6 expression). Boxplots: minimum to maximum values (error bars), box: 25th and 75th percentiles; line: median. Source data

We next hypothesized that resident tissue-specific fibroblasts impact early-stage in situ SCC progression to invasive SCC in humans and tested whether SM signalling impacts early-stage human oSCC. For this, we compared the expression of sphingolipid metabolic pathways in oral premalignant tissue and oSCC, and their respective adjacent tissues^33^. This comparison showed that S1P (Fig. 4a) and TNF/EMT (Extended Data Fig. 4a) signalling genes, which we found upregulated by oral fibroblast secretome exposure in oSCC cells, are significantly correlated (Fig. 4b); crucially, their expression increases with progression from premalignant stages to established oSCC. Furthermore, sphingolipid synthesis genes were also expressed significantly higher in oSCC tumour-adjacent tissue (Fig. 4c), supporting a role for tumour-adjacent stromal SM synthesis and provision. The upregulation of sphingolipid metabolism and key genes in the pathway was further validated in a second, independent cohort of oSCC (Fig. 4d and Extended Data Fig. 4b). Finally, The Cancer Genome Atlas (TCGA) Head and Neck Squamous Cell Carcinoma (HNSCC) primary tumour cohort confirmed that the TNF/EMT gene signature in oSCC cells is prognostic of poor overall survival (Fig. 4e), and S1P signalling significantly correlated with fibroblast signatures (Extended Data Fig. 4c).

Subsequently, we explored the spatial relationship between oral fibroblasts and oSCC tumour cells. For this, we performed spatial transcriptomics (Visium 10x Genomics) of an oSCC tumour (Fig. 4f). Deconvolution analysis revealed distinct tissue biology clusters including fibroblasts, salivary glands, macrophages and epithelial cells (Extended Data Fig. 4d–g and Supplementary Table 6). We compared the transcriptional programmes at the interface between the invasive leading edge of the oSCC (Fig. 4g) and the tumour-adjacent fibroblasts (Fig. 4h), which confirmed that tumour cells in physical proximity to fibroblasts at the invasive edge of the tumour had a higher expression of TNF/EMT genes. In contrast, tumour cells that were at greater distances from fibroblasts expressed lower levels of TNF/EMT genes; furthermore, gene expression of the S1P–STAT3 signalling pathway followed a similar gradient, strongest at the invasive edge near fibroblasts, and decreasing with distance (Fig. 4i–k). Spatial gene expression patterns were validated in a second oSCC tumour (Fig. 4l and Extended Data Fig. 4h,i), which confirmed that S1P gene expression and TNF-EMT programmes were significantly higher in fibroblast-rich tumour areas (Fig. 4m–s). Importantly, other lipid metabolic pathways linked to malignant progression in other SCCs, such as TG metabolism, were not the principal lipid metabolic pathways expressed in oSCC (Extended Data Fig. 4j–m).

**Extended Data Fig. 5:**
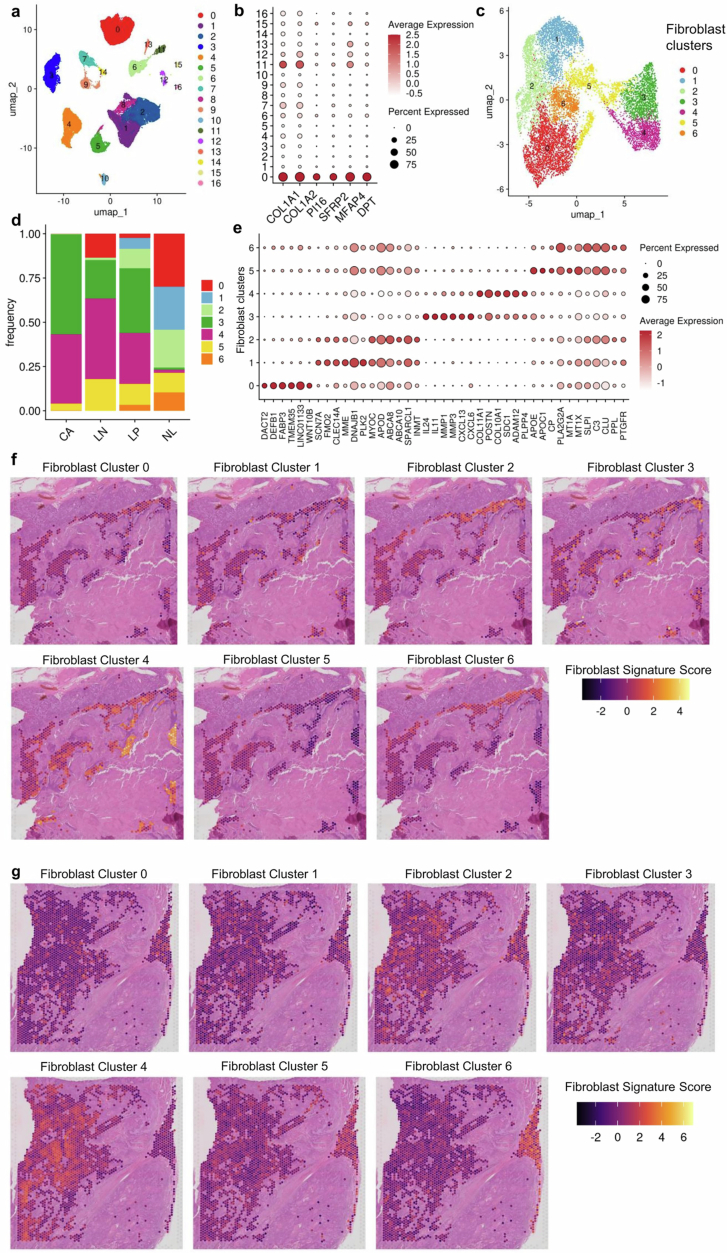
Fibroblast heterogeneity in oral SCC. **a**, UMAP plot of HNSCC single cell RNA sequencing dataset (GSE181919) containing HNSCC primary and metastatic tumours, premalignancy (leukoplakia) and normal oral tissue. **b**, Dot plot of fibroblast marker genes showing enrichment of fibroblasts in cluster 0. **c**, UMAP plot of fibroblasts (cluster 0) subclustered into distinct fibroblast populations. **d**, Bar plot displaying the frequency of each fibroblast population in HNSCC primary tumours (CA), lymph node metastases (LN), premalignancy (LP) and normal oral tissue (NL). **e**, Dot plot of the expression of top six marker genes for each fibroblast cluster, size of dot represents percent of cells gene expressed in, colour represents expression level (white: low, red: high). **f**, Expression of each fibroblast clusters marker gene signature in spatial transcriptomics oSCC tumour fibroblast regions (colour represents expression levels, black: low, yellow: high). **g**, Expression of each fibroblast clusters marker gene signature in second spatial transcriptomics oSCC tumour fibroblast regions (colour represents expression levels, black: low, yellow: high).

Cancer-associated fibroblasts (CAFs) maintain lipid homeostasis in the pancreatic tumour microenvironment^34,35^, so we next examined whether the lipid signatures in tissue-resident fibroblasts are maintained with CAF activation. We first co-cultured oSCC cells with adult oral fibroblasts, which led to oral fibroblast activation, validated via upregulation of the CAF marker interleukin-6 (Extended Data Fig. 4n,o) and a trend towards increased SM secretion (Fig. 4t). Additionally, we found SM biosynthesis expression was upregulated in CAFs from oSCC human tumours compared to normal oral fibroblasts (Fig. 4u). Next, we explored fibroblast heterogeneity in our oSCC spatial transcriptomic tumours. For this, we extracted fibroblast single-cell RNA-sequencing data from HNSCC tumours, metastases, premalignant oral lesions (leukoplakia) and normal oral tissue (Extended Data Fig. 5a–d), and we then determined the top marker genes representing each fibroblast subpopulation (Extended Data Fig. 5e and Supplementary Table 7). Fibroblast-subtype signatures were then projected onto our spatial samples (Extended Data Fig. 5f,g), which revealed fibroblasts in human oSCC are highly heterogenous, with prominent representation of both normal (cluster 2) and CAF (cluster 4) populations within tumours and within invasion-rich tumour areas. Notably, the CAF population also localized to tumour areas with the highest TNF-EMT and SM gene signatures, indicating CAFs may further contribute to lipid-mediated invasion. Thus, these data show in human oSCC that tissue-resident fibroblasts and CAFs are spatially associated with lipid signalling pathways linked to oSCC invasion.

## Lung fibroblasts transfer TGs to drive luSCC invasion

**Fig. 5:**
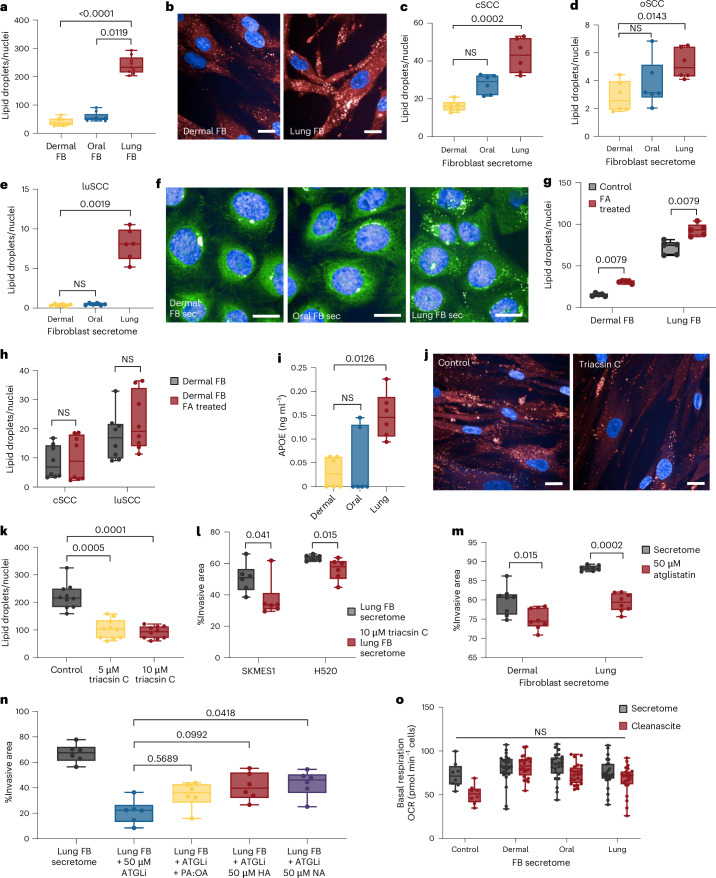
Lung fibroblasts transfer TGs to drive aggressive luSCC. **a**,**b**, Fibroblast lipid droplet quantification (**a**) (Kruskal–Wallis, Dunn’s multiple comparisons, *n* = 9, three replicates, three cell lines per group) and immunofluorescence (**b**): red, LipidSpot, blue, Hoechst. Scale bars, 25 µm. **c**–**e**, Quantification of fibroblast BODIPY-labelled lipids transferred to SCC cells through secretomes (IC19) (**c**), FADU (**d**) and SKMES1 (**e**) (Kruskal–Wallis, Dunn’s multiple comparisons, *n* = 6 replicates, two fibroblast secretomes). **f**, Immunofluorescence images from cSCC cell line IC19 treated with BODIPY-labelled dermal fibroblast (left), oral fibroblast (middle) and lung fibroblast (right) secretomes. Green, BODIPY; blue, Hoechst. Scale bar, 25 µm. **g**, Lipid droplets in dermal and lung fibroblasts after 50 µM oleic acid and 100 µM palmitic acid supplementation (two-sided Mann–Whitney *U-*test, *n* = 5 replicates per cell line). **h**, BODIPY lipids transferred from dermal fibroblasts (grey) and fatty acid (FA)-supplemented dermal fibroblasts (red) to cSCC and luSCC (two-sided Mann–Whitney *U-*test, *n* = 8 replicates across two cell lines per group). **i**, APOE in dermal (yellow), oral (blue) and lung (red) fibroblast secretomes (Kruskal–Wallis, Dunn’s multiple comparisons, *n* = 6, two measurements, three fibroblast secretomes). **j**, Immunofluorescence of lipid droplets in control lung fibroblasts (left) and after treatment with 10 µM long-chain acyl-CoA synthetase (ACSL) inhibitor triacsin C (right). Red, LipidSpot; blue, Hoechst. Scale bars, 25 µm. **k**, Quantification of lipid droplets in lung fibroblasts after treatment with 5 µM and 10 µM triacsin C (Kruskal–Wallis, Dunn’s multiple comparisons, *n* = 10 biological replicates). **l**, Spheroid invasion of luSCC exposed to secretomes of lung fibroblasts treated with 10 µM triacsin C (two-sided Mann–Whitney *U-*test, *n* = 6 replicates per cell line). **m**, luSCC (SKMES1) spheroid invasion treated with 50 µM atglistatin (red) and vehicle control (grey) and dermal or lung fibroblast secretomes (two-sided Mann–Whitney *U-*test, *n* = 8 replicate spheroids treated with two independent secretomes per group). **n**, luSCC spheroid invasion treated with lung fibroblast secretomes, with the secretomes containing 50 µM atglistatin (blue, ATGLi) and rescued with addition of fatty acids (50 µM oleic acid and 100 µM palmitic acid, PA:OA, yellow; 50 µM heneicosanoic acid (HA), red; 50 µM nervonic acid (NA), purple; Kruskal–Wallis, Dunn’s multiple comparisons, *n* = 6 replicates). **o**, Basal respiration of luSCC treated with fibroblast secretomes and with and without lipid stripping by Cleanascite (red; *n* = 24 replicates, two cell lines, three fibroblast secretomes, two experiments, Kruskal–Wallis). Box plots show minimum to maximum values (error bars), the box indicates the 25th and 75th percentiles, and the line denotes the median. Source data

**Extended Data Fig. 6:**
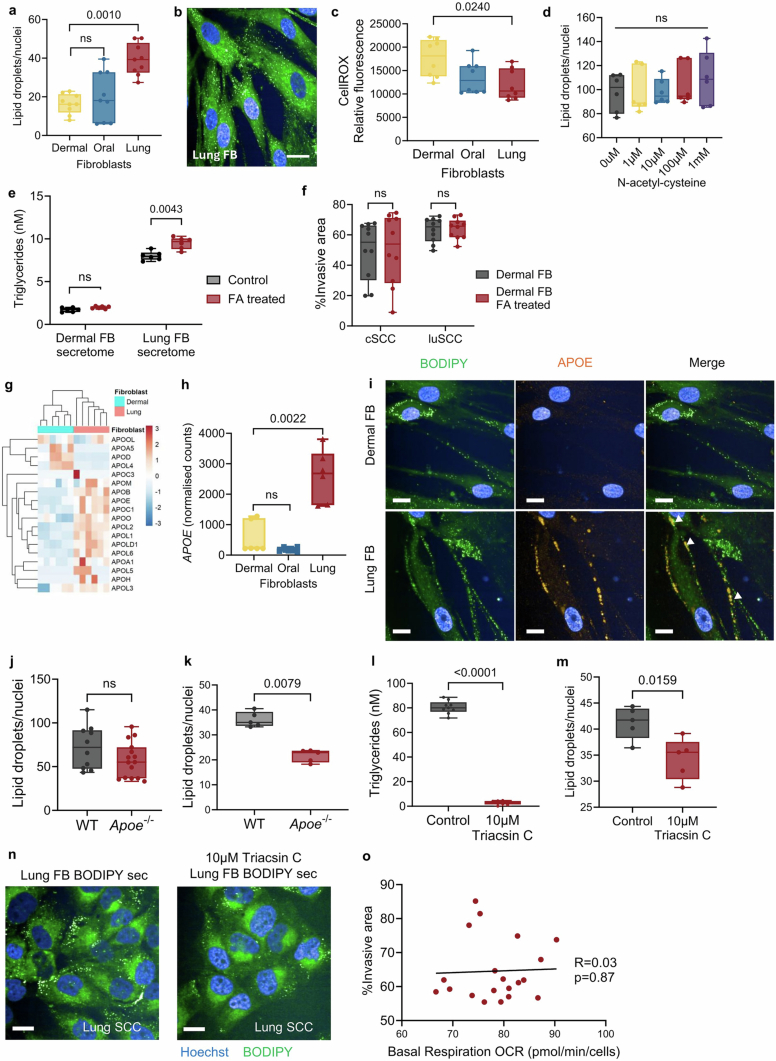
Lung fibroblast triglyceride biology. **a**, Quantification of BODIPY stained fibroblast lipid droplets (Kruskal-Wallis, Dunn’s multiple comparisons, ns: non-significant, n = 9, three replicates, three cell lines/group). **b**, Immunofluorescence: lipid droplets in lung fibroblasts, green: BODIPY, blue: Hoechst, scale=20 µm. **c**, Reactive oxygen species quantified in fibroblasts over 24 h with CellROX (Kruskal-Wallis, Dunn’s multiple comparisons, n = 8, four independent replicates of two cell lines per group). **d**, Lipid droplets in lung fibroblasts + N-acetyl-cysteine (Kruskal-Wallis test, ns: non-significant, n = 6 replicates, two cell lines). **e**, Triglyceride quantification in secretome of control fibroblasts (grey) or fibroblasts treated with fatty acid (FA) supplemented media (red) (two-sided Mann Whitney U, n = 6 biological replicates across two independent secretomes). **f**, cSCC and luSCC spheroid invasion after dermal fibroblast secretome (Dermal FB), or after fatty acid (FA) supplemented media cultured dermal fibroblasts (two-sided Mann Whitney U, ns: non-significant, n = 10 replicates, two cell lines, two experiments). **g**, Apolipoprotein genes expression in dermal, lung fibroblasts, colour: red high, blue low. **h**, APOE gene expression in RNA sequencing of dermal, oral, lung fibroblasts (two-sided Mann Whitney U, ns: non-significant, n = 6 replicates, three cell lines). **i**, Immunofluorescence: lipid droplets (left, BODIPY), APOE (middle), merged (right), in dermal (top) and lung fibroblasts (bottom). Arrow: co-localisation APOE and lipid droplets, green: BODIPY, orange: APOE, blue: Hoechst, scale =20 µm. **j**, Lipid droplets quantified in wild type (WT) and Apoe knockout (Apoe-/-) murine lung fibroblasts (two-sided Mann Whitney U, WT n = 10 biological replicates, Apoe-/- n = 15 biological replicates). **k**, Quantification of lipid droplets transferred from WT and Apoe-/- lung fibroblast to lung SCC cells with BODIPY labelled secretome (two-sided Mann Whitney U, n = 5 biological replicates). **l**, Triglycerides in lung fibroblast secretomes after treatment with 10 µM Triacsin C for 48 h (two-sided Mann Whitney U, n = 9 biological replicates across three independent secretomes). **m**, Quantification of lipid droplets transferred from control (grey) or 10 µM Triacsin C treated (red) lung fibroblasts to lung SCC cells (two-sided Mann Whitney U, n = 5 biological replicates). **n**, Representative images of lung SCC cells treated with BODIPY labelled lung fibroblast secretomes (left: control fibroblast secretome, right: Triacsin C treated fibroblast secretome, representative image from n = 5 biologically independent wells; 122 fields per well analysed, scale: 25 µm). **o**, Correlation between luSCC spheroid + fibroblast secretome invasion and oxidative phosphorylation (two-tailed Pearson, R = 0.037, p = 0.8749). Boxplots: minimum to maximum values (error bars), box: 25th and 75th percentiles; line: median. Source data

Lung fibroblasts produce and secrete TGs and strongly promote SCC invasion (Figs. 1 and 2f). To study whether TGs drive luSCC invasion, we first measured TG content in resident fibroblasts with two fluorescent dyes that bind neutral lipids (BODIPY and LipidSpot). Quantification of lipid droplets, the organelles that store TGs, confirmed that lung fibroblasts contained significantly more lipid droplets than fibroblasts from other sites (Fig. 5a,b and Extended Data Fig. 6a,b). To rule out the possibility that lipid droplets in cultured fibroblasts arise due to cellular stress, we confirmed that lung fibroblasts do not display higher levels of reactive oxygen species (ROS) compared to other fibroblasts. Moreover, treatment of lung fibroblasts with the antioxidant *N*-acetyl-L-cysteine in vitro did not alter the lipid droplet content (Extended Data Fig. 6c,d). We then examined the effect of BODIPY-stained fibroblast secretomes on SCC and found that TG-rich lung fibroblasts transfer significantly more neutral lipids to SCC cells than TG-poor oral and dermal fibroblasts (Fig. 5c–f).

Mechanistically, we explored whether raising TG content in dermal fibroblasts could drive SCC invasion. For this, we cultured dermal fibroblasts, which are naturally low in TGs (Fig. 2f), in fatty acid-enriched media (50 µM oleic acid, 100 µM palmitic acid) to increase the synthesis of lipid droplets. This culture media significantly increased lipid droplets in dermal and lung fibroblasts (Fig. 5g). However, higher lipid droplet content in dermal fibroblasts did not lead to increased secretion of TGs (Extended Data Fig. 6e), nor greater lipid transfer to SCC cells (Fig. 5h), nor greater SCC invasion (Extended Data Fig. 6f), showing that fibroblast lipid production alone does not enhance lipid transfer and SCC invasion. To explore this further, we compared the expression of lipid transport genes and found that lipid transport genes are differentially expressed in dermal, oral and lung fibroblasts. Specifically, lung fibroblasts highly express apolipoprotein genes (Extended Data Fig. 6g,h) compared to dermal fibroblasts, which could explain why a greater TG content in dermal fibroblasts does not lead to greater lipid transfer to SCC and invasion. To test whether apolipoproteins, such as apolipoprotein E (APOE), transport TGs from lung fibroblasts to the extracellular space, we performed immunofluorescence in lung fibroblasts to show APOE was expressed and colocalized with lipid droplets (Extended Data Fig. 6i) and found lung fibroblast secretomes contained significantly higher levels of secreted APOE (Fig. 5i). To validate fibroblast APOE transports TGs to SCC, we extracted lung fibroblasts from wild-type and *Apoe*^−*/*−^ mice and compared TG content and transfer. We found that while wild-type and *Apoe*^−*/*−^ fibroblasts had a similar TG content, *Apoe*^−*/*−^ fibroblasts transferred significantly fewer lipids to luSCC cells, in keeping with the lower ability to transport lipids to the extracellular space (Extended Data Fig. 6j,k). These data support that fibroblast TGs require transport proteins to mediate their transfer to SCC cells, and transport proteins are expressed in lung fibroblasts, but not dermal fibroblasts, fuelling luSCC invasion.

To test the role of lung fibroblast TG synthesis in luSCC invasion, we treated fibroblasts with triacsin C, a long-chain fatty acid acyl-CoA synthetase inhibitor that inhibits the formation of fatty acyl-CoA, the first step in the TG synthesis pathway. Triacsin C significantly reduced the number of lipid droplets in lung fibroblasts (Fig. 5j,k) and the amount of TGs in their secretomes (Extended Data Fig. 6l). This resulted in significantly less transfer of TGs to luSCC (Extended Data Fig. 6m,n) and significantly reduced luSCC invasion (Fig. 5l). Taken together, these data show lung fibroblasts produce more TGs and lipid droplets than dermal and oral fibroblasts, and transfer TGs to luSCC to drive invasion.

To study how TGs taken up by luSCC cells promote invasion, we hypothesized that lung cancer cells could break down TGs to FAs for energy use, using adipose triglyceride lipase (ATGL), generating metabolites in the process that could activate oncogenic pathways^36^. Therefore, we first tested if lung fibroblast-induced luSCC invasion requires the breakdown of TGs. For this, we inhibited ATGL in luSCC cells with atglistatin and found inhibition of ATGL significantly reduced luSCC invasion after lung fibroblast exposure, which was partially recovered with addition of exogenous fatty acids (Fig. 5m,n). Fatty acids derived from TGs can be used to increase OXPHOS^37^, so we next examined the impact of fibroblast secretomes on luSCC respiration. Intriguingly, fibroblast secretomes had no impact on OXPHOS, and luSCC OXPHOS was not correlated with luSCC invasion. Furthermore, the removal of lipids from fibroblast secretomes did not impact respiration (Fig. 5o and Extended Data Fig. 6o), indicating that fibroblast lipids in luSCC are not used for OXPHOS.

**Fig. 6:**
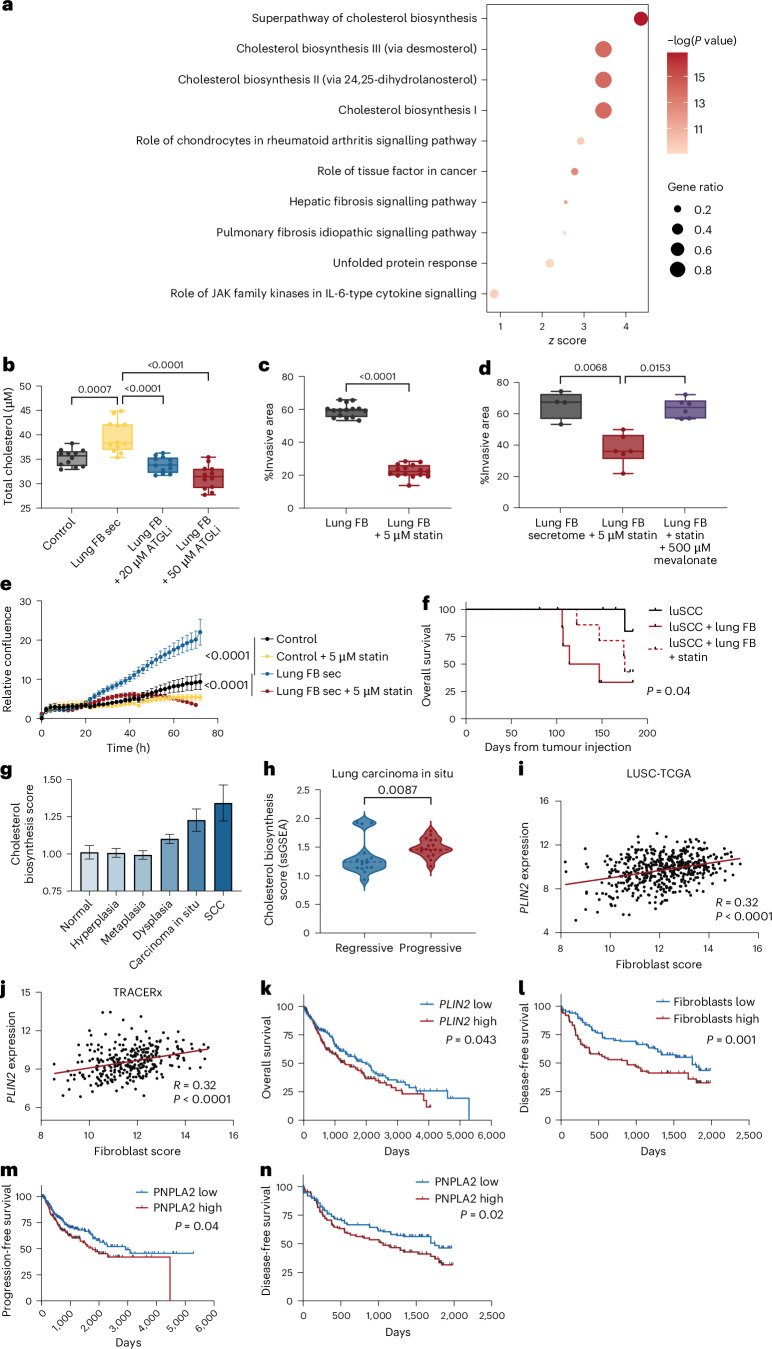
Lung fibroblast TGs fuel cholesterol synthesis in luSCC. **a**, Pathways enriched for genes differentially expressed in luSCC cell line SKMES1 after treatment with lung fibroblast secretomes. Colour represents significance of enrichment (right-tailed Fisher’s exact test). *z*-score represents predicted pathway acitvation (positive indicates activated, negative indicates inhibited). Dot size represents gene ratio (proportion of pathway genes present in the dataset). **b**, Cholesterol in luSCC cell lines treated with control media, lung fibroblast secretomes (lung FB sec) and lung fibroblast secretomes with ATGLi (two-sided Mann–Whitney *U-*test, *n* = 24 independent measurements of two cell lines treated with three independent fibroblast secretome). **c**, Spheroid invasion of luSCC (H520) treated with lung fibroblast secretomes with 5 µM statin (atorvastatin) or vehicle control (two-sided Mann–Whitney *U-*test, *n* = 15 replicate spheroids treated with three independent secretomes per group). **d**, Spheroid invasion of luSCC (SKMES1) treated with lung fibroblast secretomes, adding 5 µM statin (red) to the secretomes and rescued with 500 µM mevalonate (purple; Kruskal–Wallis, Dunn’s multiple comparisons, control: *n* = 4 replicate spheroids, statin: *n* = 6 replicate spheroids). **e**, LuSCC cell proliferation in control DMEM, lung fibroblast secretomes with statin (atorvastatin) or vehicle control (mixed-effect general linear model, data represent the mean and standard error, control *n* = 8 independent measurements of two cell lines, lung fibroblast *n* = 24 independent measurements of two cell lines treated with three independent fibroblast secretomes). **f**, Kaplan–Meier survival curve of NSG mice subcutaneously injected with luSCC cell line SKMES1 alone (solid black line, *n* = 7), co-injected with SKMES1 and lung fibroblasts (luSCC + lung FB, solid red line, *n* = 7) and co-injected with SKMES1 and lung fibroblasts and treated with atorvastatin (20 mg per kg body weight; luSCC + Lung FB + statin, broken red line, *n* = 8; two-sided Mantel–Cox test, luSCC + Lung FB versus luSCC + Lung FB + Statin: *P* = 0.04). **g**, Average expression of genes in cholesterol synthesis pathway in lung bronchial biopsy samples across lung cancer stages (GSE33479, normal *n* = 27 biological replicates, hyperplasia *n* = 15 biological replicates, metaplasia *n* = 15 biological replicates, dysplasia *n* = 38 biological replicates, carcinoma in situ *n* = 13 biological replicates, SCC *n* = 14 biological replicates). **h**, ssGSEA score of cholesterol synthesis pathway in lung carcinoma in situ samples that regressed (blue) or progressed (red) to invasive luSCC (GSE94611, two-sided Mann–Whitney *U-*test, regressive *n* = 16, progressive *n* = 17). **i**, Correlation between *PLIN2* gene expression (log_2_RSEM) and fibroblast score in TCGA Lung Squamous Cell Carcinoma (LUSC) cohort (two-sided Pearson correlation, *R* = 0.32, *P* < 0.0001, *n* = 501). **j**, Correlation between *PLIN2* gene expression (normalized counts) and fibroblast score in the TRACERx luSCC cohort (two-sided Pearson correlation, *R* = 0.32, *P* < 0.0001, *n* = 295). **k**, Kaplan–Meier curve of overall survival in the LUSC TCGA cohort based on expression of *PLIN2* relative to the median expression (two-sided log-rank test, *P* = 0.043, *n* = 501). **l**, Kaplan–Meier curve of disease-free survival in the TRACERx luSCC cohort based on fibroblast signature score relative to the median (two-sided log-rank test, *P* = 0.001, *n* = 295). **m**, Kaplan–Meier curve of progression-free survival in the LUSC TCGA cohort based on expression of *PNPLA2* (ATGL) relative to the median expression (two-sided log-rank test, *P* = 0.04, *n* = 501). **n**, Kaplan–Meier of disease-free survival in TRACERx luSCC cohort based on the expression of *PNPLA2* (ATGL) relative to the median expression (two-sided log-rank test, *P* = 0.02, *n* = 295). The box plots show the minimum to maximum values (error bars), boxes indicate the 25th and 75th percentiles, and the line indicates the median. Bar plots show the mean ± s.e.m. Data points in the line graphs represent the mean ± s.e.m. (error bars). Source data

**Extended Data Fig. 7:**
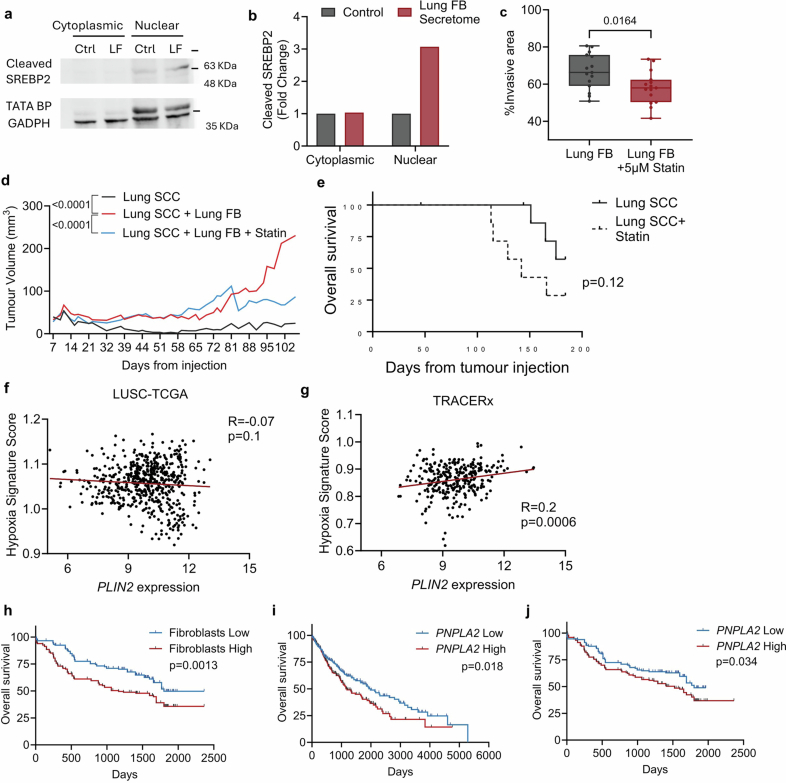
Lipid droplet biology accelerates luSCC progression. **a**, Western blot and **b**, quantification of cytoplasmic and nuclear cleaved SREPB2 in luSCC cell line SKMES1 after treatment with lung fibroblast secretome (red, western blot from one biological experiment). **c**, LuSCC (SKMES1) spheroid invasion + lung fibroblast secretome +/- 5 µM atorvastatin (two-sided Mann Whitney U, n = 15 replicates, 3 lung fibroblast secretomes, boxplots: minimum to maximum values (error bars), box: 25th and 75th percentiles; line: median). **d**, Mean tumour volume luSCC (n = 8), luSCC + lung fibroblasts (n = 7), luSCC + lung fibroblasts + atorvastatin (n = 8). (two-sided linear mixed-effects model, p- values for treatment × time interaction shown) **e**, Kaplan-Meier of survival of NSG mice subcutaneously injected with lung SCC cell line SKMES1 treated with atorvastatin (20 mg/kg) (broken line, n = 8) (two-sided Mantel Cox test). **f**, Correlation between hypoxia gene expression signature and fibroblast signature in the LUSC TCGA dataset (two-sided Pearson correlation, n = 553) **g**, and the TRACERx cohort (two-sided Pearson correlation, n = 290). **h**, Kaplan-Meier survival in TRACERx luSCC by fibroblast signature relative to the median (two-sided log-rank test, p = 0.0013, n = 295). **i**, Kaplan-Meier survival in LUSC TCGA by PNPLA2 (ATGL) relative expression to the median (two-sided log-rank test, p = 0.0018, n = 501). **j**, Kaplan-Meier survival in TRACERx luSCC by PNPLA2 (ATGL) relative expression to the median (two-sided log-rank test, p = 0.034, n = 295). Source data

To investigate how TGs promote luSCC invasion, we examined pathway-level transcriptional changes in luSCC cells exposed to lung fibroblast secretomes. Remarkably, cholesterol biosynthesis emerged as the most significantly upregulated pathway (Fig. 6a and Supplementary Tables 8 and 9), consistent with prior reports implicating cholesterol synthesis in tumour invasion^38–40^. To confirm activation of the cholesterol production pathway, we assessed activation of SREBP2, the master transcriptional regulator of this pathway. Lung fibroblast secretome exposure increased total cellular cholesterol (Fig. 6b) and induced SREBP2 activation and nuclear translocation in luSCC cells (Extended Data Fig. 7a,b). Because cholesterol synthesis depends on acetyl-CoA, which can be supplied by fatty acid metabolism downstream of TG hydrolysis^41^, we tested whether blocking TG breakdown would affect this pathway. Consistent with this model, ATGL inhibition prevented the increase in cholesterol production observed after lung fibroblast exposure (Fig. 6b). These data show cholesterol synthesis in luSCC cells requires the breakdown of TGs that are transferred from lung fibroblasts.

To further assess the link between invasion and cholesterol synthesis in luSCC, we treated luSCC cells with atorvastatin, a cholesterol synthesis inhibitor, which significantly decreased lung fibroblast-induced invasion and proliferation in luSCC cells. Critically luSCC invasion could be restored by the addition of mevalonate, the intermediate metabolite in the cholesterol synthesis pathway, which sits downstream of the key rate-limiting enzyme HMG-CoA reductase, the target of statins (Fig. 6c–e and Extended Data Fig. 7c). These data show that lung fibroblasts transfer TGs to luSCC cells, and TG breakdown is required for cholesterol synthesis and luSCC invasion. Inhibition of the cholesterol synthesis pathway decreases luSCC invasion in vitro. To confirm the role of cholesterol synthesis in luSCC progression in vivo, we tested whether statins (atorvastatin) inhibit luSCC in animal luSCC tumours. For this, we developed tumours injecting luSCC cells alone, or tumours by co-injecting luSCC cells and fibroblasts (luSCC + lung FB) into the flanks of NSG mice on oral atorvastatin. This confirmed that animals with luSCC + lung FB tumours had reduced survival compared to animals with luSCC tumours alone; critically, atorvastatin significantly decreased luSCC + lung FB tumour growth and improved survival, while having no impact on luSCC tumours alone (Fig. 6f and Extended Data Fig. 7d,e).

## Lung fibroblast lipid metabolism impacts early-stage luSCC development and outcome

To study the impact of fibroblast programmes in human luSCC, we compared molecular datasets of established luSCC to early-stage luSCC, focusing on lipid and cholesterol metabolism. Specifically, we studied the lipid metabolic pathways in bronchial premalignancy, dysplasia and in situ and invasive luSCC, which confirmed a progressive increase in cholesterol synthesis gene expression with histological disease progression (Fig. 6g). To further validate that lipid metabolism contributes to luSCC progression, we next compared lipid metabolism pathways in early-stage in situ SCC that progressed to invasive SCC, to in situ SCC that regressed. This confirmed a significantly higher expression of cholesterol genes expressed in in situ lesions that progressed to SCC compared to in situ lesions that regressed (Fig. 6h).

To test the impact of lipid metabolism on established luSCC lesions, we explored the correlation between lipid droplets, fibroblast programmes and patient outcomes in human luSCC. This revealed that gene expression of the lipid droplet-associated protein PLIN2 is correlated to fibroblast signatures, and this was confirmed in a second human luSCC cohort (Fig. 6i,j). Given that hypoxia can induce lipid droplet formation, we examined whether *PLIN2* expression correlated with hypoxic signalling. In the TCGA cohort, *PLIN2* showed no association with hypoxia and, although a small but significant correlation was observed in the TRACERx dataset, it was weaker than the association with fibroblast-related signatures (Extended Data Fig. 7f,g). Critically, higher *PLIN2* expression significantly predicts worse overall survival in TCGA (Fig. 6k), and a fibroblast signature in the TRACERx lung cohort is strongly correlated with tumour progression (Fig. 6l) and poor overall survival (Extended Data Fig. 7h). Additionally, higher ATGL expression (*PNPL2A*) in luSCC, which is linked to TG breakdown, correlated with shorter disease-free survival (Fig. 6m,n) and worse overall survival in both cohorts (Extended Data Fig. 7i,j). These data collectively indicate genes involved in fibroblast and lipid metabolism, lipid droplets and cholesterol synthesis are associated with luSCC outcome.

**Fig. 7:**
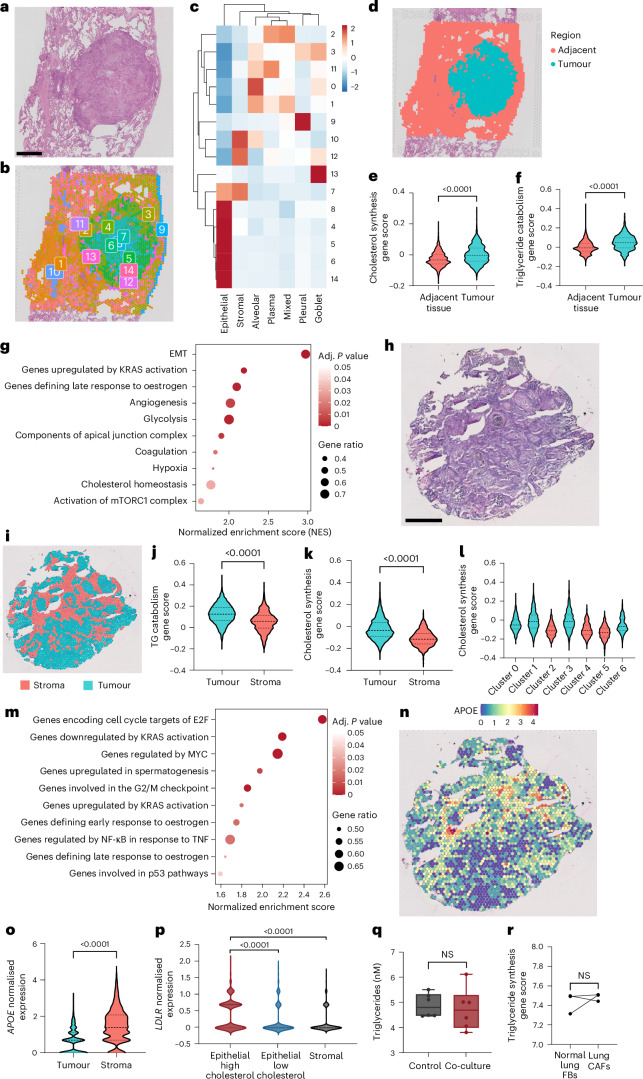
Spatial mapping of cholesterol synthesis in luSCC tumour regions with colocalization of epithelial cancer cells and fibroblasts. **a**, H&E stain of luSCC tumour for spatial transcriptomics (*n* = 2 independent tumours analysed, one section per tumour; scale bar, 1 mm). **b**, Colour map of gene expression-based clusters representing distinct spatial transcriptional signatures in luSCC. **c**, Heat map showing overlap between clusters (rows) and cell-type signatures (columns) in luSCC spatial transcriptomics. Colour scale represents *z*-scaled signature score (blue, low; red, high). **d**, Clusters illustrating epithelial tumour signature cluster (blue) and adjacent tissue (red) based on cell-type signature clustering. **e**, Quantification of cholesterol synthesis gene signature in tumour cluster (blue) and adjacent tissue (red; two-sided Mann–Whitney *U-*test, tumour *n* = 1,010 spots, adjacent *n* = 2,418 spots). **f**, Quantification of TG catabolism gene signatures in tumour cluster (blue) and adjacent tissue (red; two-sided Mann–Whitney *U-*test, tumour *n* = 1,010 spots, adjacent *n* = 2,418 spots). **g**, Dot plot of the top ten pathways from gene-set enrichment analysis of marker genes defining clusters containing fibroblasts and epithelial cells (cluster 7). Spot size indicates gene ratio. Colour represents adjusted *P* value (enrichment significance assessed using a permutation-based test implemented in clusterProfiler; *P* values were adjusted for multiple comparisons with the Benjamini–Hochberg method). **h**, H&E stain of a second luSCC tumour for spatial transcriptomics (*n* = 2 independent tumours analysed, one section per tumour; scale bar, 1 mm). **i**, Clusters illustrating epithelial tumour signature cluster (blue) and adjacent stroma cluster (red) based on cell-type signature clustering. **j**,**k**, Quantification of TG catabolism (**j**) and cholesterol synthesis (**k**) gene signatures in tumour cluster (blue) and adjacent tissue (red; two-sided Mann–Whitney *U-*test, tumour *n* = 1,494 spots, stroma *n* = 875 spots). **l**, Cholesterol synthesis gene signature score across spatial transcriptomic clusters; blue indicates epithelial tumour clusters, and red indicates stomal clusters. **m**, Dot plot of the top ten pathways from gene-set enrichment analysis of genes enriched in high-cholesterol tumour clusters (cluster 1 and 3). Spot size represents gene ratio. Colour represents adjusted *P* value (enrichment significance assessed using a permutation-based test implemented in clusterProfiler; *P* values were adjusted for multiple comparisons with Benjamini–Hochberg method). **n**, Spatial distribution of *APOE* expression in luSCC tumour. Colour denotes expression (blue, low; red, high). **o**, Quantification of *APOE* in the tumour and stromal compartment of the second luSCC tumour (two-sided Mann–Whitney *U-*test, tumour *n* = 1,494 spots, stroma *n* = 875 spots). **p**, Quantification of *LDLR* expression in tumour compartment group by high (cluster 1 and 3) or low (cluster 0 and 6) cholesterol status and the stromal compartment of luSCC tumour (Kruskal–Wallis, Dunn’s multiple comparisons, high *n* = 834 spots, low *n* = 660 spots, stromal *n* = 875 spots). **q**, TGs quantified in the secretomes of lung fibroblasts co-cultured with luSCC cells (two-sided Mann–Whitney *U-*test, tumour *n* = 6 biological replicates). **r**, TG synthesis pathway ssGSEA scores in matched normal lung fibroblasts and CAFs from individuals with lung cancer (GSE244065, two-sided Mann–Whitney *U-*test, tumour *n* = 3 biological replicates). Box plots show the minimum to maximum values (error bars), box indicates the 25th and 75th percentiles, and the line denotes the median. Bar plots indicate the mean ± s.e.m. Violin plots show the data distribution, dashed lines indicate quartiles, and the dashed line denotes the median. Source data

**Extended Data Fig. 8:**
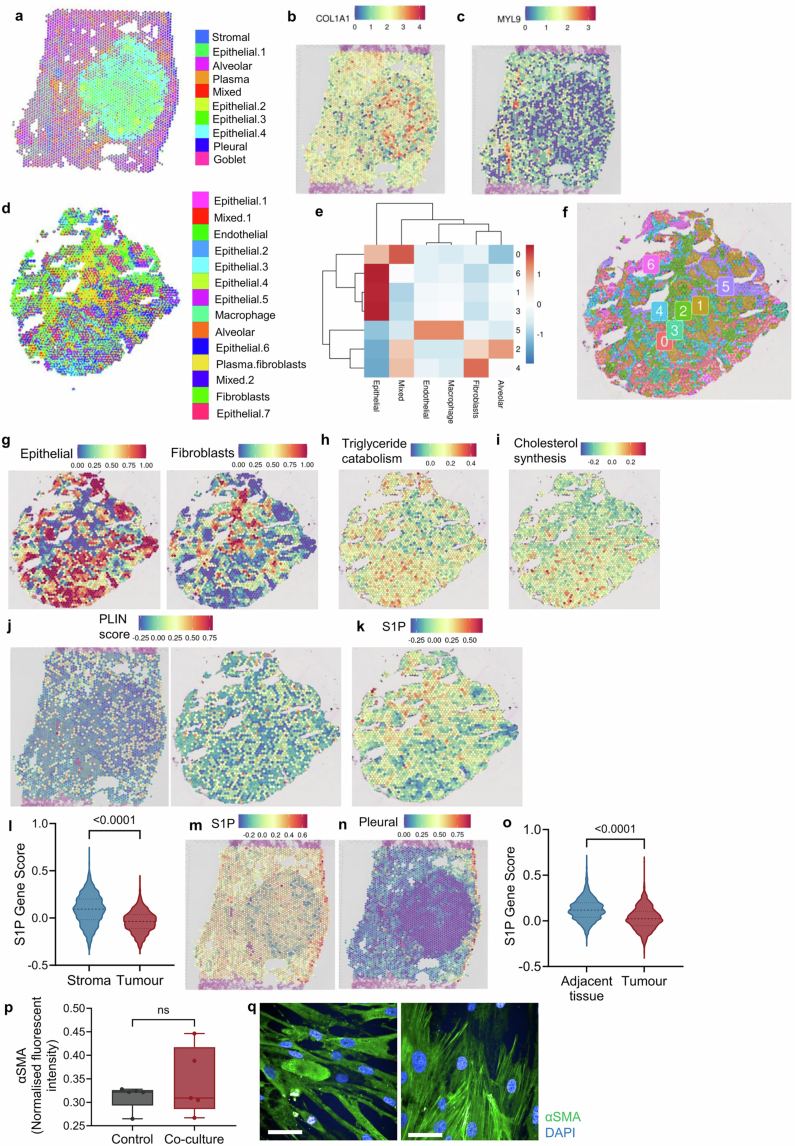
Triglyceride and cholesterol biology in lung SCC tumours. **a**, Spatial distribution of deconvoluted cell type signatures in human lung SCC sample, each spot represents pie chart displaying proportion of each signature represented by colours. **b**, COL1A1 (left, fibroblast), and **c**, MYL9 (right, smooth muscle) stromal gene expression in luSCC spatial transcriptomics, colour: gene expression (low: blue, red: high). **d**, Spatial distribution of deconvoluted cell type signatures in second human lung SCC sample, each spot represents pie chart displaying proportion of each signature represented by colours. **e**, Heatmap showing overlap between deconvoluted cell type signatures and gene expression-based clusters (**f**) representing distinct spatial transcriptional signatures in lung SCC spatial transcriptomics (colours represent distinct clusters). **g**, Spatial transcriptional expression of epithelial cell signatures (left) and spatial fibroblast signature (right) second lung SCC tumour, colour: proportion of each spot represented by signature (blue: low, red: high). **h**, Spatial distribution of triglyceride catabolism and cholesterol synthesis (**i**) gene signatures in second lung SCC sample, colour: proportion of each spot represented by signature (blue: low, red: high). **j**, Spatial distribution of lipid droplet PLIN gene signature score in lung SCC tumours, colour: signature expression (low: blue, red: high). **k**, Spatial distribution and quantification (**l**) of S1P signalling gene signature in tumour (red) and stromal (blue) compartments of second lung SCC tumour (two-sided Mann Whitney U). **m**, Spatial distribution of S1P and **n**, pleural signatures in first lung SCC tumour. **o**, Quantification of S1P gene signature in tumour compartment and adjacent tissue of first lung SCC tumour (two-sided Mann Whitney U). **p**, Immunofluorescence quantification of alpha smooth muscle actin (αSMA) in lung fibroblasts co-cultured with lung SCC cell lines (two-sided Mann Whitney U, n = 5 biological replicates, ns: non-significant). **q**, Representative immunofluorescence images of αSMA (green) in control (left) and co-cultured (right) lung fibroblasts (representative image from n = 5 biologically independent wells; 122 fields per well analysed, scale: 25 µm). Boxplots: minimum to maximum values (error bars), box: 25th and 75th percentiles; line: median value. Source data

To explore the spatial relationship between fibroblasts and luSCC in humans, we performed spatial transcriptomics of an early-stage luSCC tumour (Visium 10x Genomics; Fig. 7a). Deconvolution analysis revealed ten distinct cell-type signatures, which could be broadly categorized into epithelial, stromal, alveolar, goblet, pleural and plasma cells (Fig. 7b,c, Extended Data Fig. 8a and Supplementary Table 10). While the stromal signature was enriched for endothelial, smooth muscle and fibroblast genes, the fibroblast markers were primarily enriched within the tumour compared to endothelial and smooth muscle markers, which were localized to blood vessels in tumour-adjacent tissue. These spatial distributions indicate that the predominant stromal signature in the tumour is derived from fibroblasts (Extended Data Fig. 8b,c). We next confirmed that genes related to TG breakdown and cholesterol synthesis were significantly higher in the tumour cluster than in the surrounding adjacent tissue (Fig. 7d–f) and, strikingly, where fibroblasts clustered with epithelial cells (cluster 7), we observed significant gene enrichment of cholesterol metabolism and EMT pathways (Fig. 7g and Supplementary Table 11). We validated these findings spatially in a second luSCC tumour (Fig. 7h). Implementing deconvolution of cell-type signatures (Extended Data Fig. 8d–g), we generated sample clusters in tumour regions containing epithelial cells, as well as stromal regions containing fibroblast, macrophage, endothelial and alveolar signatures (Fig. 7i). This confirmed that TG breakdown and cholesterol synthesis pathway genes were more highly expressed in tumour regions (Fig. 7j,k and Extended Data Fig. 8h,i). Two epithelial clusters displayed higher cholesterol synthesis gene signatures (clusters 1 and 3; Fig. 7l), and a further examination of the pathways that were enriched in these cholesterol-high tumour clusters (Supplementary Table 12) revealed enriched oncogenic signalling via TNF, KRAS and MYC, and cell cycle pathways (Fig. 7m). Additionally, *APOE* expression was highly enriched in the stroma containing lung fibroblasts (Fig. 7n,o), and epithelial tumour regions with high cholesterol synthesis expression had significantly higher expression of *LDLR*, which encodes a receptor that can bind and internalize APOE (Fig. 7p). To study lipid droplets in the lung parenchyma and tumour, we examined the spatial expression of the lipid droplet-associated gene signature (PLINs), which showed PLIN gene expression in both the tumours and the adjacent tissue (Extended Data Fig. 8j). Conversely, the S1P signalling genes, which are specific drivers of EMT and invasion in oSCC tumours, were not activated in lung tumours, further confirming the tissue specificity of lipid pathways (Extended Data Fig. 8k–o). We next explored the impact of lung fibroblast activation on TG biology. Importantly, activation of normal lung fibroblasts through co-culture with SCC cells did not significantly alter TG levels (Fig. 7q and Extended Data Fig. 8p,q), and in matched normal lung fibroblasts and CAFs, there was no significant difference in the expression of TG synthesis pathway genes (Fig. 7r). These data suggest that fibroblast activation within the lung tumour does not impact TG synthesis.

**Extended Data Fig. 9:**
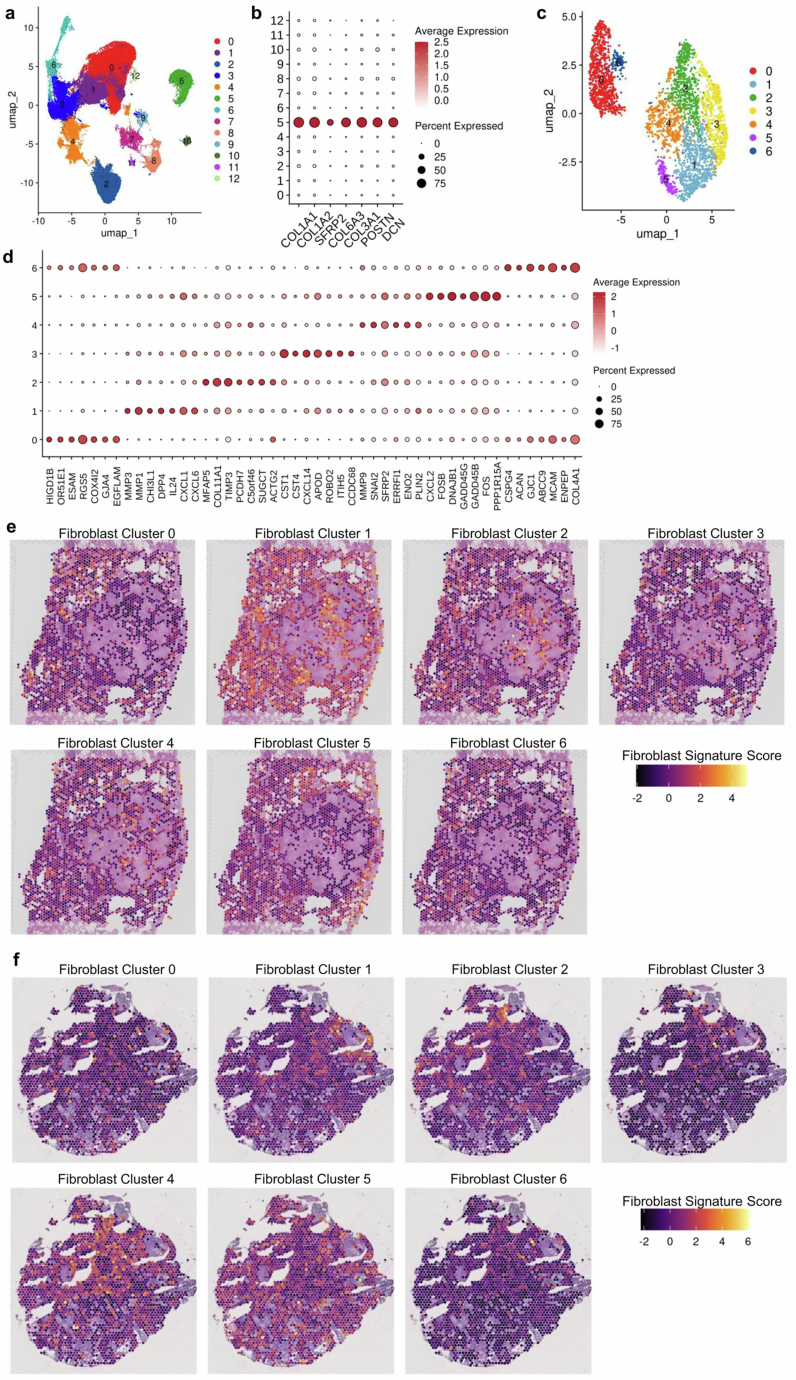
Fibroblast heterogeneity in lung SCC. **a**, UMAP plot of single cell RNA sequencing dataset (GSE148071) of lung SCC tumours. **b**, Dot plot of fibroblast marker genes showing enrichment of fibroblasts in cluster 5. **c**, UMAP plot of fibroblasts (cluster 5) subclustered into distinct fibroblast populations. **d**, Dot plot of the expression of top seven marker genes for each fibroblast cluster, size of dot represents percent of cells gene expressed in, colour represents expression level (white: low, red: high). **e**, Expression of each fibroblast clusters marker gene signature in spatial transcriptomics lung SCC tumour fibroblast regions (colour represents expression levels, black: low, yellow: high). **f**, Expression of each fibroblast clusters marker gene signature in second spatial transcriptomics lung SCC tumour fibroblast regions (colour represents expression levels, black: low, yellow: high).

Exploring fibroblast heterogeneity further, we identified mixed populations of fibroblasts in the human luSCC tumours (Extended Data Fig. 9 and Supplementary Table 13). Fibroblasts expressing prominent inflammatory signatures (cluster 1) and extracellular matrix (cluster 2) CAF markers were enriched primarily in the first tumour (Extended Data Fig. 9e,f). In contrast, the normal fibroblast cluster (cluster 4), which included the expression of the lipid droplet gene *PLIN2*, was enriched in the stromal regions of both tumours. Thus, our samples indicate that lung tumours contain fibroblasts displaying both normal and CAF phenotypes, and that CAF phenotypes preserve TG metabolism signatures. Altogether, these data show that lung fibroblasts transfer TGs to fuel cholesterol synthesis and luSCC progression, which correlates with poor patient outcomes.

## Lipid metabolism in human cSCC is downregulated and correlates with improved survival

**Fig. 8:**
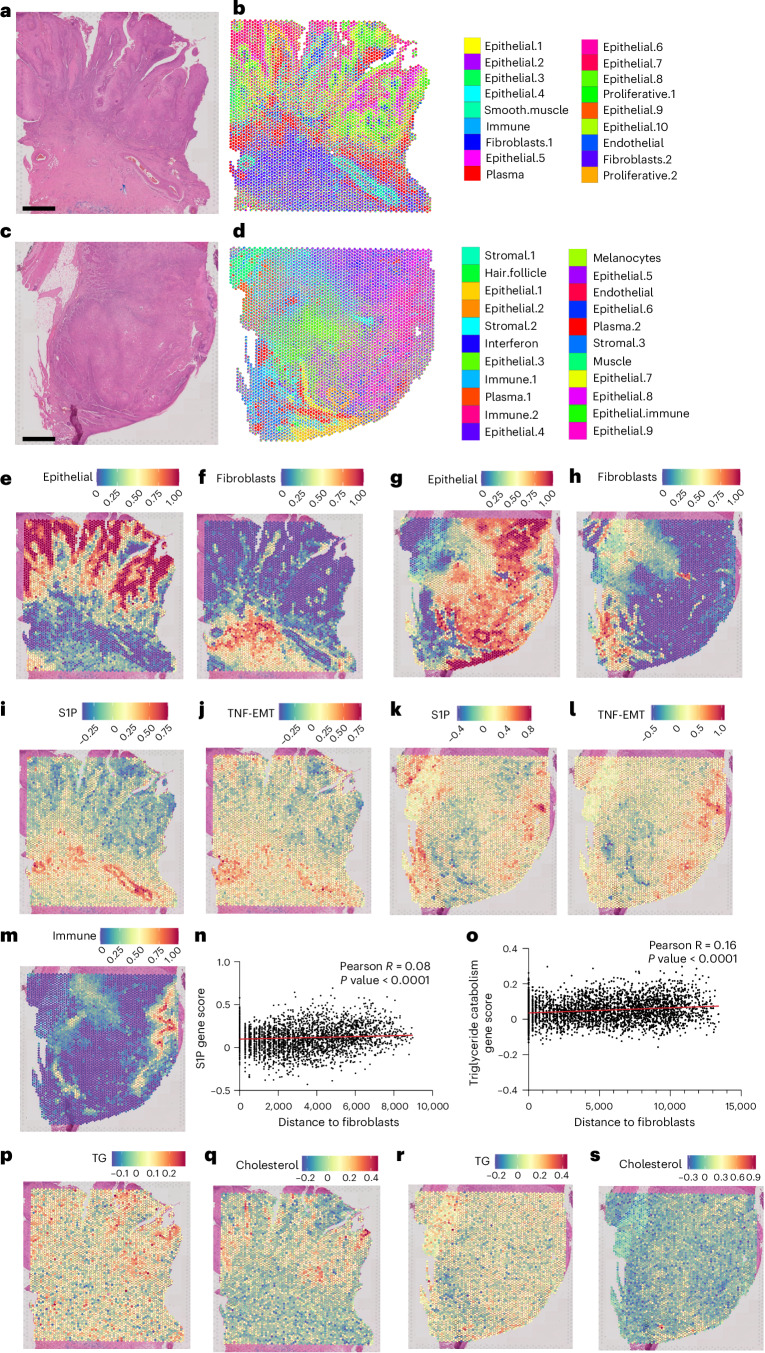
Tissue-specific lipid metabolism pathways in cSCC tumours. **a**,**b**, Human cSCC tumour H&E stain (*n* = 2 independent tumours analysed, one section per tumour; scale bar, 1 mm) (**a**) and corresponding spatial distribution of deconvoluted cell-type signatures (**b**). Each spot represents a pie chart displaying the proportion of each signature represented by colours. **c**,**d**, Second human cSCC tumour H&E stain (*n* = 2 independent tumours analysed, one section per tumour; scale bars, 1 mm) (**c**) and corresponding spatial distribution of deconvoluted cell-type signatures (**d**). **e**,**f**, Spatial transcriptional expression of epithelial cell signatures (**e**) and spatial fibroblast signature of the first cSCC tumour (**f**). Colour indicates the proportion of each spot represented by the signature (blue, low; red, high). **g**,**h**, Spatial transcriptional expression of epithelial cell signatures (**g**) and spatial fibroblast signature of the second cSCC tumour (**h**). Colour indicates the proportion of each spot represented by the signature (blue, low; red, high). **i**–**l**, Spatial expression of S1P gene signature and TNF-EMT signature in the first (**i** and **j**) and second (**k** and **l**) cSCC tumours. Colour indicates the expression of the signature (blue, low; red, high). **m**, Spatial transcriptional expression of immune cell signatures in the second cSCC tumour. Colour indicates the proportion of each spot represented by the signature (blue, low; red, high). **n**, Correlation between S1P signature expression in epithelial-enriched spots and distance to fibroblast-enriched clusters in the second cSCC tumour (two-sided Pearson correlation, *n* = 3,314). **o**, Correlation between TG catabolism signature expression in epithelial-enriched spots and distance to fibroblast-enriched clusters in the first cSCC tumour (two-sided Pearson correlation, *n* = 3,870). **p**–**s**, Spatial expression of TG catabolism and cholesterol synthesis signatures in the first (**p** and **q**) and second (**r** and **s**) cSCC tumours. Colour indicates the expression of the signature (blue, low; red, high). Source data

**Extended Data Fig. 10:**
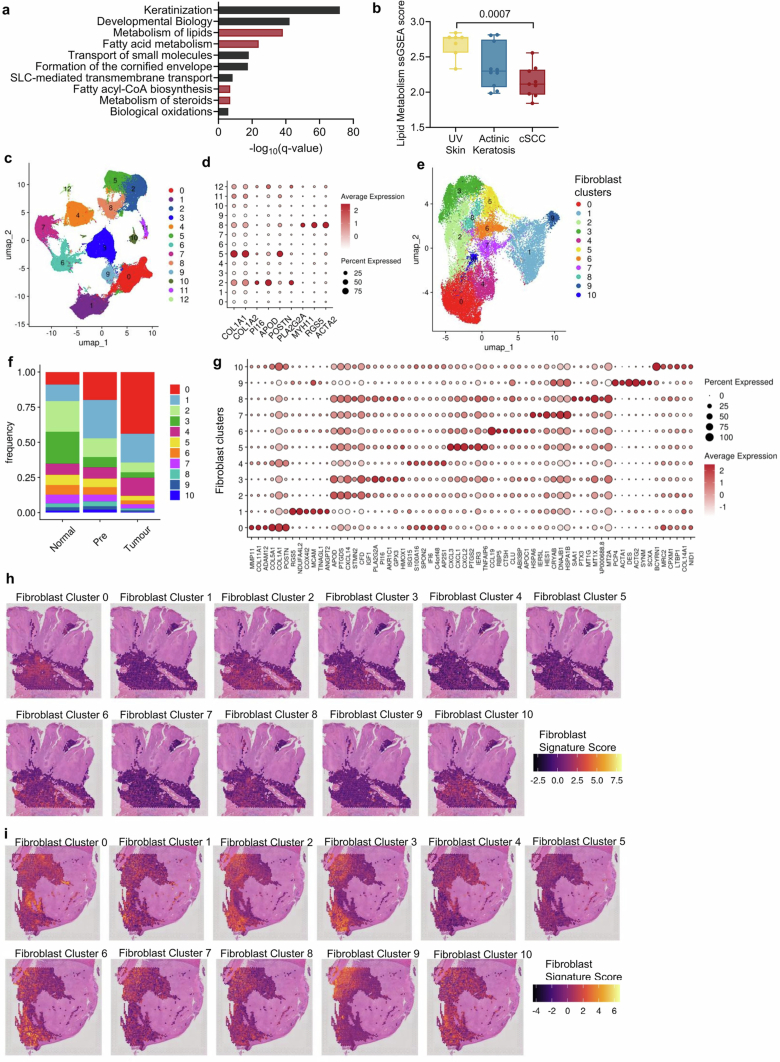
Lipid metabolism and fibroblast heterogeneity in human cSCC. **a**, Biological pathways significantly downregulated with progression from UV damaged skin to cSCC, red bars: lipid metabolism pathways. **b**, Single sample gene set enrichment analysis (ssGSEA) score of lipid metabolism genes in ultraviolet radiation (UV) exposed skin, actinic keratosis (in situ cSCC), and cutaneous SCC (cSCC) samples (GSE84293, two-sided Mann Whitney U, n = 7 skin, n = 10 actinic keratoses, n = 9 cSCC), boxplots: minimum to maximum values (error bars), box: 25th and 75th percentiles, line: median. **c**, UMAP plot of cSCC single cell RNA sequencing dataset (GSE218170) containing cSCC tumours, premalignancy (Bowen’s disease) and normal skin. **d**, Dot plot of fibroblast marker genes showing enrichment of fibroblasts in cluster 2, 5 and 8. **e**, UMAP plot of fibroblasts (cluster 2, 5, 8) subclustered into distinct fibroblast populations. **f**, Bar plot displaying the frequency of each fibroblast population in cSCC tumour, premalignancy (pre) and normal skin. **g**, Dot plot of the expression of top six marker genes for each fibroblast cluster, size of dot represents percent of cells gene expressed in, colour represents expression level (white: low, red: high). **h**, Expression of each fibroblast clusters marker gene signature in spatial transcriptomics cSCC tumour fibroblast regions (colour represents expression levels, black: low, yellow: high). **i**, Expression of each fibroblast clusters marker gene signature in second spatial transcriptomics cSCC tumour fibroblast regions (colour represents expression levels, black: low, yellow: high). Source data

Dermal fibroblasts are lipid poor and do not induce invasive programmes on SCC in vitro, so we examined the relationship between lipid metabolism, dermal fibroblasts and progression in human cSCC tumours. We compared lipid metabolic pathways in aged, sun-damaged skin and cSCC, which revealed that sun-damaged skin had higher expression of lipid pathways compared to in situ cSCC and invasive cSCC (Extended Data Fig. 10a,b). These data show that unlike non-cutaneous SCC, lipid metabolism is downregulated in cSCC progression. To explore this further, we performed spatial transcriptomics on two cSCC tumours (Visium 10x Genomics), identifying regions of epithelial tumours and fibroblasts (Supplementary Table 14), and testing whether the tissue-specific pathways that drive oral and lung SCC (Fig. 8a–h) are absent in cSCC. This confirmed that S1P and TNF-EMT signalling, which are enriched in oSCC, are only present in cSCC endothelial and immune-enriched regions, but not in established cSCC tumours or near dermal fibroblasts (Fig. 8i–n). We next examined whether TG and cholesterol pathways, which fuel luSCC progression, are present in the cSCC tumours. This revealed that only the well-differentiated, keratinizing cSCC tumour expressed TGs and cholesterol metabolic signatures; however, the expression was spatially localized to the epithelial, well-differentiated, non-invasive tumour compartment with no association with fibroblast signatures (Fig. 8o–s), a finding that is expected during epidermal differentiation of the stratum corneum. Finally, cSCC samples also expressed a heterogeneous mix of normal and CAF populations (Extended Data Fig. 10c–i and Supplementary Table 15), with no differences in lipid biology. Taken together, these findings establish in human tissue a spatial link between tissue-specific lipid metabolic cues, imposed by site-specific fibroblast lipid programmes, and epithelial cancer invasion, metastasis and survival.

## Discussion

We examined the contribution of tissue-resident fibroblasts from different anatomic sites on the invasive properties and phenotypes of SCCs. We observed that fibroblasts from different anatomic sites harbour distinct lipid compositional profiles that align with site-specific transcriptional states. Fibroblasts are metabolically dynamic cells that can release lipids and other metabolites into their microenvironment. Lipids serve diverse roles, including fuelling cellular metabolism, contributing to membrane biogenesis, acting as signalling mediators and modulating protein behaviour and localization. We found that fibroblasts from the oral cavity and the lung synthesize and secrete more lipids, and lipids drive invasive oral and lung SCCs, which are highly metastatic. In contrast, dermal fibroblasts are lipid poor, limiting cSCC invasion, which correlates with the low metastatic rate of these tumours in individuals. Firstly, we showed that oral fibroblasts secrete lipid signalling SMs, which in oSCC cells activate the SM–ceramide–S1P–STAT3 pathway to drive oSCC invasion. In animals, oSCC cells co-injected with oral fibroblasts have decreased survival compared to animals injected with oSCC cells alone. Secondly, we found that lung fibroblasts produce and secrete more lipids and TGs, which transfer to luSCC cells where they are broken down and utilized to synthesize cholesterol and fuel luSCC progression. We show that inhibiting cholesterol synthesis with atorvastatin in luSCC decreases invasion and improves animal survival. In human tissue, oSCC tumours in close spatial proximity to tissue-resident fibroblasts highly express the SM pathway and, in the lung, tumour cells spatially close to fibroblasts express TGs and cholesterol lipid metabolic pathways. Importantly, these site-specific lipid programmes are linked to progression and survival across multiple patient cohorts. Taken together, this work shows that fibroblast anatomic origin affects SCC biology and outcome, and stromal fibroblasts impose the rate of SCC invasion via lipid metabolic cues. Furthermore, targeting metabolic stromal fibroblast–cancer cell dependencies decreases epithelial cancer progression in vivo.

Fibroblasts are present throughout the body and play a crucial role in epithelial and connective tissue support, epithelial function and identity, wound healing and tissue repair. Lipids are key to adult fibroblast identity and heterogeneity^42^, and our work adds to the current knowledge showing that fibroblasts from different sites have specific biological properties and exert unique effects on epithelial cancer progression. Specifically, the site-specific secretory lipid component of fibroblasts determines SCC invasion.

This work defines a new oral fibroblast contribution to oSCC progression. During early stages of oSCC development, oral fibroblasts may provide SMs to adjacent epithelial cancer cells for invasion, and our analysis of human data presented here supports that fibroblast metabolic interactions with epithelial cancer cells define early head and neck carcinogenesis. In support of our work, *Sphk1*^−/−^ mice have reduced head and neck SCC numbers and tumour burden in carcinogenesis models^43^. Intriguingly, patients on antidepressants such as imipramine that inhibit acid sphingomyelinase^44^ have a lower risk of oSCC and other cancers^45,46^, and we show that imipramine reduces oSCC invasion.

SPHK1 drives more aggressive disease in oSCC. Previous studies established oSCCs express SPHK1 primarily at the invasive front of tumours, in close association with fibroblasts. Furthermore, increased SPHK1 expression correlates with clinical stage, lymph node metastasis, EMT markers and lower oSCC survival^47,48^. Our study identifies stromal oral fibroblasts as the source of SMs that promote oSCC invasion. Mechanistically, SM transfer from oral fibroblasts to oSCC cells upregulates the TNF/EMT invasion signature and the phosphorylation of STAT3, which are pathways that lie downstream of SPHK1 in aggressive oSCC^49–51^.

We defined a new contribution of stromal lung fibroblasts to aggressive luSCC behaviour. We show that lung fibroblasts produce high levels of TGs, which transfer via transport proteins to adjacent luSCC cells in lipid droplets. The breakdown of TGs into fatty acids in luSCC, which are used for cholesterol synthesis, promotes luSCC invasion. We show this hallmark is a tissue-specific phenotype, as increasing the lipid content in dermal fibroblasts does not increase TG transfer or SCC invasion. Previous work established that lipid droplets in lung fibroblasts have the unique site function of neonatal lung epithelial development, providing support under hyperoxic conditions and supporting the production of surfactant phospholipids^52,53^. Importantly, aggressive non-small cell lung cancer also prominently activates lipid metabolic pathways, suggesting that lipid droplet transfer from lung-resident fibroblasts may contribute to lung cancer subtypes more widely^54–56^. Critically, preliminary work has shown that increased lipid droplets drive activation of lung fibroblasts to CAFs^57^, and cigarette smoke increases ROS in exposed lungs and lipid droplet formation in lung fibroblasts^58^. Our work focuses on normal tissue fibroblasts; however, future studies should address lipid metabolism dependencies in the established tumour microenvironment between CAFs and later stages of SCC.

Additionally, we show that statins decrease tumour volume and improve survival in mice bearing tumours composed of luSCC cells and lung fibroblasts. Epidemiological and functional work has previously shown conflicting results on the potential of statins to improve cancer outcomes; however, veterans and patients with diabetes who are on statins have lower lung cancer and luSCC incidence, respectively^59,60^. Our work suggests that statins may reduce early disease progression from dysplasia and carcinoma in situ to invasive luSCC.

Finally, we show that dermal fibroblasts do not transfer high quantities of lipids to adjacent cSCC cells, which limits the ability of cSCC to invade. These findings mirror human data, which confirm that lipid metabolic reliance is not a hallmark of progressing cutaneous dysplasia and human cSCC tumours have a very low metastatic rate (1–3%)^17^. Thus, we link the favourable outcome observed in human cSCC to decreased lipid metabolic reliance and downregulation of lipid metabolism, which limits cSCC progression^61^. In contrast, oral and lung SCC, which rely on lipid metabolism and obtain site-specific lipids from fibroblasts, have poor rates of survival (45–80% 5-year mortality)^16^.

Our study has limitations that warrant consideration. First, although we identify a site-specific activation of the cholesterol biosynthetic programme in luSCC and demonstrate its functional contribution to invasion, the precise metabolites and downstream effectors responsible for this phenotype remain incompletely defined. We cannot yet distinguish whether increased invasiveness is driven by cholesterol itself, by intermediate mevalonate-pathway metabolites or by broader sterol-dependent signalling processes. Moreover, while our data link fibroblast-derived TGs to the induction of SREBP2 and cholesterol synthesis, we have not formally shown that fatty acids liberated from these TGs are directly channelled into sterol biosynthesis.

Second, in vitro systems do not capture the structural and metabolic complexity of stromal–epithelial interfaces in vivo. Our in vitro assays provide a permissive environment that supports fibroblast viability and lipid biosynthesis, yet they do not reproduce the restricted diffusion, oxygen gradients and spatial organization that shape fibroblast–epithelial metabolic coupling in tissues. For this reason, we incorporated orthogonal validation with in vivo and human models. The development of more physiologically informed culture systems is important to resolve the dynamics of fibroblast-derived lipid provision in native tissue contexts.

Our work shows that tissue-resident fibroblast lipids interact with epithelial malignant cells in a tissue-specific manner to determine SCC invasion. This work provides new therapeutic rationales and targets for epithelial cancer prevention, biomarker development and cancer therapies.

## Methods

### Cell lines and culture

Two normal adult oral fibroblasts (CTICC1.8.2) were purchased from Generon and three normal adult lung fibroblasts (CC-2512) were purchased from Lonza. One oral fibroblast cell line was acquired from C. Gaudy, Aix-Marseille Université. Two cSCC cell lines, IC1 and IC19 (ref. ^62^), were acquired from C. Harwood from Queen Mary University London, two oSCC cell lines FADU and UMSCC01 were acquired from C. West, The University of Manchester, and two luSCC cell lines, SKMES1 and H520 (NCI-H520), were acquired from C. Lopez-Garcia, Cancer Research UK Manchester Institute. All cell lines were cultured in DMEM (Gibco, 41966-029) supplemented with 10% FCS (Sigma-Aldrich, F7524), 1× Glutamax (Gibco, 35050061), 100 U ml^−1^ penicillin and streptomycin (Gibco, 15140122) and 1 mM sodium pyruvate (Gibco, 11360070). For fatty acid-treated fibroblasts, media were prepared as above supplemented with 50 µM oleic acid (Cayman Chemical, 90260) and 100 µM palmitic acid (Cayman Chemical, 10006627) and fibroblasts were cultured in fatty acid-supplemented media for 5 days. All cell lines were cultured at 37 °C in 5% CO_2_ with medium replaced as required. Cell lines were tested every month for *Mycoplasma* using LookOut Mycoplasma PCR Detection Kit (Sigma-Aldrich, MP0035). The cell line identity of SCC cell lines was confirmed using STR profiling.

### Human fibroblasts

Dermal fibroblast cultures were established from redundant normal skin (surgical dog ears) of individuals treated at the Christie NHS Foundation Trust. Ethical approval to establish fibroblast cell lines from the redundant normal skin was granted by the local Biobank committee (17_AMVI_01), which required signed informed consent from all participants. The hypodermis of whole-skin samples was removed by scraping with a scalpel, and the residual specimen was incubated overnight in Dispase (Gibco, 17105-041) at 4 °C to separate the epidermis and dermis. The dermis was digested in Collagenase I (Gibco, 17018029) in DMEM (without FCS) at 37 °C for 6 h and then filtered through a 70-µm filter to remove the residual debris. Dermal cells were spun at 300*g* and resuspended in DMEM 20% FCS, cultured in DMEM 20% FCS until they became confluent.

### Secretome collection

To collect secretomes, 1 × 10^6^ fibroblast cells were plated in a 100-mm dish and the following day the medium was washed off twice with PBS and then cultured for 72 h in 10 ml DMEM without FBS (Zen-Bio, DMEMHG-PRF) to limit cell proliferation. This medium was then collected as secretomes and centrifuged at 1,000*g* for 5 min, aliquoted and stored at −80 °C until used. For required experiments, lipids were removed from the secretomes using Cleanascite Lipid Removal Reagent (Biotech Support Group, X2555-100). Cleanascite was mixed at room temperature and diluted in secretome at a 1:5 ratio and incubated for 10 min with gentle shaking. Samples were centrifuged at 16,000*g* for 1 min and the supernatant was collected. Fresh lipid-stripped secretomes and control media were prepared before each experiment.

### Fibroblast co-culture models

For co-culture models, fibroblasts were cultured in six-well plates with 100,000 cells per well containing 3.0-µm PET membrane inserts (Corning, 353092) containing 200,000 SCC cells from tissue-matched fibroblasts. Cells were co-cultured for 7 days with media replaced regularly. When SCCs grew to confluence in inserts, they were replaced with a new insert with 200,000 cells. To collect secretomes, after co-culture inserts with SCCs were removed, fibroblasts were washed twice with PBS and serum-free DMEM was added to wells. Secretomes were collected from co-cultured fibroblasts after 72 h as previously outlined. Control fibroblasts were cultured in identical conditions without SCC cells in inserts. Once secretomes were collected, fibroblasts were fixed in 4% paraformaldehyde for 20 min at room temperature. To account for changes in fibroblast proliferation in co-culture conditions, relative confluence was measured with crystal violet staining. Fixed fibroblasts were washed with PBS and stained with 0.05% crystal violet (V5265, Sigma-Aldrich) for 30 min. Excess crystal violet was washed off with PBS and cells were lysed with 1% SDS (Invitrogen, 15553-035) for 30 min. Crystal violet levels from each co-culture sample were quantified by measuring absorbance at 595 nm on a VarioSkan Lux plate reader (Thermo Fisher Scientific) and SkanIt Software (Research edition, version 7.0.2) and raw OD values were used to normalize differences in cell density between control and co-culture cells. For imaging of activation markers, fibroblasts were seeded in 96-well plates and cultured in SCC cell line conditioned media for 7 days, with fresh conditioned media added three times. Fibroblasts were then washed with PBS and fixed with 4% paraformaldehyde and used for immunofluorescence as described below.

### Treatment of cells

SM (Egg-SM, Avanti Polar Lipids, 860061P) was resuspended in ethanol at 50 mM stock concentration. SM was diluted to final concentrations in serum-free DMEM for experiments. Imipramine hydrochloride (Tocris, 7841) was resuspended in DMSO at a stock concentration of 50 mM and diluted in secretomes to 20 µM. S1P (Tocris, 1370) was resuspended in 0.3 M NaOH at a stock concentration of 5 mM and diluted in serum-free DMEM for use. ABC294640 (APExBIO, B1182) was resuspended in DMSO to a concentration of 10 mM and used in secretomes at a final concentration of 20 µM. PF543 (Sigma-Aldrich, PZ0234) dissolved in double distilled water to a stock concentration of 19.92 mM and diluted in secretomes to a final concentration of 2 µM. ARC39 (Cayman Chemical, 13583) was dissolved in PBS to a stock concentration of 3.58 mM. Carmofur (RayBiotech, 331-10309) was supplied at 10 mM in DMSO and diluted in secretomes to 5 µM or 10 µM. S1PR1 inhibitor ponesimod (MedChemExpress, HY-10569-1ml) and S1PR3 inhibitor TY-52156 (MedChemExpress, HY-19736-1ml) were supplied at 10 mM in DMSO and diluted in secretomes to 5 µM or 10 µM. STAT3 inhibitor STX-0119 (MedChemExpress, HY-103692-1ml), AKT inhibitor MK-2206 (MedChemExpress, HY-10358-1ml) and NF-κB inhibitor JSH-23 (MedChemExpress, HY-13982-1ml) were supplied at 10 mM in DMSO and diluted to 20 µM, 2 µM and 10 µM, respectively, in secretomes. Triacsin C (LKT Labs, T6834) was resuspended in DMSO to a stock concentration of 766 µM. Atglistatin (Sigma-Aldrich, 5301510001) was resuspended at 10 mM in DMSO and diluted to 20 µM or 50 µM in secretomes or serum-free media. Cells were treated with atglistatin overnight in serum-free media before treatment with fibroblast secretomes containing atglistatin. *N*-acetyl-L-cysteine (Sigma-Aldrich, A9165) was dissolved in double distilled water at a stock concentration of 100 mM and diluted in media to final concentrations. Atorvastatin (Stratech, S2077-SEL) was resuspended in DMSO at 5 mM and diluted in secretomes to a 5 µM final concentration. Mevalonate (Cayman Chemical, 37476) was resuspended in DMSO at 50 mM and diluted to 500 µM in secretomes. Nervonic acid (Cayman Chemical, CAY13940-100) was resuspended in DMSO at 54.56 mM, and heneicosanoic acid (Cayman Chemical, CAY22593-100) was resuspended in ethanol at 61.24 mM. For treatment of cells with fatty acids in rescue experiments, 2 mM (nervonic and heneicosanoic acid) and 5 mM (oleic and palmitic acid) stock solutions were prepared in serum-free DMEM containing 10% BSA, which was used for further dilutions in secretomes for final working concentrations. In all treatment conditions, vehicle controls were used that matched the dilutions of the treatment conditions.

### Collagen organotypic invasion assays

SCC cell line invasion into collagen with fibroblasts was assayed using a protocol adapted from Timpson et al.^63^. Briefly, equal numbers of dermal, oral or lung fibroblasts (150,000 cells per collagen disc) were mixed with 2.5 mg ml^−^^1^ Collagen I, rat tail (Corning, 354236) and cultured in 35-mm culture dishes. Collagen discs were allowed to contract until they fit in a 24-well plate. Cell suspensions of SCC cell lines at 4 × 10^4^ cells per ml were plated on top of each collagen disc in duplicate for each fibroblast cell line. Cells and collagen were cultured as normal for approximately 5 days in DMEM. Collagen discs were then transferred to a Falcon 3.0-µm high-density PET membrane (Corning, 353092) in Falcon six-well Deep Well TC-treated Polystyrene Plates (Corning, 355467) containing DMEM to create an air–liquid interface to drive SCC invasion into collagen. After 12 days, constructs were fixed in 4% paraformaldehyde and embedded in paraffin and stained with H&E. Slides were scanned at ×20 with an Olympus VS200 slide scanner (Olympus). Invasion of cells into collagen was quantified with HALO software (Indica labs, version 3.6.4134.314). A supervised random forest classifier was generated and trained to distinguish cells from collagen on the slides. Invasive area was calculated as the area of cells within the collagen normalized to the total area of collagen analysed. Two sections per collagen disc were analysed for two independent discs per condition.

### Spheroid invasion assays

SCC cell lines were cultured in U-bottom 96-well plates (Brand, 781900) at 5 × 10^3^ cells per well, spun at 200*g*, and spheroids were allowed to form over 72 h. Culture medium was removed from the wells, and 100 µl collagen (PureCol, Advanced BioMatrix, 5005-100 ML) diluted to 1.5 mg ml^−1^ in serum-free DMEM was added to the wells. Plates were briefly spun for 5 s at 200*g* and incubated at 37 °C for 1 h to set collagen. One hundred µl secretomes or control medium was added on top of the collagen once set, plates were incubated, and cell invasion was monitored over 96 h. For experiments with pharmacological inhibitors or rescue metabolites, these were resuspended in secretomes or medium added on top of spheroids. Images of spheroids were taken with light microscopy (EVOS XL Core, Thermo Fisher Scientific, AMEX1000) at ×4 magnification under constant light settings. Invasion of cells into collagen was quantified with HALO software (Indica labs, version 3.6.4134.314). A supervised random forest classifier for each cell line was generated and trained to distinguish the spheroid core, invading cells and collagen. Invasive area was calculated as the area of invasive cells as a percentage of the total area of invasive cells and spheroid core combined, to normalize for spheroid size.

### In vivo subcutaneous co-injection model

All procedures involving animals were performed under the Home Office-approved project license PP0466403, and UK Home Office regulations under the Animals (Scientific Procedures) Act 1986. The study received ethical approval by the Cancer Research UK Manchester Institute’s Animal Welfare and Ethics Review Body (AWERB). All mice were maintained in pathogen-free, ventilated cages in the Biological Resources Unit at our Institute, and allowed free access to irradiated food and autoclaved water in a 12-h light–dark cycle, with room temperature at 21 ± 2 °C. All cages contained wood shavings, bedding and a plastic tube for environmental enrichment. Experiments were performed in adults (8–12-week-old) female NSG mice (Charles River) acclimatized for 7 days before study. Four cohorts—oSCC (UMSCC01 cell line, 21 mice, 7 per group; FADU cell line, 12 mice, 6 per group; shCtrl 6 mice; and sh*SPHK1* 6 mice) and luSCC (SKMES1; 24 mice, 8 per group) were used. All cell lines were tested for *Mycoplasma* and murine hepatitis virus and were confirmed negative before injection. Tumours were generated by co-injection of SCC and fibroblasts at a 1:3 ratio, with 100,000 SCC cells and 300,000 fibroblasts per injection. On the day of injection, cells were collected and washed twice in ice-cold PBS and then counted, aliquoted in ice-cold PBS at the correct ratio, washed once more in PBS then resuspended in Cultrex UltiMatrix Reduced Growth Factor Basement Membrane Extract (Bio-techne, BME001-05) at a volume of 50 µl per injection. All preparations for injections were performed on ice and needles and syringes were stored on ice before injection. Cells were injected subcutaneously in the right flanks of animals.

Mice treated with statins were given oral atorvastatin in sterile PBS at 20 mg per kg body weight daily by oral gavage once tumours were palpable after injection. Mice treated with imipramine were given imipramine hydrochloride (Sigma-Aldrich, I0899) in sterile PBS at 40 mg per kg body weight daily by oral gavage once tumours were palpable. Animal tumour development was closely monitored, and once tumour development began tumours were measured three times weekly. Animals were culled by schedule 1 (cervical dislocation) if tumours reached a volume limit of 1,500 mm^3^, or if a tumour interfered with the quality of life before this point. Full-body autopsy including liver, heart, lungs, liver, spleen and kidneys was conducted on all mice. Tumours and organs were collected and fixed in 10% formalin overnight before embedding in paraffin and stained with H&E, and then histologically analysed blinded by group by dermatologist and dermatopathologist to assess tumour features (L.M. and A.V.). Tumour growth rate was calculated as final tumour volume divided by the number of days to reach final volume. In the luSCC study, 12 animals did not reach limits and were culled and censored 184 days from the beginning of the study to conclude the study. In the imipramine treatment model, one mouse (oSCC + oral FB) was culled 7 days into the study due to leg injury and was censored. Two animals in the lung SCC study did not develop tumours following injection and were excluded from analysis (1× luSCC, 1× luSCC + lung FB). Group sizes (*n* = 6–8 per group) were selected based on prior in vivo tumour growth experiments from our laboratory and published studies demonstrating that this number provides sufficient power to detect biologically meaningful differences in tumour burden while minimizing animal use. Assuming a 35% difference in tumour volume between groups, a standard deviation of 25%, a two-sided *α* of 0.05 and 80% power, a minimum of six mice per group were required. To account for potential attrition, 7–8 mice per group were used in some experiments.

### Mouse lung fibroblast extraction

Lung fibroblasts were extracted and cultured from the lungs of wild-type and *Apoe*^−/−^ C57BL/6 mice (male, 8 weeks of age). *Apoe*^−/−^ mice were obtained from the University of Manchester. The Apoetm1Unc mutant strain (B6.129P2-Apoetm1Unc/J) was originally developed in the laboratory of N. Maeda at The University of North Carolina at Chapel Hill to replace part of exon 3 and part of intron 3 of the apolipoprotein E (*Apoe*) gene replaced with a neomycin resistance (neo) cassette. The genotype of each mouse was confirmed using the Transnetyx system. Healthy disease-free mice were culled and lungs removed during autopsy and stored in ice-cold serum-free DMEM. Immediately after, lungs were incised in a Petri dish with scalpel to relax the tissue and transferred to a tube containing 3 U ml^−^^1^ collagenase P (Merck, 11213857001) and 20 U ml^−^^1^ DNase I (Sigma-Aldrich, D4263) in 5 ml DMEM and incubated at 37 °C for 15 min with intermittent shaking. After incubation, tissue was dissociated with sequential pipetting with 25-ml, 10-ml and 5-ml pipettes. Following this, incubation and dissociation were repeated in this manner two more times. Samples were then centrifuged at 450*g* for 5 min, the supernatant removed, and the pellet resuspended in 1 ml TrypLE Express (Thermo Fisher, 12604013) and incubated at 37 °C for 10 min. Samples were strained on ice through 40-µm strainers (EASYStrainer, Grener Bio-One, 542040) and washed with serum-free DMEM. Samples were centrifuged at 450*g* for 5 min at 4 °C and the pellet resuspended in 1 ml red blood cell lysis solution (BioLegend, 420301) and incubated on ice for 10 min. Following this, samples were centrifuged at 450*g* for 5 min at 4 °C, and the pellet was resuspended in complete 10% DMEM and cultured as described above.

### Immunohistochemistry

Slides were stained on the BOND RX automated platform (Leica Microsystems). Sections (4 µm) of FFPE tumours were cut and mounted on charged slides. Dewaxing and heat-induced epitope retrieval of slides was automated on the Bond RX, using Epitope Retrieval Solution 1 (ER1 AR9961, Leica Microsystems) for 20 min at 98 °C (Antigen Retrieval Ph6). Using the Refine kit (Leica Microsystems, DS9800), endogenous peroxidase was blocked using the peroxidase in the Refine kit) for 10 min and the slides further blocked with 10% wt/vol casein (2b Scientific SP-5020) in TBS-T. Antibody application (Ki67 Agilent M7240, 0.23 µg ml^−^^1^, 1:200 dilution; human mitochondria, ab92824, 1 µg ml^−^^1^), detection incubation with the primary antibody in Bond Antibody Diluent (Leica Microsystems, AR9352) for 30 min, followed by detection using Mouse Envision (K4001 Agilent) and the Refine Kit (DS9800). Slides were dehydrated through graded ethanol, cleared in xylene and cover-slipped with Pertex. Slides were scanned at a magnification of ×20 with an Olympus VS200 slide scanner (Olympus). Analysis of Ki67 staining was performed using HALO software (Indica labs, version 3.6.4134.314) IHC area quantification algorithm. For collagen organotypic analyses, Ki67-positive nuclei were calculated as a percentage of total nuclei to provide a normalized measure of varying amounts of cells by cell line. For tumour analysis, the number of Ki67-positive cells were counted in two hotspots per tumour counted by two independent scorers.

### Mouse lipid extraction

To extract lipids from fresh mouse tissues, the tongue/pharynx, lungs and abdominal skin were collected from four wild-type disease-free C57BL/6 mice (male, 8 weeks of age). Tissues were lysed in 10 mg ml^−^^1^ isopropanol using a QIAGEN TissueLyser II and placed in −80 °C for 1 h before centrifuging at 21,000*g* for 10 min at 4 °C, and supernatant collected.

### Lipidomics

To quantify lipids in fibroblast secretomes, to 600 μl of fibroblast secretome, 60 μl of 10% formic acid was added, followed by the addition of 400 μl of methanol for protein precipitation. After vortexing, samples were centrifuged at 20,000*g* for 10 min at 4 °C. The supernatant was transferred to a glass tube and 100 μl of chloroform was added. After 10 min resting at 4 °C, the bottom layer containing the lipids was transferred to a new glass tube. The chloroform extraction was repeated to ensure recovery of the lipids. The extracts containing lipids were dried under nitrogen stream and reconstituted in 30 μl of methanol for HPLC–MS analysis. Lipids were separated on a Dionex Ultimate 3000 UHPLC (Thermo Fisher Scientific) coupled to a Q Exactive plus Hybrid Orbitrap MS (Thermo Fisher Scientific). Around 5 μl of the sample was loaded on a Kinetex 2.6 u EVO C18 100 A, LC Column 150 × 0.3 mm (Phenomenex). A gradient was performed at 250 μl min^−1^ using H_2_O-5mM ammonium formate, 0.1% formic acid (A), 60% acetonitrile: 40% methanol-5mM ammonium formate, 0.1% formic acid (B) and isopropanol-5mM ammonium formate, 0.1% formic acid (C). The start condition was 85% (A), 15% (B), kept for 0.5 min increasing to 100% (B) at 7.5 min, kept from 1 min and increasing to 95% (C) and 5% (B) at 19 min and kept until 23 min. At 24 min, returned to 100% (B) and kept for 1 min. Returned to the initial condition at 27 min until 30 min. Data were acquired in positive ion mode with full MS/dMS2 using a scan range from 300 *m/z* to 1,800 *m/z*. Full MS resolution of 70,000 and MS2 of 17,500 were used. For lipidomics on fresh mouse tissue, 600 μl of mouse tissue extract, was prepared as secretomes above. Lipids were separated on a Dionex Ultimate 3000 UHPLC (Thermo Fisher Scientific) coupled to a Q Exactive Hybrid Orbitrap MS (Thermo Fisher Scientific). Around 5 μl of the sample was loaded on a Kinetex 2.6 u EVO C18 100 A, LC Column 150 × 0.3 mm (Phenomenex). A gradient was performed at 200 μl min^−1^ using H_2_O-5mM ammonium formate, 0.1% formic acid (A), 60% acetonitrile: 40% methanol-5mM ammonium formate, 0.1% formic acid (B) and isopropanol-5mM ammonium formate, 0.1% formic acid (C). The start condition was 75% (A), 25% (B), kept for 0.5 min increasing to 100% (B) at 2 min and increasing to 95% (C) and 5% (B) at 7 min and 99% (C), 1% (B) at 10 min and kept until 11.5 min. At 12 min, returned to 100% (B) and kept for 0.5 min. Returned to the initial condition at 13 min until 15 min. Data were acquired in positive ion mode with full MS/ddMS2 using a scan range from 250 *m/z* to 1,700 *m/z*. Full MS resolution of 70,000 and 17,500 for MS2 were used.

### Lipid assays

TGs were quantified in the secretomes of fibroblasts using the Triglyceride Assay Kit (Abcam, ab65336) using the fluorescence protocol and 50 µl of secretomes per well. SM was quantified using the Sphingomyelin Assay Kit (Cell Biolabs, STA-601) and 10 µl of secretomes per well. Secretomes were quantified from two independent secretomes from three independent fibroblast cell lines per tissue group, measured in technical triplicate. Fluorescence was measured using a VarioSkan Lux plate reader (Thermo Fisher Scientific) and SkanIt Software (Research Edition, version 7.0.2).

### APOE ELISA

APOE was quantified in fibroblast secretomes using the Human APOE ELISA Kit (Invitrogen, EHAPOE). In total, 10 ml of each fibroblast secretome was concentrated using Amicon Ultra-15 centrifugal filter units (Millipore, UFC901024), and 100 µl per well of 1:10 diluted concentrated secretomes were run in the ELISA. Absorbance was detected using a VarioSkan Lux plate reader (Thermo Fisher Scientific) and SkanIt Software (Research edition, Version 7.0.2). Absorbances of blank wells were subtracted from all values and unknowns were interpolated from sigmoidal, four-parameter logistic standard curve. A value below the blank was considered undetectable.

### Seahorse metabolic assay

Cell metabolism was measured using the Seahorse XFe96 Analyzer system (Agilent Technologies). Before the assay, the cartridge was hydrated for 24 h with distilled water and an hour before the experiment, water was replaced with 200 μl per well of Seahorse XF Calibrant Solution (Agilent Technologies, 100840-000) and incubated in a non-CO_2_ 37 °C incubator for 1 h. Cells were washed twice with PBS and incubated in Seahorse XF base medium (Agilent Technologies, 103335-100) supplemented with 2 mM L-glutamine, 10 mM glucose and 1 mM sodium pyruvate, and incubated in a non-CO_2_ incubator at 37 °C for 1 h. OXPHOS and mitochondrial function were assessed in cell lines using the Seahorse XF Cell Mito Stress Test kit (Agilent Technologies, 103015-100). OCR was measured across the assay with sequential injection of 1.5 µM oligomycin, 2 µM FCCP and 0.5 µM antimycin A–rotenone. For all assays, to normalize for cell number, after the assay was complete media were aspirated from the wells and cells were fixed in 4% paraformaldehyde for 20 min. Fixed cells were washed with PBS and stained with 0.05% crystal violet (V5265, Sigma-Aldrich) for 30 min. Excess crystal violet was washed off with PBS and cells were lysed with 1% SDS (Invitrogen, 15553-035) for 30 min. Crystal violet levels were quantified by measuring absorbance at 595 nm on a VarioSkan Lux plate reader (Thermo Fisher Scientific) and SkanIt Software (Research edition, Version 7.0.2), and raw OD values were used to normalize Seahorse results for each plate in the Wave software (Agilent Technologies, version 2.6.1.53). Normalized results were exported from the software using the report generator, and basal respiration was used as measurement of OXPHOS in each condition.

### Mitochondria visualization and quantification

Mitochondrial activity was quantified by immunofluorescence using MitoView Green (Biotium, 70054-T) to quantify mitochondrial abundance and MitoView 633 (Biotium, 70055-T) to quantify mitochondrial membrane potential. SCC cells were plated in 96-well plates 24 h before treatment with control serum-free media or oral fibroblast secretomes. Each condition was measured in four replicate wells with two fibroblast secretomes per SCC cell line. After 24 h of secretome treatment, cells were washed with PBS and incubated in serum-free DMEM containing 100 nM MitoView Green or MitoView 633, and 1 µg ml^−1^ Hoechst 33342 (Thermo Fisher Scientific, 62249) for 30 min at 37 °C. Cells were washed twice with PBS and fresh serum-free DMEM was added to each well. Cells were imaged live with using an Opera Phenix (Perkin Elmer) with Zeiss C-Apochromat ×63 water immersion objective NA 1.15 WD 0.6 mm, with 134 fields of view per well. Images were processed in Harmony software (Perkin Elmer, version 6.9). Briefly, nucleus staining was used to identify cells based on Hoechst staining using the predefined methods in Harmony. For quantification, the mean fluorescence signal was calculated per well and divided by the number of nuclei to normalize for differences in cell numbers.

### Cholesterol assay

Intracellular cholesterol levels in SCC cells were quantified using the Cholesterol/Cholesterol Ester-Glo Assay (Promega, J3190). SCC cells were plated in opaque white 96-well plates and treated with atglistatin or control for 24 h before treatment with lung fibroblast secretomes containing atglistatin or vehicle control for 24 h. Each condition was measured in triplicate wells using two different fibroblast secretomes. Cells were lysed with the cholesterol lysis solution, and total cholesterol levels were quantified alongside a standard curve with cholesterol detection reagent with esterase. Luminescence was quantified with a VarioSkan Lux plate reader (Thermo Fisher Scientific) and SkanIt Software (Research edition, version 7.0.2). Total cholesterol was quantified by interpolation from a linear standard curve.

### Intracellular lipid staining

Fluorescence visualization of intracellular lipids was performed using BODIPY 493/503 (Cayman Chemical, 25892) or LipidSpot 610 (Biotium, 70069). BODIPY was dissolved in DMSO to 5 mg ml^−1^. Cells were incubated with BODIPY or LipidSpot diluted at 1:1,000 in serum-free DMEM containing 1 µg ml^−1^ Hoechst 33342 (Thermo Fisher Scientific, 62249) and incubated at 37 °C for 30 min. Cells were washed with PBS twice and fixed with 4% paraformaldehyde for 20 min at room temperature before imaging. For lipid transfer analysis, fibroblasts were incubated with BODIPY as above for 30 min at 37 °C. BODIPY media was removed and cells washed three times with PBS to remove excess BODIPY. Cells were cultured in fresh serum-free DMEM for 48 h and media collected as secretomes containing labelled lipids. SCC cells were cultured in these labelled secretomes for 24 h before removing secretomes and staining with 1 µg ml^−1^ Hoechst in serum-free DMEM at 37 °C for 20 min. Cells were washed with PBS and fixed with 4% paraformaldehyde for 20 min at room temperature before imaging. Imaging was performed using an Opera Phenix (Perkin Elmer) with Zeiss C-Apochromat ×63 water immersion objective NA 1.15 WD 0.6 mm, with at least 146 fields of view per well. Images were processed in Harmony software (Perkin Elmer, version 6.9). Briefly, analysis sequence was developed for each analysis based on the signals. Nucleus staining was used to identify cells based on Hoechst staining using the predefined methods in Harmony. Lipid spots were calculated using the Find Spots feature. The output of the data was calculated as lipid spots per cell per well by dividing average lipid spots per nucleus in a well.

### Incucyte proliferation, viability and ROS quantification

Proliferation of SCC cells was quantified using the confluence assay on an IncuCyte S3 (Essen Bioscience). luSCC cells were plated in clear-bottom 96-well plates in four replicate wells per cell line and treated with serum-free DMEM and lung fibroblast secretomes, with or without the addition of 5 µM atorvastatin. oSCC cells were plated in five replicate wells per cell line and treated with serum-free DMEM with or without 20 µM SM, 20 µM imipramine or 2 µM PF543. Plates were incubated at 37 °C and imaged in real time at ×10 for phase-contrast images over 72 h at 2-h intervals with four images per well. Phase masking and AI confluence calculation of confluence were used to compare growth rates of cell lines in each condition using the IncuCyte software (Essen Bioscience, 2020C rev. 1). Relative confluence was calculated by dividing the confluence of the cells with the initial confluence of the cells. Viability was quantified using propidium iodide diluted in secretomes or media at 1 µg ml^−1^ and imaged in real time at ×10 for phase-contrast and red fluorescence images over 24 h at 2-h intervals with four images per well. Image segmentation of the red channel used Top-Hat background correction (radius 100, threshold 2.0, Edge Split ON, sensitivity = 0). The count of dead cells was normalized to the confluence of their respective wells. ROS levels in fibroblast cell lines were quantified using the CellROX Green reagent (Thermo Fisher, C10444) in an IncuCyte S3 (Essen Bioscience). Fibroblasts were plated in clear-bottom 96-well plates in four replicate wells per cell line and treated with DMEM containing 5 µM CellROX Green. Plates were incubated at 37 °C and imaged in real time at ×10 for phase-contrast and green fluorescence images over 24 h at 2-h intervals with four images per well. Image segmentation of the green channel used Surface Fit background correction with Edge Split ON, Threshold = 0 and Edge Sensitivity = 0. The software-reported Total Integrated Green Intensity was normalized to phase confluence for each field to correct for differences in cell number.

### Immunofluorescence

Cells for immunofluorescence were plated in 96-well plates treated with experimental conditions. Cells were fixed with 4% paraformaldehyde for 20 min at room temperature before permeabilization with 0.01% Triton X-100 for 5 min. Cells were washed three times with Tris buffered saline (TBS) and blocked with 5% BSA TBS-T (5% BSA in 1× TBS, 0.1% Tween 20) for 1 h at room temperature. Cells were incubated overnight with primary antibodies diluted in 1% BSA TBS-T (1% BSA in 1× TBS, 0.05% Tween 20) at 4 °C (S1P, 1:500 dilution, Echelon Biosciences, Z-P300; APOE, 1:1,000 dilution, Abcam, ab183597; αSMA, 1:200 dilution, Abcam, ab7817; IL-6 1:200 dilution, Abcam, ab233706). Cells were washed three times with TBS and then incubated with secondary antibodies for 1 h at room temperature (goat anti-rabbit Alexa Fluor 488, 1:1,000 dilution, Thermo Fisher, A32731; goat anti-mouse Alexa Fluor 488, 1:1,000 dilution, Thermo Fisher, A32723; goat anti-rabbit Alexa Fluor 555, 1:2,000 dilution, Thermo Fisher, A32732). For experiments with actin staining, cells were incubated with Alexa Fluor 546 Phalloidin (1:2,000 dilution, Invitrogen, A22283) at the same time as secondary antibodies. Cells were washed three times with TBS and incubated with 500 nM DAPI (Invitrogen, D3571) in TBS at room temperature for 20 min. For experiments also including lipid droplet staining, cells were stained with BODIPY (1:1,000 dilution) for 20 min with DAPI staining. Cells were washed with TBS before imaging. All experiments contained negative control wells that were treated identically to experimental wells except for primary antibody staining. S1P immunofluorescence contained a positive control of cells treated with exogenous S1P as positive control. Imaging was performed using an Opera Phenix (Perkin Elmer) with a Zeiss C-Apochromat ×63 water immersion objective NA 1.15 WD 0.6 mm, with at least 146 fields of view per well. Images were processed in Harmony software (Perkin Elmer, version 6.9). Briefly, the analysis sequence was developed for each analysis based on the signals and controls. Nucleus staining was used to identify cells based on DAPI staining using the predefined methods in Harmony. For quantification, the mean fluorescence signal was calculated per well and divided by the number of nuclei to normalize for differences in cell numbers.

### Western blots

Protein was extracted from SCC cell pellets using RIPA Lysis Buffer (Thermo Fisher Scientific, 88901) containing 1× Phosphatase inhibitor (Roche, 4906845001) and 1× Protease inhibitor (Roche, 11836153001) by incubating on ice for 30 min with regular vortexing, followed by centrifuging at 14,000*g* for 15 min at 4 °C and the supernatant collected. For cytoplasmic and nuclear fractions, proteins were extracted with the NucBuster Protein Extraction Kit (Millipore, 71183-M). Protein samples were quantified using the Pierce BCA Protein Assay Kit (Thermo Fisher Scientific, 23225). A total of 50 µg of protein was diluted in Laemmli Buffer (Bio-Rad, 1610747) with beta-mercaptoethanol (Sigma-Aldrich, M6250), denatured at 95 °C for 5 min and loaded onto Mini-PROTEAN TGX Gels (Bio-Rad, 4568084) and ran in 1× TGS buffer (Bio-Rad, 1610732) at 100 V. Samples were transferred to nitrocellulose membranes using the Trans-Blot Turbo system (Bio-Rad, 170-4270) and protein visualized with Ponceau Stain (Sigma-Aldrich, P7170). Membranes were blocked in 5% BSA TBS-T (5% BSA in 1× TBS, 0.1% Tween 20) for 1 h and incubated with primary antibodies overnight in 5% BSA TBS-T (STAT3, 1:1,000 dilution, mouse mAb 9139, and phosphor-STAT3 Tyr705, 1:500 dilution, rabbit mAb 9145, Cell Signalling Technologies; B-actin, 1:10,000 dilution, mouse mAb ab8226, Abcam; SREBP2, 1:1,000 dilution rabbit pAb ab30682, Abcam; SPHK1 1:1,000 dilution; rabbit mAb 12071, Cell Signalling Technologies; GAPDH 1:5,000 dilution, rabbit mAb 5174, Cell Signalling Technologies; TATA BP 1:2,000 dilution rabbit mAb ab220788, Abcam; vinculin 1:10,000 dilution, rabbit mAb ab129002, Abcam). Membranes were washed three times with TBS-T and incubated in secondary antibodies (Anti-Rabbit IgG, HRP-linked Antibody, Cell Signalling, 7074 and Anti- Mouse IgG, HRP-linked Antibody, Cell Signalling, 7076, 1:5,000 dilution) in 5% BSA TBS-T for 1 h at room temperature. Membranes were washed three times with TBS-T and were visualized using the ECL Western Blotting Detection Reagents (Merck, Cytiva RPN2209) and blots were imaged using the Bio-Rad ChemiDoc imaging system using the optimal autoexposure settings. Quantification of bands was performed by densitometry analysis using ImageJ software (version 1.53o) to normalize protein bands to their respective loading controls. Phospho-protein quantification was presented as a ratio of normalized phosphorylated protein to normalized total protein.

### qPCR

RNA was collected in triplicate from 8 × 10^5^ cells and were processed and RNA extracted using RNeasy Mini Kit (Qiagen, 74104). Concentration was determined with the Qubit RNA BR Assay (Invitrogen, Q10210) and 500 ng RNA was reverse transcribed to cDNA using TaqMan Reverse Transcription Reagents (Thermo Fisher, N8080234) and diluted at a 1:20 ratio in nuclease-free water. Genes were quantified by qPCR using TaqMan Gene expression assays and Fast Mastermix (Thermo Fisher, 4444556) on a QuantStudio 3 system. *GAPDH* (Hs02758991_g1) and *ACTB* (Hs01060665_g1) were used as housekeeping genes. *VIM* (Hs00958111_m1), *FN1* (Hs01549976_m1), *SNAI2* (Hs00161904_m1) and *SPHK1* (Hs00184211_m1) were quantified and normalized to the geometric mean of both housekeeping genes and relative expression calculated using 2^−Δct^.

### Lentiviral shRNA transfection

Knockdown of *SPHK1* expression in FADU cells was performed using shRNA Lentiviral Particles (Santa Cruz Biotechnology). For *SPHK1* knockdown, SPHK1 shRNA (h) lentiviral particles (sc-44114-V) were used alongside a scramble control, Control shRNA Lentiviral Particles A (sc-108080). A total of 5 × 10^4^ cells were cultured in cell culture media with 5 μg ml^−1^ Polybrene (Santa Cruz Biotechnology, sc-134220). Lentiviral particles were added to cells and incubated overnight. Media containing lentiviral particles and Polybrene was removed, and cells were incubated in DMEM overnight before performing selection of transfected cells using increasing concentrations of puromycin over 72 h. Once cells were stably growing in puromycin, cells were cultured as normal in DMEM. Knockdown of *SPHK1* gene expression was validated by qPCR and western blot.

### RNA sequencing

For fibroblast RNA sequencing, RNA was extracted from 400,000 fibroblasts cultured in six-well plates for 72 h in serum-free DMEM to match secretome collection conditions. For SCC RNA sequencing, IC19, UMSCC01 and SKMES1 were plated at 80% confluence and treated with fibroblast secretomes for 24 h or serum-free DMEM control in triplicate. RNA was extracted using RNeasy mini kits (Qiagen, 74104) and quantified with Qubit RNA broad range assay (Thermo Fisher, Q10210). Sequencing was performed by GENEWIZ (Azenta Life Sciences). Libraries were prepared with polyA selection and sequenced on 2 × 150-base-pair paired-end reads. SCC sequencing was performed at approximately 20 million reads per sample. Fibroblasts were sequenced using a strand-specific protocol with approximately 30 million reads per sample. The RNA-sequencing reads underwent quality checking using the FASTQC programme. Then, Trim Galore v. 0.6.10 was utilized to inspect and remove adaptor contamination. Subsequently, the clean reads were aligned to the human reference genome assembly (GRCh38) using the STAR aligner v.2.5.1b^64^ in either single-end or paired-end mode based on the sequencing protocol, with default parameters. The mapped data were then converted to gene-level integer read counts using featureCounts^65^, and the Ensemble gene annotation (Homo_sapiens.GRCh38.85.gtf). Differential expression analysis was performed using the DESeq2 (version 1.28.1)^66^ packaged in R (version 4.3.0, RStudio Pro 2024.04.2, RStudio). Analysis was performed on protein-coding genes and reads counts were filtered by removing any gene with less than ten counts across all samples. Downstream analysis was performed with Ingenuity Pathway Analysis software (version 111725566, Qiagen) to identify enriched biological pathways and upstream regulators.

### Spatial transcriptomics

The oSCC human specimens analysed by spatial transcriptomics were obtained from the CCR5396 ORIGINS Study. The ORganoid GeneratioN Study for Cancer (ORIGINS, NCT05734963) was reviewed and approved by The Committee for Clinical Research at The Royal Marsden Hospital (ref. CCR5396) and the North Tyneside 1 Research Committee (ref. 21/NE/0096, IRAS 292105). cSCC specimens were obtained from Northern Care Alliance NHS, ethical approval Health Research Authority (IRAS 216310, REC ref. 16/LO2098, sponsored by The University of Manchester). The human luSCC specimens were obtained from the Christie NHS Foundation Trust Biobank under ethical approval granted by the local Biobank committee (17_AMVI_01). Indexed sequencing libraries were prepared from H&E-stained Visium Spatial Gene Expression Slides using the Visium Spatial for FFPE Gene Expression Kit, Human Transcriptome (10x Genomics, 1000338, 55-µm resolution, untargeted transcriptomics), according to the manufacturer’s protocol. Library quality was checked using the Fragment Analyzer (Agilent). Libraries were quantified by qPCR using a KAPA Library Quantification Kit for Illumina (Roche, 07960336001). Paired-end sequencing with read lengths of 28 + 10 + 10 + 50 base pairs was performed on a NovaSeq 6000 sequencer (Illumina). Data were processed through the standard Space Ranger pipeline. Data for the second luSCC sample were downloaded from BioStudies (E-MTAB-13530). Deconvolution analysis for cell-type signatures in tumours was performed with the STdeconvolve package (version 1.4.0)^67^ standard workflow. This package performs a reference-free cell-type deconvolution analysis using latent Dirichlet allocation modelling to determine the optimal number of cell signatures. For analysis, spots with fewer than 100 transcript counts were removed, and genes that are expressed in 100% of spots or present in fewer than 5% of spots were removed. To find the optimal number of cell-type signatures (topics) for each sample, a range of latent Dirichlet allocation models were fitted with between 5 and 25 cell types. The optimal number of cell-type signatures (*k*) was determined by the model with the lowest perplexity (oSCC: sample 1 *k* = 13, sample 2 *k* = 12; luSCC: sample 1 *k* = 10, sample 2 *k* = 14; cSCC: sample 1 *k* = 18, sample 2 *k* = 22). Marker genes for each signature were determined as the top 20 genes with the highest log_2_FC compared to other signatures (Supplementary Tables 6, 10 and 14). The filtered Space Ranger output was further processed and analysed in the Seurat package (version 5.1) in R (version 4.3.0). All samples were analysed with the standard Seurat spatial transcriptomics workflow individually. Firstly, spots with fewer than 100 transcripts detected were removed from samples and cell-type signature (topic) percentages from STdeconvolve analysis were added to the object metadata. Each sample was normalized with the SCTransform function and PCA was performed. Dimensionality reduction and clustering were performed on the first 30 components, and clusters were identified at a resolution of 0.6 for all samples except for 1.0 for luSCC sample 1. To calculate the distance of each spot to fibroblasts at the invasive front, the *x* and *y* coordinates were extracted with the GetTissueCoordinates function. For each spot, the minimum distance to any spot of fibroblasts at the invasive front was calculated by computing the Euclidean distance from each spot to each invasive front fibroblast spot and recording the smallest distance. Gene signatures were calculated using the AddModuleScore function. Gene-set enrichment analysis of marker genes for Seurat clusters was performed using the clusterProfiler package (version 4.8.3) and compared to Hallmark pathways by converting gene names to Entrez IDs, ranking genes by log fold change and compared to Hallmark pathway database using the msigdbr (version 7.5.1), org.Hs.eg.sb (version 3.17.0) and enrichplot (version 1.20.3) packages.

### Single-cell RNA-sequencing data analysis

Data for single-cell RNA sequencing was downloaded from NCBI’s Gene Expression Omnibus (GEO; HNSCC, GSE181919; lnSCC, GSE148071 (luSCC samples only); cSCC, GSE218170). Data were imported, processed and analysed using the Seurat package (version 5.1) in R (version 4.3.0). Each cancer-type dataset was analysed individually. For HNSCC and cSCC datasets, cells were filtered out if they expressed fewer than 200 or greater than 7,500 unique genes or contained more than 15% mitochondrial reads. As fewer fibroblasts were present in the luSCC samples, no additional filtering cut-offs from the original sample processing were used. All datasets were normalized with the SCTransform function, regressing out the percentage of mitochondrial reads in HNSCC and luSCC datasets, and PCA was performed. The Harmony package (version 1.0.3) was used to correct for any batch effects between samples in each dataset. Dimensionality reduction and clustering were performed on the first 30 components of the harmony reduction, with a clustering resolution of 0.2. Marker genes for cell-type clusters were determined with the FindAllMarkers function using only genes detected in at least 25% of cells and had a log_2_FC greater than 0.25. The cluster containing fibroblasts identified by expression of fibroblast genes (*COL1A1*, *COL1A2*, *PI16*, *SFRP2*) were subset for further analysis. For each fibroblast dataset, cell cycle analysis was performed using the CellCycleScoring function to assign a cell cycle score to each cell, based on the expression of G2/M and S phase marker genes. Normalization, reduction and clustering were repeated as above on the fibroblast subsets, regressing out G2M and S phase scores to remove the effects of cell cycle on fibroblast clustering. For fibroblast clustering, a resolution of 0.4 was used for HNSCC data, 1.0 for luSCC data and 0.2 for cSCC data.

For each fibroblast dataset, fibroblast signatures were created for each cluster by taking the top six marker genes for each cluster (top ten genes for luSCC) that had the highest fold-change values. These signatures were used to examine fibroblast signature expression on the respective SCC spatial transcriptomics samples using the AddModuleScore function.

### Data analysis

Data collection was performed with Microsoft Excel (Microsoft 365, version 2407), and statistical analysis was performed in GraphPad Prism (version 9.2, GraphPad Software). For comparisons between two groups, Mann–Whitney tests were used, and for comparisons between three or more groups, Kruskal–Wallis with Dunn’s multiple-comparison tests were used. A *P* value < 0.05 was considered significant, after correcting for multiple testing where necessary. For human studies, statistical analysis was performed in R (version 4.3.0, RStudio Pro 2024.04.2, RStudio Inc). ssGSEA was performed using the GSEABase (version 1.50.1) and GSVA (version 1.36.3) packages in R. Survival analysis was performed using survival (version 3.1–12) and survminer (version 0.4.9) packages. Univariate grouped survival analysis was performed with Kaplan–Meier and log-rank tests, and multivariate analyses with Cox regression models, with evaluation of the proportional-hazard assumption. Gene expression (log_2_(*x* + 1) normalized RSEM) and clinical data from the TCGA LUSC and HNSC datasets were accessed from the UCSC Xena data portal (https://xenabrowser.net/datapages/). The TNF/EMT gene signature was calculated in the TCGA HNSC primary tumour cohort using ssGSEA with a custom gene set including the 15 overlapping TNF-EMT genes from SCC RNA sequencing (Supplementary Table 1). TRACERx lung cancer data were downloaded from Zenodo (10.5281/zenodo.7819449)^68^. Public datasets were downloaded from the GEO (GSE140523, GSE202048, GSE186775, GSE84293, GSE33479, GSE94611, GSE135975 and GSE244065). Fibroblast differential expression by anatomic site was performed using the DESeq2 package in R, and differentially expressed genes were further analysed using Ingenuity Pathway Analysis software (Qiagen). Fibroblast signature score was calculated using the MCPcounter package (version 1.2.0). For array data, gene expression scores were calculated as the averaged expression of genes within a biological pathway. For a list of gene-set pathways used in analyses, see Supplementary Table 16.

### Reporting summary

Further information on research design is available in the Nature Portfolio Reporting Summary linked to this article.

## Supplementary information

Supplementary Information Supplementary Figs. 1–3.

Reporting Summary

Supplementary Tables 1–16 Supplementary Table 1. Differentially expressed genes in SCC cell lines treated with dermal or lung fibroblast secretomes. Supplementary Table 2. Differentially expressed genes in SCC cell lines treated with dermal or oral fibroblast secretomes. Supplementary Table 3. Differentially expressed genes between dermal and oral or lung fibroblasts. Supplementary Table 4. Fibroblast secretome lipidomics. Supplementary Table 5. Predicted upstream regulators of genes differentially expressed in SCC cells treated with dermal or oral fibroblasts. Supplementary Table 6. Top 20 marker genes of spatial transcriptomics cell signatures in oSCC tumours. Supplementary Table 7. Fibroblast cluster markers in HNSCC single-cell RNA sequencing. Supplementary Table 8. Differentially expressed genes in lung SCC cell line SKMES1 treated with lung fibroblast secretome. Supplementary Table 9. Ingenuity Pathway Analysis of differentially expressed genes in luSCC cells treated with lung fibroblast secretome. Supplementary Table 10. Top 20 marker genes of spatial transcriptomics cell signatures in luSCC tumours. Supplementary Table 11. Gene-set enrichment analysis of maker genes in spatial transcriptomics cluster 7. Supplementary Table 12. Gene-set enrichment analysis of maker genes in high cholesterol synthesis epithelial clusters in luSCC spatial transcriptomics. Supplementary Table 13. Fibroblast cluster markers in luSCC single-cell RNA sequencing. Supplementary Table 14. Top 20 marker genes of spatial transcriptomics cell signatures in cSCC tumours. Supplementary Table 15. Fibroblast cluster markers in cSCC single-cell RNA sequencing. Supplementary Table 16. Gene sets used in analyses

Supplementary Data 1 Supplementary Figs. 1–3 source data.

## Source data

Source Data Fig. 1 Fig. 1 statistical source data.

Source Data Fig. 2 Fig. 2 statistical source data.

Source Data Fig. 3 Fig. 3 statistical source data.

Source Data Fig. 3 Fig. 3i unprocessed western blots.

Source Data Fig. 4 Fig. 4 statistical source data.

Source Data Fig. 5 Fig. 5 statistical source data.

Source Data Fig. 6 Fig. 6 statistical source data.

Source Data Fig. 7 Fig. 7 statistical source data.

Source Data Fig. 8 Fig. 8 statistical source data.

Source Data Extended Data Fig. 1 Extended Data Fig. 1 statistical source data.

Source Data Extended Data Fig. 2 Extended Data Fig. 2 statistical source data.

Source Data Extended Data Fig. 2 Extended Data Fig. 2f unprocessed western blots.

Source Data Extended Data Fig. 3 Extended Data Fig. 3 statistical source data.

Source Data Extended Data Fig. 3 Extended Data Fig. 3i unprocessed western blots.

Source Data Extended Data Fig. 4 Extended Data Fig. 4 statistical source data.

Source Data Extended Data Fig. 6 Extended Data Fig. 6 statistical source data.

Source Data Extended Data Fig. 7 Extended Data Fig. 7 statistical source data.

Source Data Extended Data Fig. 7 Extended Data Fig. 7a unprocessed western blots.

Source Data Extended Data Fig. 8 Extended Data Fig. 8 statistical source data.

Source Data Extended Data Fig. 10 Extended Data Fig. 10 statistical source data.

## Data Availability

Spatial transcriptomic and RNA-sequencing data generated in this study have been uploaded to the GEO under accession numbers GSE321832, GSE320602 and GSE322745. Source data are provided with this paper.

## Code availability

Scripts used for analysis are available on Zenodo (10.5281/zenodo.18790615)^69^.
