# Supplementary material for: Tissue-specific fibroblast lipid cues impose the rate of epithelial cancer invasion
Source: Nat Metab. 2026 Apr 27;8(5):1149–72. doi: 10.1038/s42255-026-01514-y (PMC13218938; doi:10.1038/s42255-026-01514-y)
Supplement: Supplementary file 1 — Supplementary Figs. 1–3. [file 42255_2026_1514_MOESM1_ESM.pdf]

nature metabolism

Supplementary information https://doi.org/10.1038/s42255-026-01514-y

Tissue-specific fibroblast lipid cues impose the rate of epithelial cancer invasion

In the format provided by the authors and unedited

Supplementary Figure 1

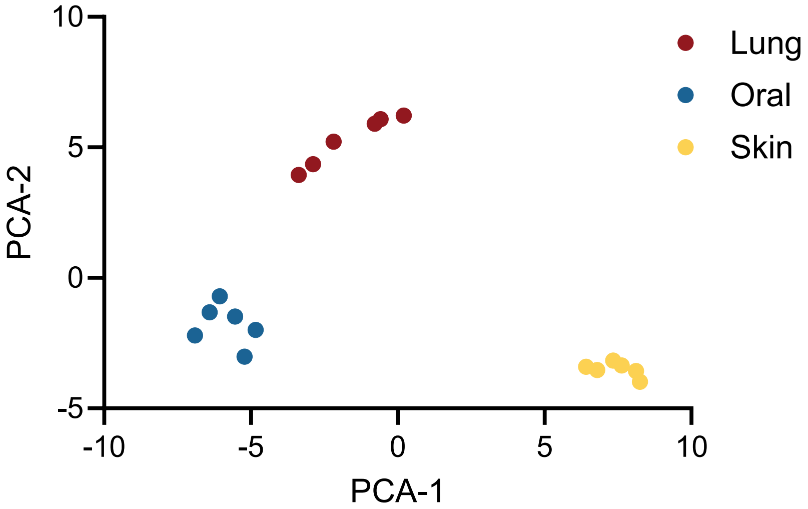

Supplementary Figure 1. Principal component analysis (PCA) plot of lipidomics from freshly collected mouse skin (yellow), oral (blue) and lung (red) tissue (n=6, two independent tissue samples from three mice).

Supplementary Figure 2

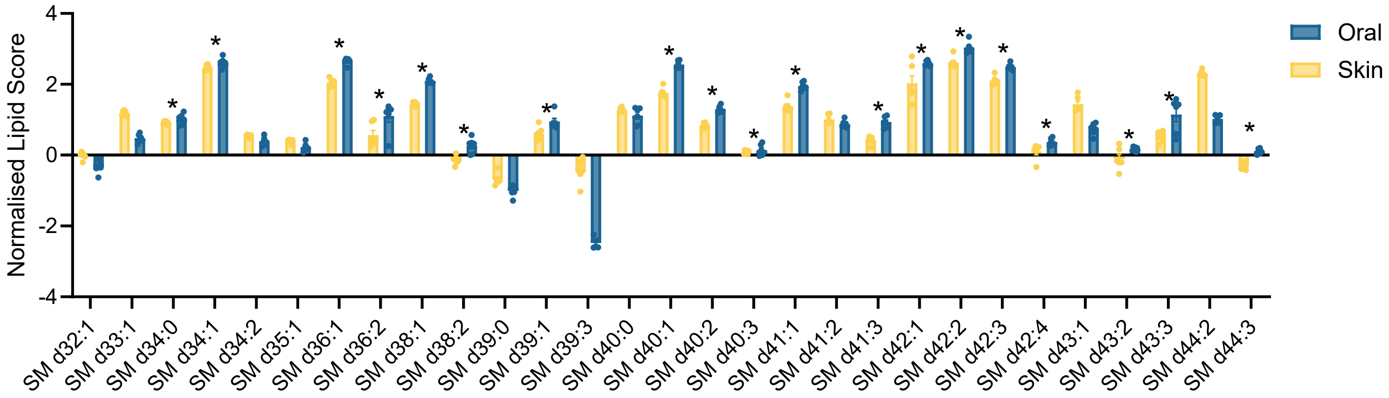

Supplementary Figure 2. Lipidomics of sphingomyelin (SM) species in mouse whole skin (yellow) and oral (blue) tissue. Asterisk denotes SM species higher in oral tissue than skin. Data represents normalised lipid scores relative to the median (n=6, two independent tissue samples from three mice). Bar plots: Mean ± standard error of the mean

Supplementary Figure 3

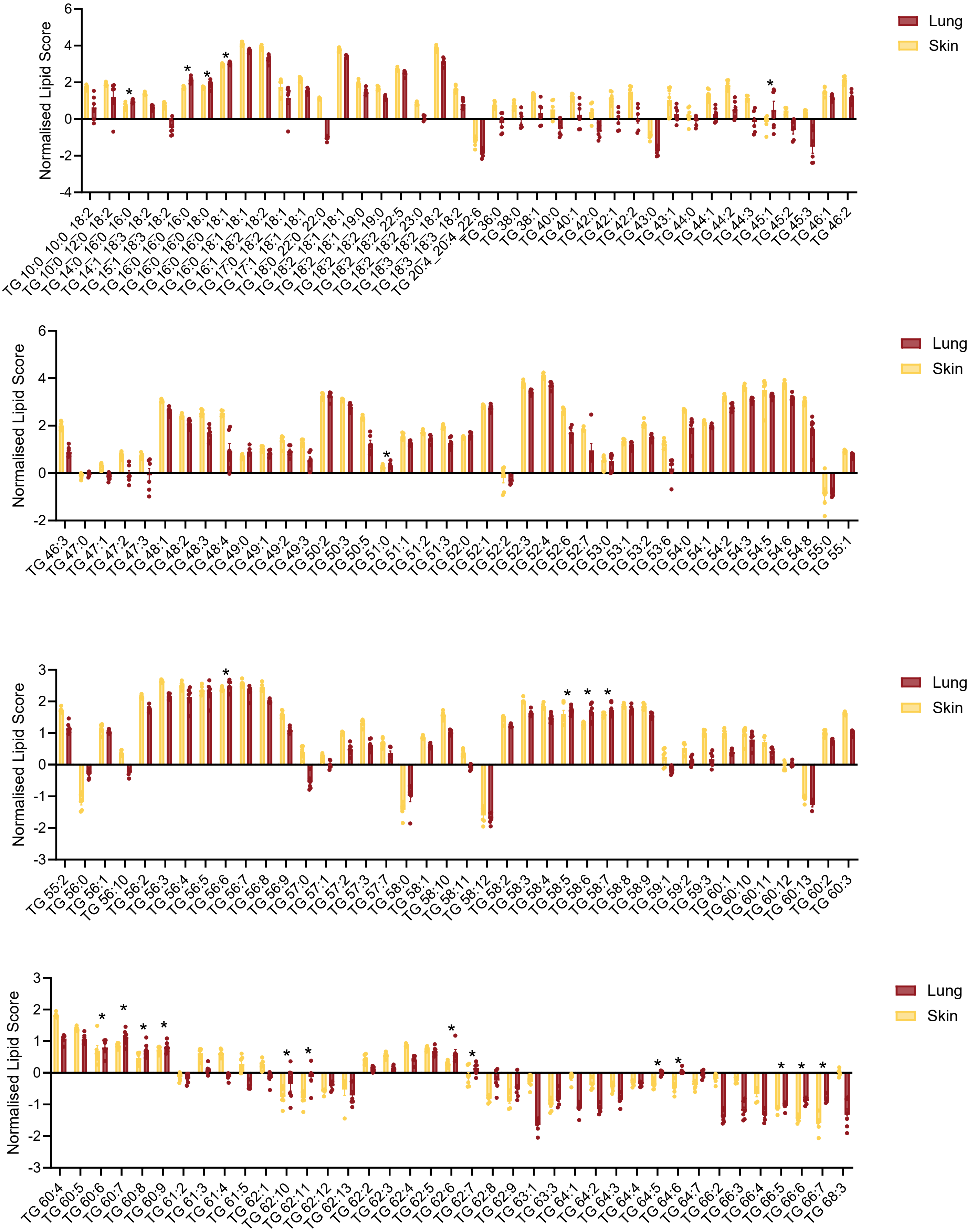

Supplementary Figure 3. Lipidomics of triglyceride (TG) species in mouse whole skin (yellow) and lung (red) tissue. Asterisk denotes TG species higher in lung tissue than skin. Data represents normalised lipid scores relative to the median (n=6, two independent tissue samples from three mice). Bar plots: Mean ± standard error of the mean.
