# Supplementary material for: Tissue-specific fibroblast lipid cues impose the rate of epithelial cancer invasion
Source: Nat Metab. 2026 Apr 27;8(5):1149–72. doi: 10.1038/s42255-026-01514-y (PMC13218938; doi:10.1038/s42255-026-01514-y)
Supplement: Supplementary file 2 — Reporting Summary [file 42255_2026_1514_MOESM2_ESM.pdf]

nature portfolio

Corresponding author(s): Amaya Viros

Last updated by author(s): Feb 17, 2026

# Reporting Summary

Nature Portfolio wishes to improve the reproducibility of the work that we publish. This form provides structure for consistency and transparency in reporting. For further information on Nature Portfolio policies, see our Editorial Policies and the Editorial Policy Checklist.

## Statistics

For all statistical analyses, confirm that the following items are present in the figure legend, table legend, main text, or Methods section.

| n/a | Confirmed | |
|---|---|---|
| ☐ | ☒ | The exact sample size (*n*) for each experimental group/condition, given as a discrete number and unit of measurement |
| ☐ | ☒ | A statement on whether measurements were taken from distinct samples or whether the same sample was measured repeatedly |
| ☐ | ☒ | The statistical test(s) used AND whether they are one- or two-sided<br>*Only common tests should be described solely by name; describe more complex techniques in the Methods section.* |
| ☐ | ☒ | A description of all covariates tested |
| ☐ | ☒ | A description of any assumptions or corrections, such as tests of normality and adjustment for multiple comparisons |
| ☐ | ☒ | A full description of the statistical parameters including central tendency (e.g. means) or other basic estimates (e.g. regression coefficient) AND variation (e.g. standard deviation) or associated estimates of uncertainty (e.g. confidence intervals) |
| ☐ | ☒ | For null hypothesis testing, the test statistic (e.g. *F*, *t*, *r*) with confidence intervals, effect sizes, degrees of freedom and *P* value noted<br>*Give P values as exact values whenever suitable.* |
| ☒ | ☐ | For Bayesian analysis, information on the choice of priors and Markov chain Monte Carlo settings |
| ☒ | ☐ | For hierarchical and complex designs, identification of the appropriate level for tests and full reporting of outcomes |
| ☐ | ☒ | Estimates of effect sizes (e.g. Cohen's *d*, Pearson's *r*), indicating how they were calculated |

*Our web collection on statistics for biologists contains articles on many of the points above.*

## Software and code

Policy information about availability of computer code

| | |
|---|---|
| Data collection | Data was collected using Microsoft Excel (Microsoft 365, version 2407), histology data was collected using Olympus VS200 slide scanner (Olympus) and HALO software (Indica labs, version 3.6.4134.314), spheroid invasion data was collected using EVOS XL Core, Thermo Fisher Scientific, AMEX1000, ELISA and plate assay data was collected using VarioSkan Lux plate reader (Thermo Fisher Scientific) and SkanIt Software (Research edition, Version 7.0.2), seahorse metabolic data was collected on Seahorse XFe96 Analyser system (Agilent Technologies) with Wave software (Agilent Technologies, version 2.6.1.53), immunofluorescence was collected on Opera Phenix (Perkin Elmer) with Harmony software (Perkin Elmer, Version 6.9), proliferation data was collected with IncuCyte S3 (Essen Bioscience) with IncuCyte Software (Essen Bioscience, 2020C Rev1), western blot data was collected with ChemiDoc system (BioRad), spatial transcriptomics data was collected with NovaSeq 6000 (Illumina). qPCR data was collected with QuantStudio 3 system (Thermo Fisher Scientific). |
| Data analysis | Image analysis was performed using HALO software (Indica labs, version 3.6.4134.314). Seahorse metabolism data was analysed using Wave software (Agilent Technologies, version 2.6.1.53), immunofluorescence data was analysed using Harmony software (Perkin Elmer, Version 6.9), western blot images were analysed using ImageJ software (version 1.53o), RNA sequencing analysed with FASTQC, Trim Galore v0.6.10, STAR aligner v2.5.1b and downstream analysis performed in (version 4.3.0 RStudio Pro 2024.04.2, RStudio Inc).and Ingenuity Pathway Analysis software (IPA, Qiagen). Spatial Transcriptomics data was processed with Space Ranger and analysed in R. For in vitro and in vivo studies statistical analysis was performed in GraphPad Prism (version 9.2, GraphPad Software, Inc.). Analysis of public data sets and clinical data statistical analysis was performed in R (version 4.3.0 RStudio Pro 2024.04.2, RStudio Inc). All analyses packages and software are cited in methods. |

For manuscripts utilizing custom algorithms or software that are central to the research but not yet described in published literature, software must be made available to editors and reviewers. We strongly encourage code deposition in a community repository (e.g. GitHub). See the Nature Portfolio guidelines for submitting code & software for further information.

## Data

Policy information about availability of data

All manuscripts must include a data availability statement. This statement should provide the following information, where applicable:

- Accession codes, unique identifiers, or web links for publicly available datasets
- A description of any restrictions on data availability
- For clinical datasets or third party data, please ensure that the statement adheres to our policy

Public data sets were downloaded from NCBI Gene Expression Omnibus (GEO) (GSE140523, GSE202048, GSE186775, GSE84293, GSE33479, GSE94611, GSE135975, GSE244065, GSE181919, GSE148071, GSE218170)
Data for second lung SCC sample was downloaded from BioStudies (E-MTAB-13530).
TCGA data was downloaded from the UCSC Xena data portal (https://xenabrowser.net/datapages/)
TRACERx lung cancer data was downloaded from Zenodo (https://zenodo.org/records/7819449)
The datasets generated during the current study are available in the Supplementary Tables, Spatial transcriptomic and RNA sequencing data generated in this study have been uploaded to the Gene Expression Omnibus under accession numbers GSE321832, GSE320602, and GSE322745. Source data are provided with this paper.

## Research involving human participants, their data, or biological material

Policy information about studies with human participants or human data. See also policy information about sex, gender (identity/presentation), and sexual orientation and race, ethnicity and racism.

| | |
|---|---|
| Reporting on sex and gender | We used five human samples, male, which were the ones available from Biobank and Royal Marsden, and Northern Care Alliance NHS |
| Reporting on race, ethnicity, or other socially relevant groupings | As we analysed 1 or 2 samples per cancer group, there is no possibility to stratify by age, sex, race, ethnicity or other socially relevant characteristics. Samples were chosen by availability. |
| Population characteristics | cancer patient SCC |
| Recruitment | tissue availability two samples |
| Ethics oversight | The oral SCC human specimens analysed by spatial transcriptomics was obtained from the CCR5396 ORIGINS Study. The ORganoid GeneratioN Study for Cancer (ORIGINS, NCT05734963) was reviewed and approved by The Committee for Clinical Research at The Royal Marsden Hospital (reference CCR5396) and the North Tyneside 1 Research Ethics Committee (reference 21/NE/0096, IRAS 292105). Cutaneous SCC specimens were obtained from Salford Royal Northern Care Alliance, ethics approved by the Human Research Authority, Study title: Mechanistic differences driving distinct skin cancer subtypes IRAS project ID: 216310 REC reference: 16/LO/2098, Sponsored by the The University of Manchester The human lung SCC specimens were obtained from the Christie NHS Foundation Trust Biobank under ethical approval granted by the local Biobank committee (17_AMVI_01). |

Note that full information on the approval of the study protocol must also be provided in the manuscript.

# Field-specific reporting

Please select the one below that is the best fit for your research. If you are not sure, read the appropriate sections before making your selection.

☒ Life sciences ☐ Behavioural & social sciences ☐ Ecological, evolutionary & environmental sciences

For a reference copy of the document with all sections, see nature.com/documents/nr-reporting-summary-flat.pdf

# Life sciences study design

All studies must disclose on these points even when the disclosure is negative.

| | |
|---|---|
| Sample size | In vitro: Two independent cell lines per SCC type, three independent cells per fibroblast cell type. The number of SCC lines was limited by their scarcity and availability. The number of 3 patient fibroblasts was also selected by patient sample availability. Secretomes were collected from all fibroblast cell lines in a minimum of biological duplicate. Organotypics were made with independent duplicates for each SCC cell type. Spheroids were performed with all SCC cell lines with a minimum n=8, using 3 independent fibroblast cell line secretomes per condition, run across two independent experiments. RNA sequencing was performed on independent biological duplicates for all cell lines sequenced. Seahorse experiments were run with 5 independent replicates per cell line per condition.<br>In vivo: Group sizes (n = 6-8 per group) were selected based on prior in vivo tumour growth experiments from our laboratory and published studies demonstrating that this number provides sufficient power to detect biologically meaningful differences in tumour burden while minimising animal use. Assuming a 35% difference in tumour volume between groups, a standard deviation of 25%, a two-sided α of 0.05 and 80% power, a minimum of 6 mice per group was required. To account for potential attrition, 7–8 mice per group were used in some experiments.<br>Public data sets: All relevant data points were included. |

| | |
|---|---|
| Data exclusions | Two mice were excluded from analyses in vivo study as the did not develop tumours after subcutaneous injection. |
| Replication | in vitro: Three independent cell lines were used for each fibroblast type and two independent cell lines per SCC group. All experiments were performed with at least two independent fibroblasts per group in biological duplicate as indicated. Spheroid assays were replicates across two independent experiments. All replication experiments were successful. |
| Randomization | Treatments of mice with statins were randomized within each cage, assessment of histology was done by TB, LM and AV, blinded for group. |
| Blinding | All in vivo tumour volume measurements were taken by a technician who was blind to the experimental rationale. |

# Reporting for specific materials, systems and methods

We require information from authors about some types of materials, experimental systems and methods used in many studies. Here, indicate whether each material, system or method listed is relevant to your study. If you are not sure if a list item applies to your research, read the appropriate section before selecting a response.

## Materials & experimental systems

| n/a | Involved in the study |
|---|---|
| ☐ | ☒ Antibodies |
| ☐ | ☒ Eukaryotic cell lines |
| ☒ | ☐ Palaeontology and archaeology |
| ☐ | ☒ Animals and other organisms |
| ☒ | ☐ Clinical data |
| ☒ | ☐ Dual use research of concern |
| ☒ | ☐ Plants |

## Methods

| n/a | Involved in the study |
|---|---|
| ☒ | ☐ ChIP-seq |
| ☒ | ☐ Flow cytometry |
| ☒ | ☐ MRI-based neuroimaging |

## Antibodies

| | |
|---|---|
| Antibodies used | Ki67, Agilent, M7240, 1:200 dilution<br>Vinculin, Abcam, ab129002, 1:10,000 dilution<br>SPHK1, Cell Signalling, 12071, 1:1000 dilution<br>Human mitochondria, Abcam, ab92824, 1ug/ml dilution<br>IL-6, Abcam, ab233706, 1:200 dilution<br>SREBP2, Abcam, ab30682, 1:1000 dilution<br>TATA Binding protein, Abcam, ab220788, 1:2000 dilution<br>Alpha smooth muscle actin, Abcam, ab7817, 1:200 dilution<br>APOE, Abcam, ab183597, 1:1000 dilution<br>S1P, Echelon Biosciences, Z-P300, 1:500 dilution<br>goat anti-rabbit Alexa Fluor 488, Thermo Fisher, A32731, 1:1000 dilution<br>goat anti-mouse Alexa Fluor 488, Thermo Fisher, A32723, 1:1000 dilution<br>goat anti-rabbit Alexa Fluor 555, Thermo Fisher, A32732, 1:1000 dilution<br>STAT3, 9139, Cell Signalling, 1:1000 dilution<br>phosphor-STAT3 Tyr705, 9145, Cell Signalling, 1:500 dilution<br>B-actin, ab8226, Abcam, 1:10,000 dilution<br>Anti-Rabbit IgG, HRP-linked Antibody, Cell Signalling, 7074, 1:5000 dilution<br>Anti- Mouse IgG, HRP-linked Antibody, Cell Signalling, 7076, 1:5000 dilution |
| Validation | All antibodies used were validated for the specific application and species by the manufacturers and all antibodies were used according the manufactures instructions only for the validated species and application. Additionally, we validated staining in human tonsil as a positive control alongside an isotype negative control antibody. |

## Eukaryotic cell lines

Policy information about cell lines and Sex and Gender in Research

| | |
|---|---|
| Cell line source(s) | IC1 (male) and IC19 (male) were provided by Prof. Catherine Harwood from Queen Mary University London<br>SKMES1 (male) and H520 (male) were acquired from Dr Carlos Lopez-Garcia, Cancer Research UK Manchester Institute.<br>UMSCC01 (male) and FADU (male) were acquired from Prof Catherine West, University of Manchester<br>Normal lung fibroblasts were purchased from Lonza (CC-2512) (2x female, 1x male)<br>Two oral fibroblast cell lines (male) were purchased from Generon (CTICC1.8.2)<br>One oral fibroblast cell line was provided by Prof Caroline Gaudy, Aix-Marseille Université<br>Dermal fibroblasts were established from redundant skin acquired during surgical resection of the wide local excision of healthy skin. Three dermal fibroblast cell lines were from female donors. |
| Authentication | Cancer cell lines were authenticated by STR profiling |

| | |
|---|---|
| Mycoplasma contamination | All the cell lines tested negative for Mycoplasma in monthly testing by LookOut Mycoplasma PCR kits |
| Commonly misidentified lines (See ICLAC register) | No commonly misidentified cell lines were used in this study |

## Animals and other research organisms

Policy information about studies involving animals; ARRIVE guidelines recommended for reporting animal research, and Sex and Gender in Research

| | |
|---|---|
| Laboratory animals | Mus musculus, NSG, 8-12 weeks old; C57BL/6 8 weeks old; Apoe -/- C57BL/6 8 weeks old |
| Wild animals | Study did not involve wild animals |
| Reporting on sex | All mice used in subcutaneous tumour models were female. C57BL/6 WT and Apoe -/- for fibroblast extraction were male |
| Field-collected samples | Study did not involve samples collected from the field. |
| Ethics oversight | All procedures involving animals were performed under the Home Office approved project license PPL PP0466403, and UK Home Office regulations under the Animals (Scientific Procedures) Act 1986. The study received ethical approval by the Cancer Research UK Manchester Institute's Animal Welfare and Ethics Review Body (AWERB). |

Note that full information on the approval of the study protocol must also be provided in the manuscript.

## Plants

| | |
|---|---|
| Seed stocks | *Report on the source of all seed stocks or other plant material used. If applicable, state the seed stock centre and catalogue number. If plant specimens were collected from the field, describe the collection location, date and sampling procedures.* |
| Novel plant genotypes | *Describe the methods by which all novel plant genotypes were produced. This includes those generated by transgenic approaches, gene editing, chemical/radiation-based mutagenesis and hybridization. For transgenic lines, describe the transformation method, the number of independent lines analyzed and the generation upon which experiments were performed. For gene-edited lines, describe the editor used, the endogenous sequence targeted for editing, the targeting guide RNA sequence (if applicable) and how the editor was applied.* |
| Authentication | *Describe any authentication procedures for each seed stock used or novel genotype generated. Describe any experiments used to assess the effect of a mutation and, where applicable, how potential secondary effects (e.g. second site T-DNA insertions, mosiacism, off-target gene editing) were examined.* |
