# Supplementary material for: Tissue-specific fibroblast lipid cues impose the rate of epithelial cancer invasion
Source: Nat Metab. 2026 Apr 27;8(5):1149–72. doi: 10.1038/s42255-026-01514-y (PMC13218938; doi:10.1038/s42255-026-01514-y)

Figure 3i – STAT3/B-actin (left blot), pSTAT3/B-actin (right blot)

Sample order identical on both blots

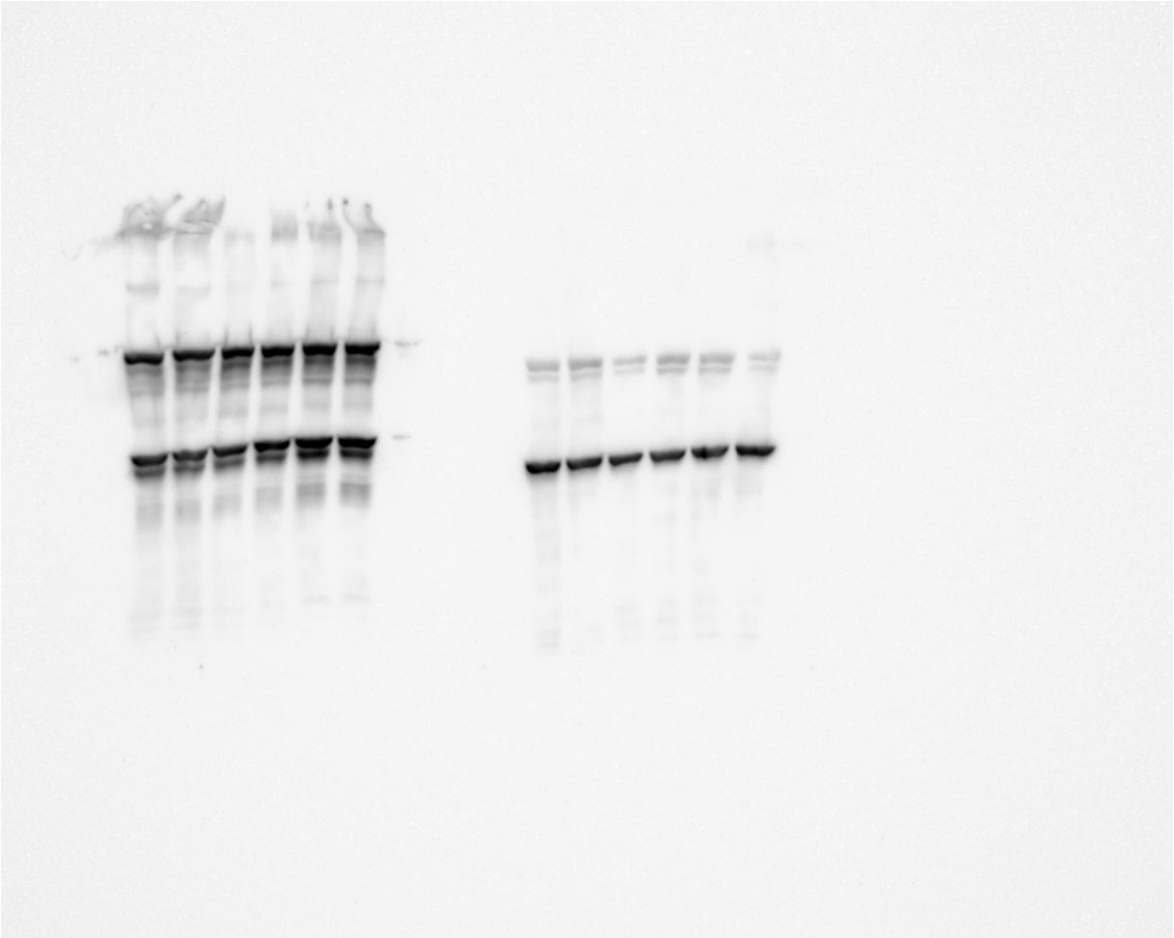

Figure 3i – gel with ladder (shorter exposure)

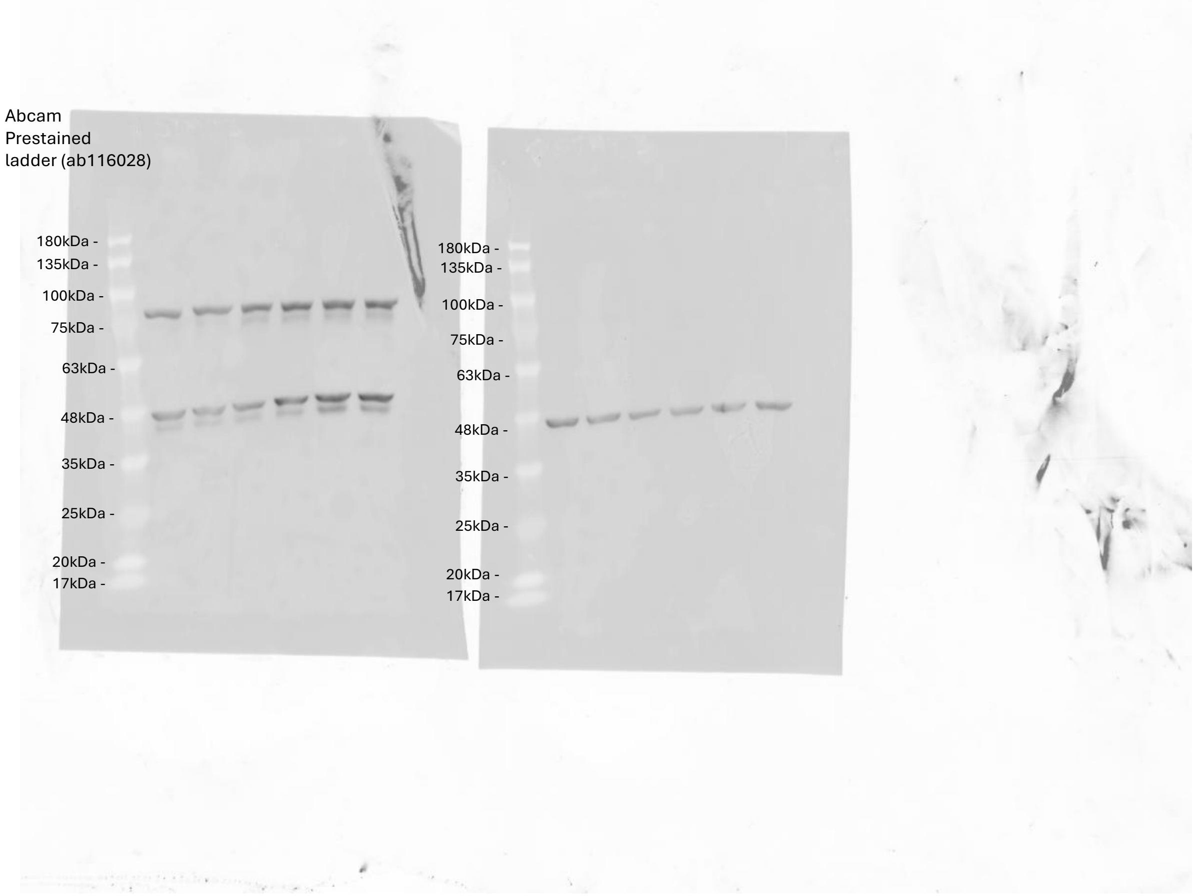

Supplement: Supplementary file 8 — Fig. 3i unprocessed western blots. [file 42255_2026_1514_MOESM8_ESM.pdf]
