# Supplementary material for: Tissue-specific fibroblast lipid cues impose the rate of epithelial cancer invasion
Source: Nat Metab. 2026 Apr 27;8(5):1149–72. doi: 10.1038/s42255-026-01514-y (PMC13218938; doi:10.1038/s42255-026-01514-y)

Extended Data Figure 2f – STAT3/B-actin (left blot), pSTAT3/B-actin (right blot)

Sample order identical on both blots

Low exposure used for STAT3 and B-actin

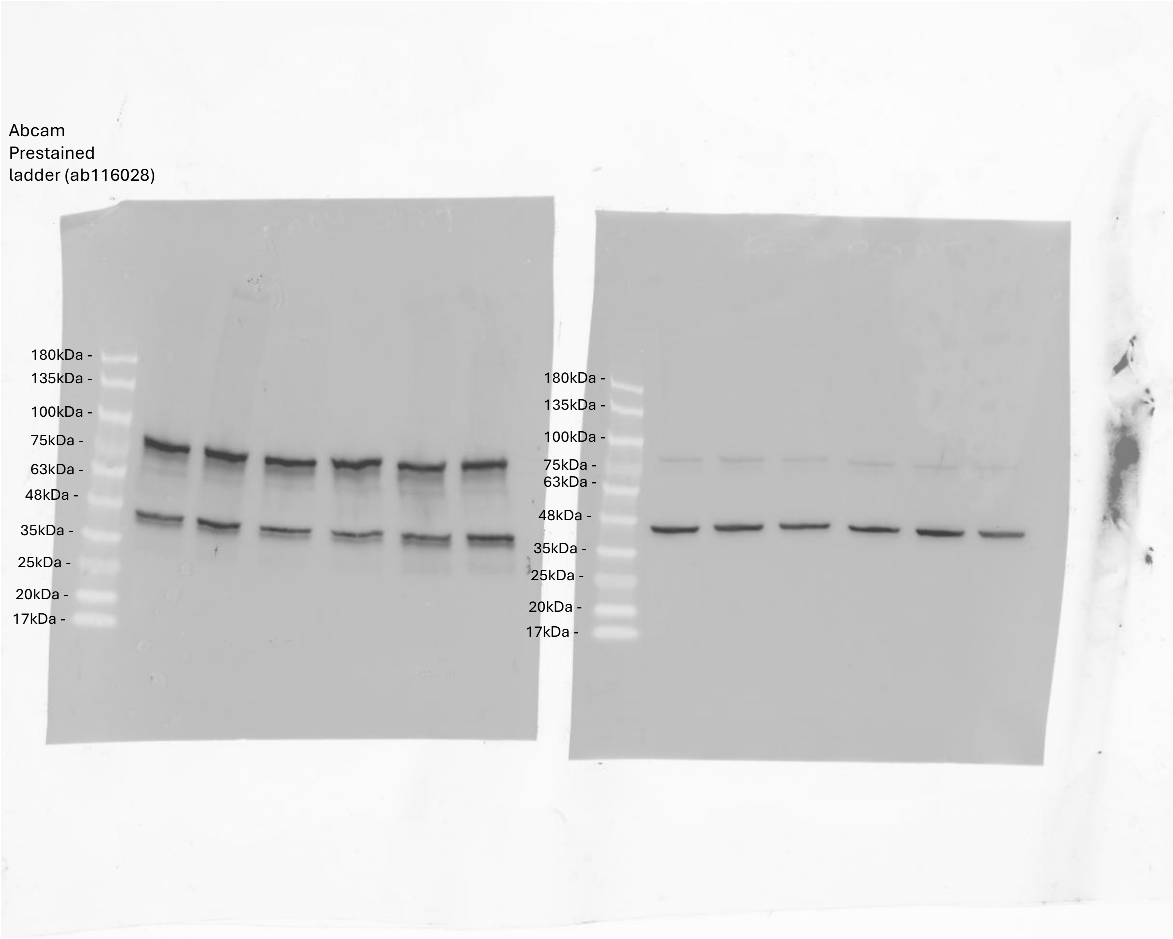

Higher exposure used for pSTAT3 and B-actin

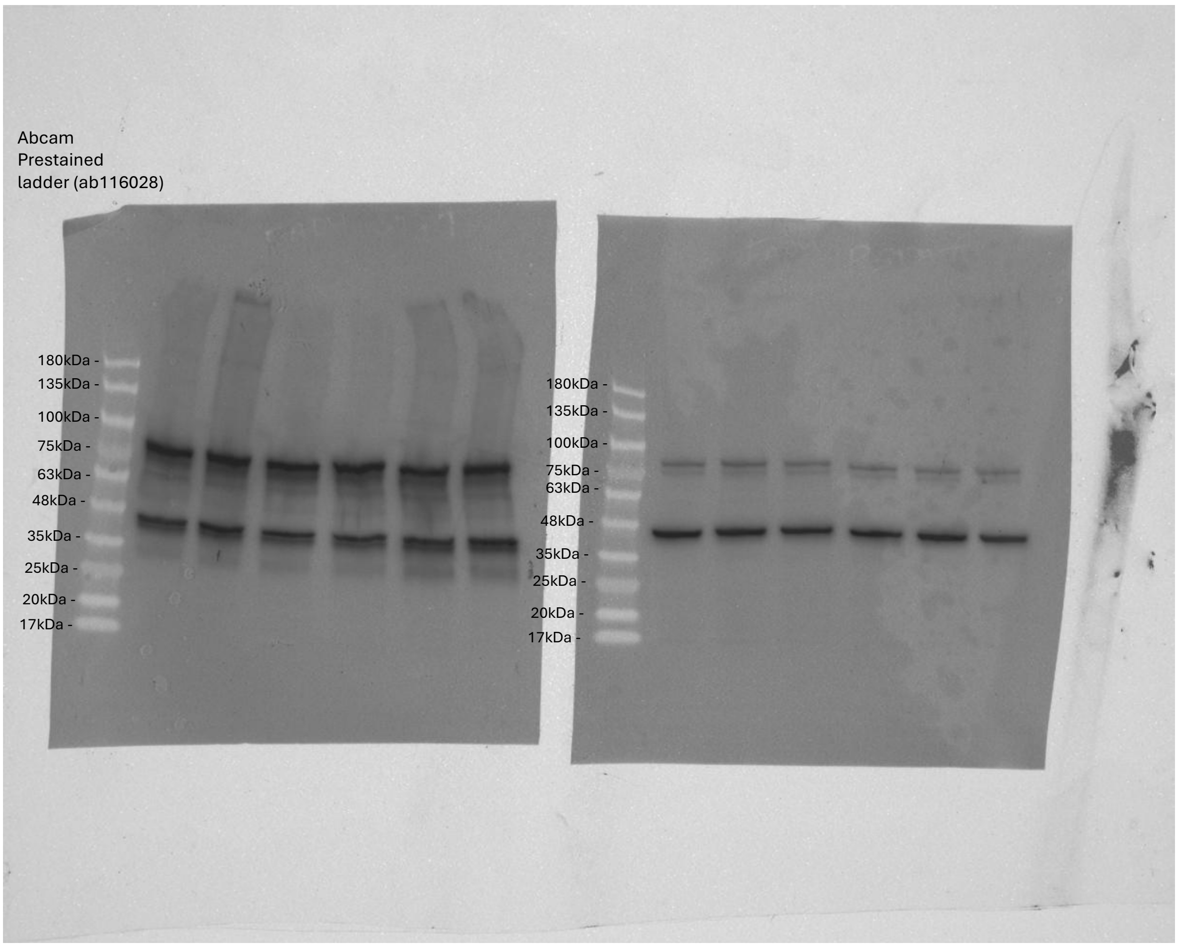

Supplement: Supplementary file 16 — Extended Data Fig. 2f unprocessed western blots. [file 42255_2026_1514_MOESM16_ESM.pdf]
