# Supplementary material for: Tissue-specific fibroblast lipid cues impose the rate of epithelial cancer invasion
Source: Nat Metab. 2026 Apr 27;8(5):1149–72. doi: 10.1038/s42255-026-01514-y (PMC13218938; doi:10.1038/s42255-026-01514-y)

Extended Data Figure 3i – Vinculin, SPHK1

Low exposure used for Vinculin

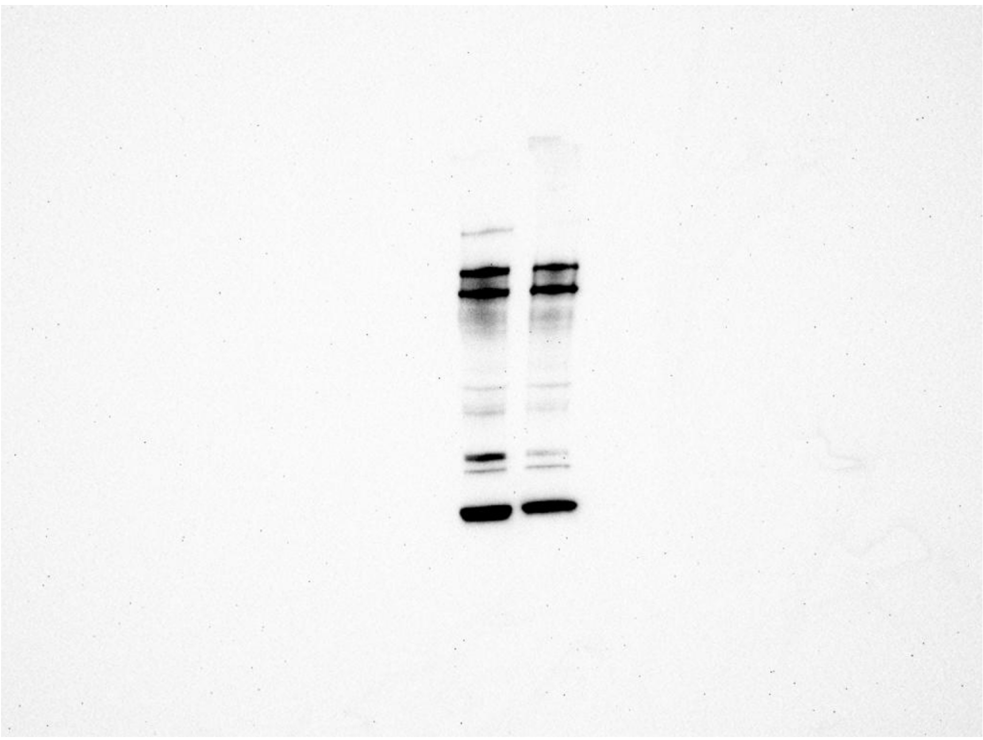

Higher exposure used for SPHK1

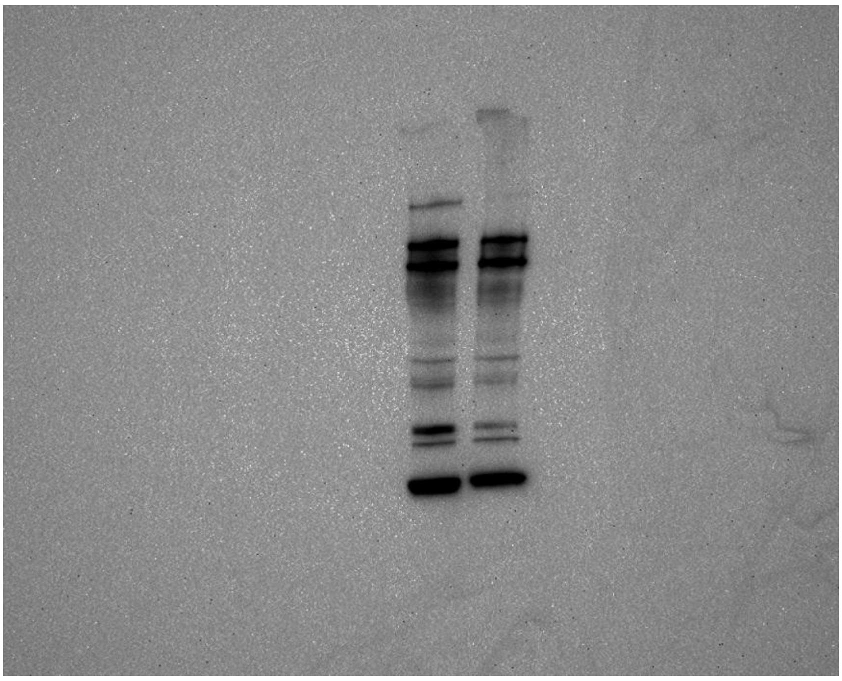

With ladder

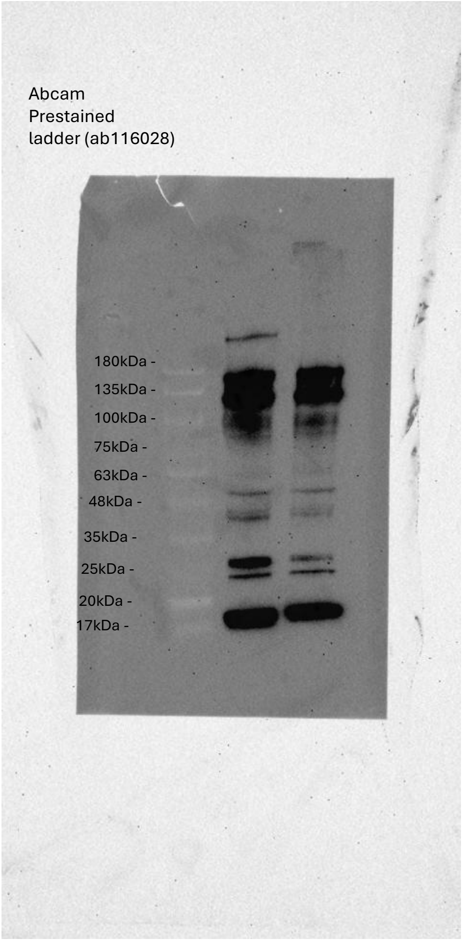

Supplement: Supplementary file 18 — Extended Data Fig. 3i unprocessed western blots. [file 42255_2026_1514_MOESM18_ESM.pdf]
