# Supplementary material for: Tissue-specific fibroblast lipid cues impose the rate of epithelial cancer invasion
Source: Nat Metab. 2026 Apr 27;8(5):1149–72. doi: 10.1038/s42255-026-01514-y (PMC13218938; doi:10.1038/s42255-026-01514-y)
Supplement: Supplementary file 22 — Extended Data Fig. 7a unprocessed western blots. [file 42255_2026_1514_MOESM22_ESM.pdf]

Extended Data Figure 7a – SREBP2, TATA BP

Sample order identical on images

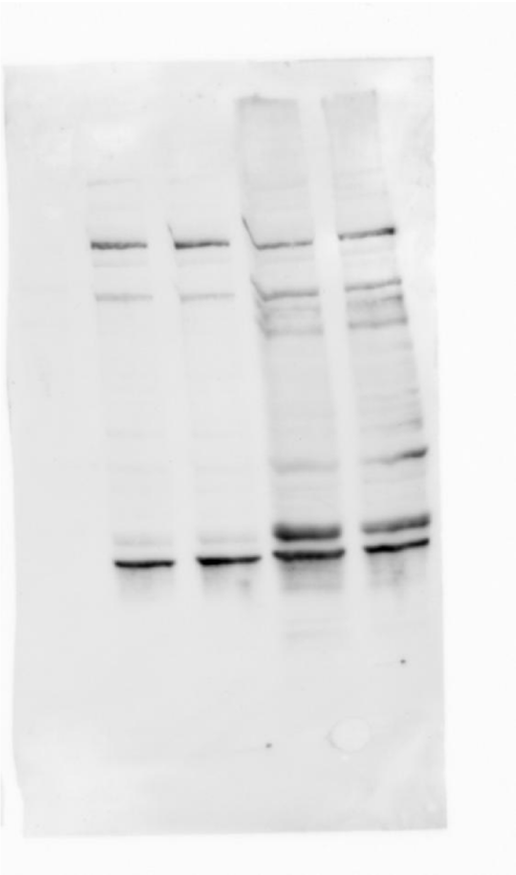

With ladder, without TATA BP (same blot)

180kDa -
135kDa -
100kDa -
75kDa -
63kDa -
48kDa -
35kDa -
25kDa -
20kDa -
17kDa -

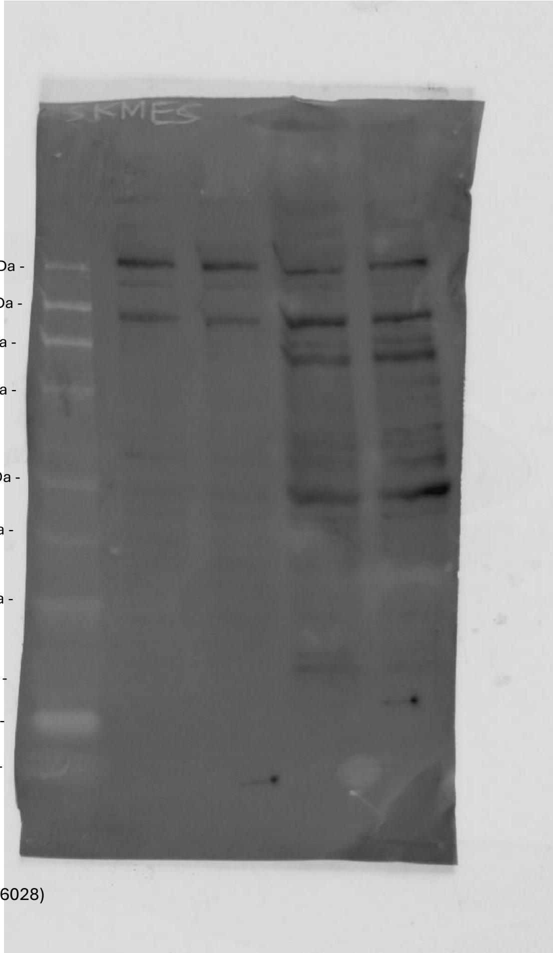

Abcam
Prestained
ladder (ab116028)
